# Hydroxyl transfer *versus* cyclization reaction in the gas phase: Sequential loss of NH_3_ and CH_2_CO from protonated phenylalanine derivatives

**DOI:** 10.3389/fchem.2022.1094329

**Published:** 2023-01-09

**Authors:** Mingyu Zheng, Xiaoping Zhang, Yihao Cheng, Lili Sun, Xinglei Zhang

**Affiliations:** Jiangxi Key Laboratory for Mass Spectrometry and Instrumentation, East China University of Technology, Nanchang, China

**Keywords:** hydroxyl transfer, electrospray ionization mass spectrometry, gas-phase reaction, phenylalanine, melphalan

## Abstract

Collisional activation of protonated phenylalanine derivatives deamination products leads to hydroxyl skeletal rearrangement *versus* cyclization reaction, and to form hydroxylbenzyl cation *via* elimination of CH_2_CO. To better clarify this unusual fragmentation reaction, accurate mass measurements experiments, native isotope experiments, multiple-stage mass spectrometry experiments, different substituents experiments, and density functional theory (DFT) calculations were carried out to investigate the dissociation mechanistic pathways of protonated phenylalanine derivatives deamination products. In route 1, a three-membered ring-opening reaction and a 1,3-hydroxyl transfer (from the carbonyl carbon atom to the interposition carbon atom of carbonyl) occurs to form 3-hydroxy-1-oxo-3-phenylpropan-1-ylium, followed by dissociation to lose CH_2_CO to give hydroxy (phenyl)methylium. In route 2, a successive cyclization rearrangement reaction and proton transfer occur to form a 2-hydroxylphenylpropionyl cation or protonated 2-hydroxy-4H-benzopyran, followed by dissociation to lose CH_2_CO or CH≡COH to give 2-hydroxylbenzyl cation. In route 3, a successive hydroxyl transfer (from the carbonyl carbon atom to the ortho carbon atom on benzene) and two stepwise proton transfer (1,2-proton transfer to the ipso-carbon atom of the phenyl ring followed by 1,3-proton transfer to the ortho carbon atom of carbonyl) occurs to form a 2-hydroxylphenylpropionyl cation, which subsequently dissociates to form 2-hydroxylbenzyl cation by elimination of CH_2_CO. DFT calculations suggested that route 1 was more favorable than route 2 and route 3 from a thermodynamic point of view.

## Introduction

Electrospray ionization mass spectrometry (ESI-MS) is not only a versatile technique for the analysis of a wide range of compounds but also has proven to be a powerful tool for the investigation of molecular structure, mechanism, and dynamic (Gronert, 2001; Indelicato et al., 2021; Li et al., 2021; Qiu et al., 2022). In particular, tandem mass spectrometry can offer abundant fragmentation data for structure elucidation, which is acted as a small “gas-phase chemistry laboratory”. Several specific rearrangement reactions have been reported in tandem mass spectrometry, including hydride transfer (Chai et al., 2010), benzyl cation transfer (Sun et al., 2012; Li et al., 2014; Paulose et al., 2015), sulfonyl cation transfer (Wang et al., 2016), methyl transfer (Kshirsagar and Argade, 2009; Ren et al., 2019), sulfur transfer (Zhang et al., 2019), halogen transfer (Chai et al., 2016; Zhang and Cheng, 2017), *etc.* Mastering these specific rearrangement transfer reactions can help to elucidate the structural information of compounds, enrich the content of gas-phase ion chemistry, and also help to discover new pathways of drug metabolism.

Phenylalanine derivatives are an important class of functionally active substances that are frequently involved in various metabolisms in the body, and their metabolites are hydroxylated products (Matthews, 2007). Melphalan, a phenylalanine derivative containing bis-β-chloroethylamine groups, plays an important role in the development of therapeutic approaches for tumors as well as cancer (Musto and D'Auria, 2007). Investigation of the fragmentation mechanism of phenylalanine derivatives can promote structure elucidation and help new metabolic pathways be discovered. The dissociation reactions of protonated amino acids such as phenylalanine have attracted considerable attention using tandem mass spectrometry (Perera et al., 2002; Lioe and O’Hair, 2005). It has been shown that protonated amino acids fragment predominantly to form their respective iminium ions by concomitant loss of H_2_O and CO, giving rise to very simple CID mass spectra.

In this study, we report the hydroxy transfer reaction of phenylalanine derivatives in detail. Melphalan was selected as a model to perform a detailed mechanistic investigation of the fragmentation reaction. Collisional activation of protonated melphalan occurred hydroxyl transfer, which generated hydroxybenzyl cation. Density functional theory (DFT) calculations and substituent effects were also implemented for the mechanism research.

## Experimental section

### Chemicals and material

**SCHEME 1:**
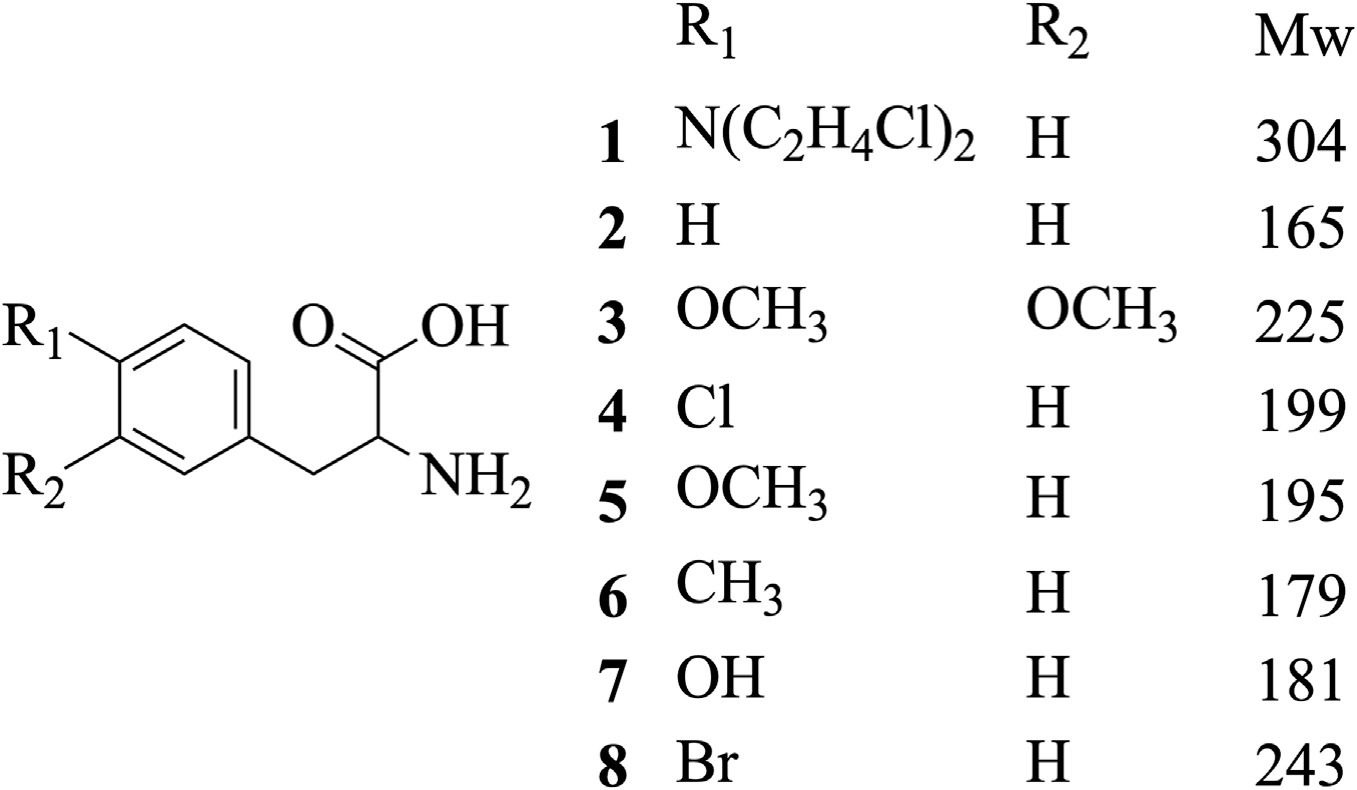
Structures of phenylalanine derivatives. (Mw: molecular weight)

Methanol HPLC grade was purchased from Sigma-Aldrich (St. Louis, MO, United States). Melphalan (compound **1**) and phenylalanine (compound **2**) were purchased from J&K Scientific Ltd (Shanghai, China) with a purity>99%. 3,4-Dimethoxyphenylalanine (compound **3**), 4-chlorophenylalanine (compound **4**), 4-methoxyphenylalanine (compound **5**), 4-methylphenylalanine (compound **6**), tyrosine (compound **7**), 4-bromophenylalanine (compound **8**) and 2-hydroxylbenzylamine were purchased from Sinopharm Chemical Reagent Co., Ltd. Their structures of them are listed in Scheme 1. Experimental ultrapure water produced by Millipore ultrapure water system.

### Mass spectrometry

The samples were analyzed on an LTQ-XL advantage IT-MS (Thermo Scientific, San Jose, CA, United States) and an Orbitrap-XL mass spectrometer (Thermo Scientific, San Jose, CA, United States) using a homemade ESI interface in the positive ion mode. The optimized ESI source conditions were as follows: the capillary temperature at 250°C, the capillary voltage at 19 V; the tube lens at 90 V, the nebulizing gas (N_2_), and 25 arbitrary units (a.u.). The samples were injected by a syringe pump at a flow rate of 5 μL/min. Other LTQ-XL parameters were automatically optimized by the system. The Orbitrap-XL mass spectrometer instrument was operated at a high resolution of up to 100,000. The collision-induced dissociation (CID) MS data were obtained with helium as the collision gas. In CID-MS experiments, the desired precursor ions were isolated with an isolation window of 1.0 m*/z* unit. The CID-MS spectra of the protonated molecules were obtained by activation of the precursor ions at the normalized collision energy of 20–30%.

### Theoretical calculations

Theoretical calculations were performed using the Gaussian 09 program (Frisch et al., 2010). The geometries such as reactants, intermediates, transition states (TSs), and products were optimized by the density functional theory (DFT) method at the B3LYP/6-31+G (d,*p*) level. TSs were obtained by relaxed PES scans, in which a bond length was scanned to search a first-order saddle point, and subsequently optimizing the corresponding TS. Then, the relevant TS structures were searched and optimized by either TS or QST2. All TSs were confirmed by the presence of a single imaginary vibrational frequency using intrinsic reaction coordinate (IRC) method. IRC calculations at the same level of theory were performed on each transition state to further confirm that the optimized TS structures were connected to the correct reactants and products by the steepest descend path. Vibrational frequencies of all the key species were calculated at the same level of theory. Full structural details and energies of all structures involved are available in the supplementary material. The energies discussed here are the sum of electronic and thermal free energy.

## Result and discussion

### Fragmentation behavior of protonated melphalan

**FIGURE 1:**
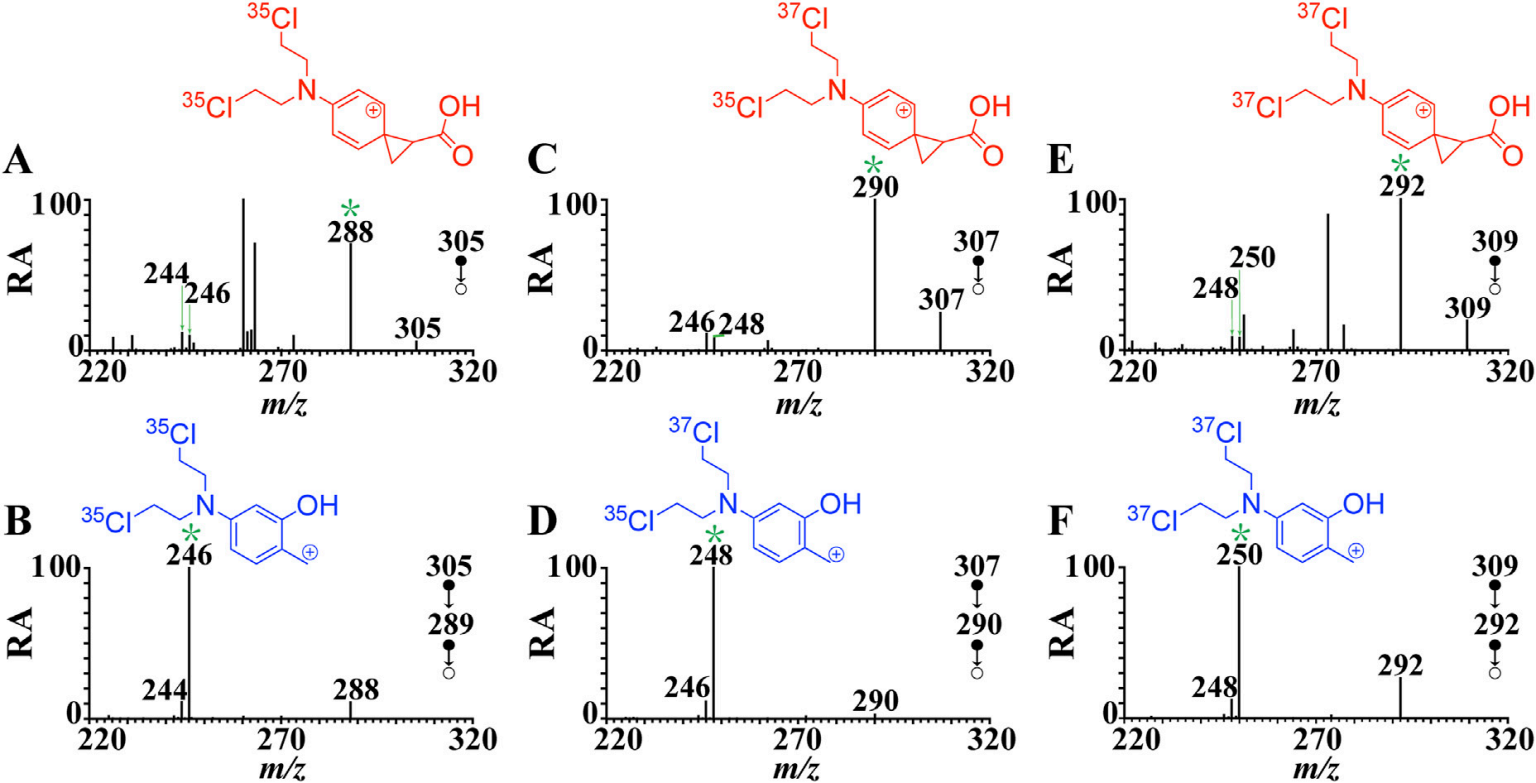
CID mass spectra of [melphalan + H]^+^ at the normalized collision energy of 20% **(A)** MS^2^ spectrum of [melphalan**-** ^35^Cl^35^Cl + H ]^+^ (*m/z* 305→), **(B)** MS^3^ spectrum of [melphalan**-** ^35^Cl^35^Cl + H]^+^ (*m/z* 305→*m/z* 288→), **(C)** MS^2^ spectrum of [melphalan **-** ^37^Cl^35^Cl + H]^+^ (*m/z* 307→), **(D)** MS^3^ spectrum of [melphalan **-** ^37^Cl^35^Cl + H]^+^ (*m/z* 307→*m/z* 290→) **(E)** MS^2^ spectrum of [melphalan **-** ^37^Cl^37^Cl + H]^+^ (*m/z* 309→), **(F)** MS^3^ spectrum of [melphalan **-** ^37^Cl^37^Cl + H]^+^ (*m/z* 309→*m/z* 292→). The structures in the figure correspond to the structure of the ion marked with an asterisk.

The gas phase hydroxyl transfer rearrangement reaction was explored by investigating the MS fragmentation behavior of protonated phenylalanine derivatives (Scheme 1). Melphalan (compound **1**) was selected as a model to perform a detailed investigation. The tandem mass spectrum of protonated melphalan (***a***, *m/*z 305) shown in Figure 1A reveals the generation of a major fragment ion at *m/z* 288, corresponding to ***b*** *via* a neutral loss of 17 Da (NH_3_). Fragment ions at *m/z* 246 and *m/z* 244 are attributed to the elimination of (NH_3_ + CO_2_) and (NH_3_ + CH_2_CO) of the precursor ion, respectively, which will be discussed in detail in the following sections.

### Fragmentation behavior of protonated melphalan deamination product

The characteristic fragment ion at *m/*z 246 can only be interpreted as a result of the (NH_3_ + CH_2_CO) elimination. To verify the formation mechanism of ion at *m/z* 246, MS^3^ experiments were performed. As shown in Figure 1B, the MS^3^ spectrum of protonated melphalan (*m/z* 305→*m/z* 288→) shows fragment ions at *m/z* 246 and *m/z* 244 *via* eliminations of CH_2_CO and CO_2_, respectively. However, there is no relevant moiety of CH_2_CO in the structure of ***b*** (*m/z* 288). Thus, the generation of the ion at *m/z* 246 originated from the dissociation of ***b*** (*m/z* 288) may *via* skeletal rearrangement. As reported earlier, protonated melphalan does appear to eliminate NH_3_ and CH_2_CO. This is similar to the results of the Harrison group (Tsang and Harrison, 1976; Harrison and Ichikawa, 1980) and K. W. Michael Siu et al. (Shoeib et al., 2002; El Aribi et al., 2004). O’Hair group has also done a lot of work on the gas-phase behavior of protonated phenylalanines (Lioe and O’Hair, 2005; Lioe and O’Hair, 2007), but no such a CH_2_CO loss has been observed, possibly due to the differences on the energy transfer to the ions which depending on the different MS instruments used. Harrison’s group has proposed a mechanism for the loss of NH_3_ and CH_2_CO from protonated phenylalanine in which the hydroxide ion migrates to form Ph-CHOH^+^ ion with concomitant elimination of CH_2_CO (Tsang and Harrison, 1976). This similar mechanism was also invoked by Ichikawa and co-workers who proposed migration of the methoxy ion in the dissociation of protonated methyl cinnamate (Harrison and Ichikawa, 1980). However, few detailed studies have been conducted on the detailed mechanism of hydroxyl transfer. Are there other mechanisms such as cyclization reaction that contribute to the formation of *m/z* 246?

Here we are interested in the characteristic fragment ion *m/z* 246 marked as ***p2*** (100%). ***P2*** is 42 Da (CH_2_ = C=O or CH≡COH) less than the precursor ion ***b***. The elemental compositions of fragment ions at *m/z* 246 and *m/z* 244 were determined by accurate mass measurements performed on a high-resolution Orbitrap-XL mass spectrometer (Supplementary Figure S1 and Supplementary Table S1). The high-resolution MS results further confirmed that fragment ions at *m/z* 246 and *m/z* 244 were generated *via* eliminations of CH_2_CO and CO_2_, respectively. Thus, the formation of ***p2*** may be interpreted as a result of both rearrangement (hydroxyl transfer or cyclization reaction) and dissociation (elimination of CH_2_ = C=O or CH≡COH) of ***b*** (*m/z* 288).

**SCHEME 2:**
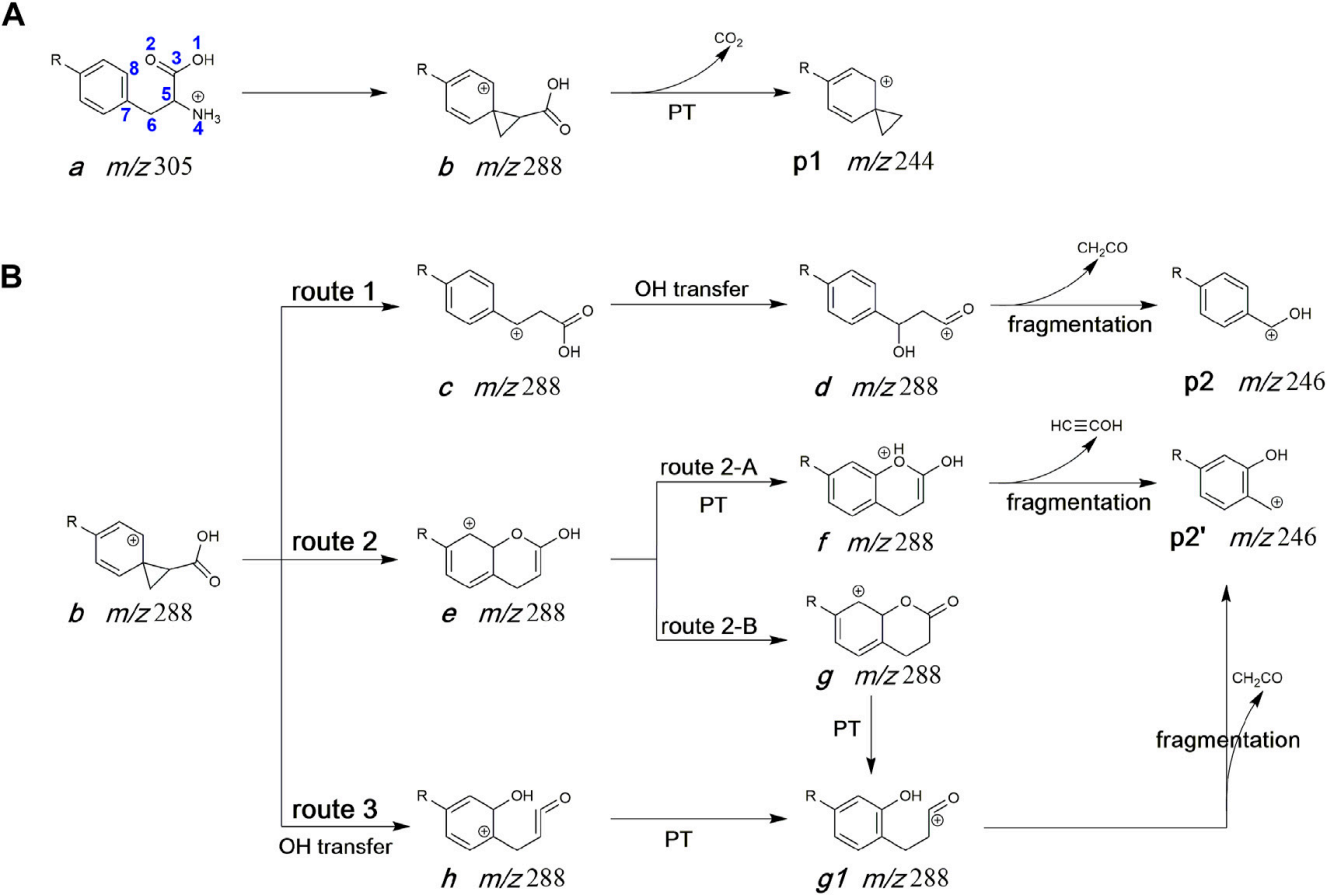
Proposed fragmentation mechanism of protonated melphalan. **(A)** The formation process of m/z 244. **(B)** The formation process of m/z 246. R: N(C2H4Cl)2, PT: proton transfer.

Many literatures have reported that the loss of NH_3_ form protonated phenylalanine is predicted to occur *via* a neighbouring group attack by the aryl group to form a phenonium cation rather than by 1,2-hydride migration from the perspective of experiment or calculation (Shoeib et al., 2002; El Aribi et al., 2004; Lioe and O’Hair, 2005; Lioe and O’Hair, 2007; Lam and O'Hair, 2010). In addition, Liam and O'Hair have fully considered several structures for the [MH-NH_3_]^+^ fragment and showed that the most stable one is not the phenonium cation but that the most favorable NH_3_ loss pathway is however leadint to this structure (Dookeran et al., 1996; Lam and O'Hair, 2010). Thus, the structure of deamination product (ion ***b***, *m/z* 288) was also considered as a ternary ring structure (see Scheme 2) in this work. The potential pathways for the generation of product ions at *m/z* 246 and *m/z* 244 were displayed in Scheme 2. For the generation of *m/z* 244, the hydrogen atom on carboxyl undergoes 1,3-H transfer to form an intermediate isomer, which undergoes the dissociation to form ***p1*** (spiro [2.5]octa-5,7-dien-6-N(C_2_H_4_Cl)_2_-4-ylium) *via* elimination of CO_2_ (Scheme 2A). Three potential routes for the generation of *m/z* 246 (***p2***) from protonated melphalan deamination product ***b***) were proposed in Scheme 2B. In route 1, firstly, under collision activation of structure ***b***, a three-membered ring opening reaction occurred to form intermediate ***c***, then the hydroxyl is transferred from the carbonyl carbon atom (***C*3**) to the interposition carbon atom (***C*6**) of carbonyl leading to intermediate ***d***, followed by the elimination of CH_2_CO to give Ph-CHOH^+^ ion (***p2***). In route 2, the oxygen atom on the carbonyl group first conducts a nucleophilic attack on the positively charged *ortho*-position carbon atom of the phenyl ring group, thereby generating the bicyclic intermediate ***e***. Then, the proton at carbon atom (***C*8**) of intermediate ***e*** undergoes a 1,2-transfer to form intermediate ***f***, followed by dissociation to lose CH≡COH to give HO-Ph-CH_2_^+^ ion (***p2′***, route 2-A). Alternatively, the formed intermediate ***e*** undergoes enol interconversion to form a keto structure ***g***, and the proton at carbon atom (***C*8**) of intermediate ***g*** directly undergoes further 1,2-transfer to form intermediate ***g1***, followed by dissociation to lose CH_2_CO to give ***p2’*** (route 2-B). In route 3, the hydroxyl is transferred from the carbonyl carbon atom (***C*3**) to the ortho carbon atom (***C*8**) of the benzene ring, leading to the intermediate ***h***, which further undergoes a successive proton transfer to form the intermediate ***g1*** and dissociates to form ***p2’***.

### Native ^37^Cl isotope experiments

The postulated fragmentation mechanism in Scheme 2 was supported by the MS/MS analysis on the native ^37^Cl isotopic ion (Figures 1C–F). The chlorine element has two isotopes, ^35^Cl and ^37^Cl in nature, with a relative abundance at 100% and 33%, respectively. Abundant native ^37^Cl isotope protonated molecular ions of melphalan at *m/z* 307 and *m/z* 309 were generated in the positive mode, and ions at *m/z* 305, m*/z* 307, and *m/z* 309 with characteristic abundance ratio of 9:6:1 (Supplementary Figure S1A). As shown in Figure 1C, the product ion ***b*** at *m/z* 290 was observed in the fragmentation spectra of the native ^37^Cl isotopologue of [**1** + H]^+^ at *m/z* 307 (one ^37^Cl atom and one ^35^Cl atom). This indicates that ***b*** contains two Cl atoms. Similarly, as shown in Figure 1D, the mass shift of ***p2*** (from *m/z* 246 to *m/z* 248) and ***p1*** (from *m/z* 244 to *m/z* 246) increase by 2 Da in the MS^3^ spectrum of the protonated ^37^Cl isotopologue (*m/z* 307→*m/z* 290→), which provides evidence that ***p1*** and ***p2*** both contain two Cl atoms. In addition, the product ion ***b*** at *m/z* 292 was observed (Figure 1E) in the fragmentation spectrum of the native ^37^Cl isotope of [**1** + H]^+^ at *m/z* 309 (two ^37^Cl atoms). And the mass shift of ***p2*** (from *m/z* 246 to *m/z* 250) and ***p1*** (from *m/z* 244 to *m/z* 248) increase by 4 Da in the MS^3^ spectrum of the protonated ^37^Cl isotope (*m/z* 309→*m/z* 292→) (Figure 1F, which further proves that ***p1*** and ***p2*** both contain two Cl atoms. These native isotope experiment results further confirm that fragment ion **p1** (*m/z* 244) and **p2** (*m/z* 246) are generated due to the elimination of CO_2_ and CH_2_ = C=O/CH≡COH from ***b***, respectively.

### Density functional theory calculations

To further investigate the mechanism displayed in Scheme 2, density functional theory (DFT) calculations were carried out at the B3LYP/6-31+G (d,*p*) level of theory. There are multiple potential protonation sites for melphalan, including ***O*1** of the hydroxyl, ***O*2** of the carbonyl, ***N*4** of the amino, ***N*9** of the nitrogen mustard, and ***Cl*10** of the nitrogen mustard. The structures with different protonation sites of melphalan were optimized at the same level B3LYP/6-31+G (d,*p*) and the relative energies of these structures are summarized in Table 1. The free energy of the protonation site on the ***N*4** atom is 40.9 kJ/mol, 132.0 kJ/mol, 133.0 kJ/mol, and 95.4 kJ/mol lower than those of protonation sites on the ***N*9** atom, ***O*1** atom, ***O*2** atom, and ***Cl*10** atom, respectively. Overall, the calculation results indicate that the ***N*4** atom is the most thermodynamically favorable protonation site, which gives rise to ion ***a*** (*m/z* 305) as described in Scheme 2. Subsequently, the breakage of the ***C*5**–***N*4** bond in ion ***a*** occurs easily, resulting in the formation of ion ***b*** (*m/z* 288) with a three-membered ring structure and NH_3_. The detailed process was displayed in Supplementary Figure S2. The same mechanism to form a deamination product with a three-membered was also invoked by O’Hair and co-workers (Lioe et al., 2004; Lioe and O’Hair, 2007).

**TABLE 1:** Relative energies of [**1** + H]^+^ with different protonation sites calculated at B3LYP/6-31+G (d,*p*) level.

Compound 1	Site of protonation	Relative energy (kJ mol^−1^)
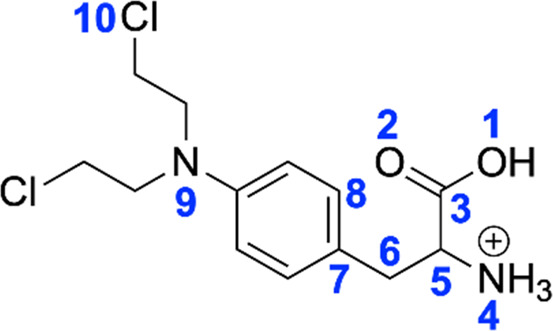	O1 of the hydroxyl	132.0
	*O*2 of the carbonyl	133.0
	*N*4 of the ammonia	0.0
	*N*9 of the Phenylamino	40.9
	*Cl*10 of the chloromethane	95.4

**FIGURE 2:**
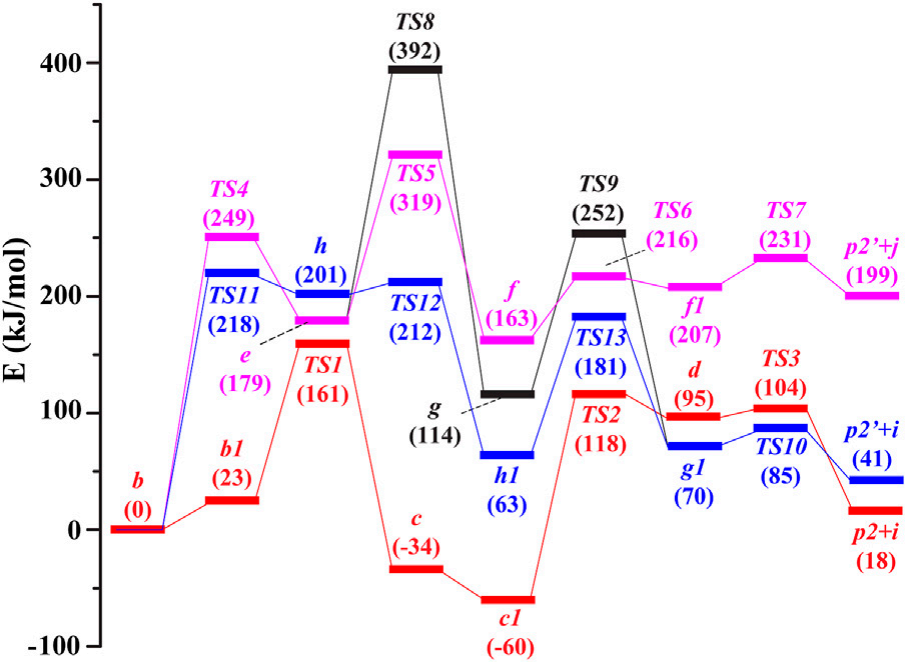
Potential energy diagram for the generation of *m/z 246* in the dissociation of [melphalan + H–NH_3_]^+^ at the B3LYP/6-31+G (d,*p*) level. Red line: route 1, rose red line: route 2-A, black line: route 2-B, blue line: route 3. i: CH_2_CO, j: CH≡COH.

**FIGURE 3:**
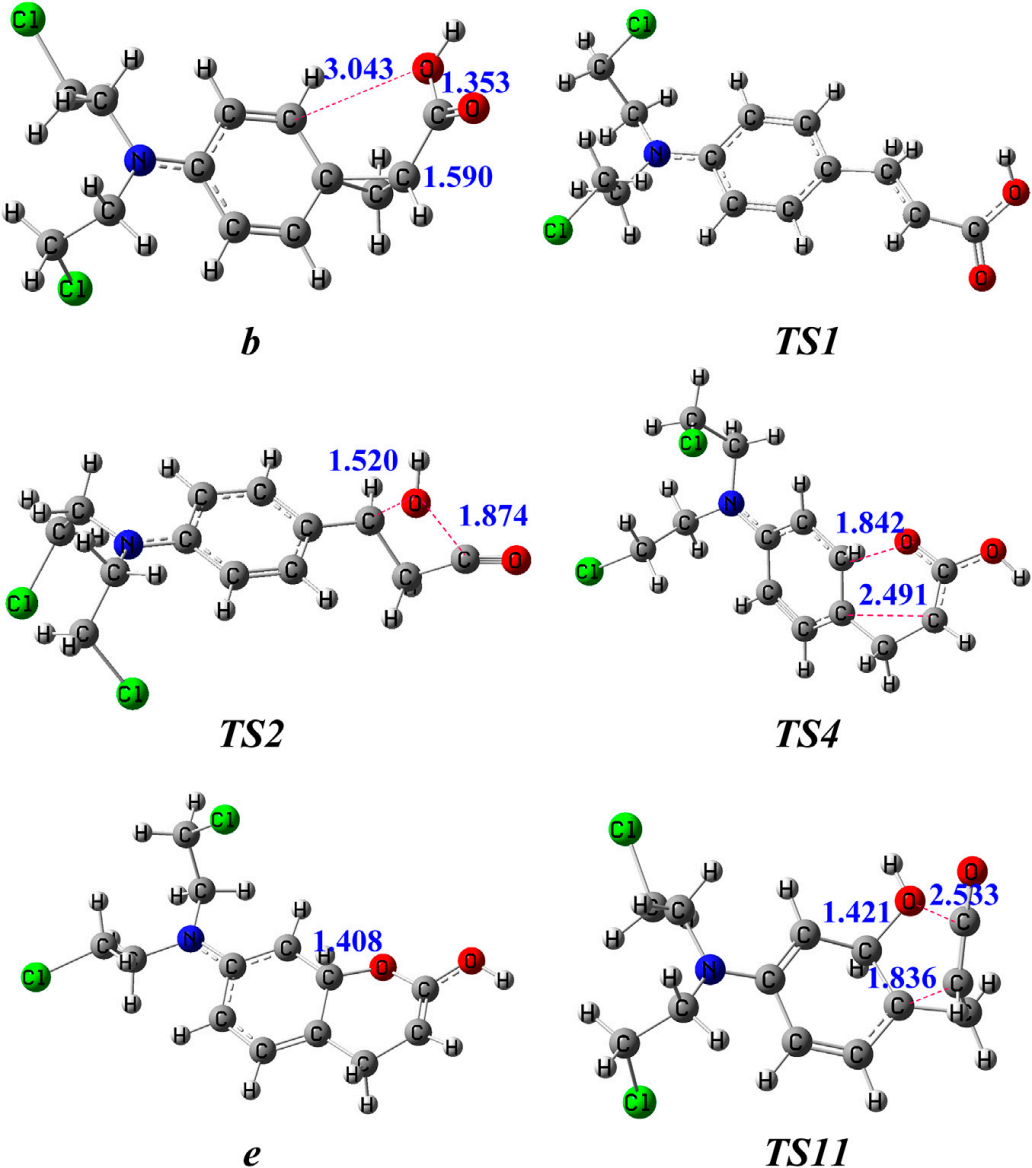
The optimized structures of key intermediates for the generation of *m/z 246* in the dissociation of [melphalan + H–NH_3_]^+^ at the B3LYP/6-31+G (d,*p*) level. The bond lengths are given in Å.

The potential routes to the ion *m/z* 246 in the subsequent fragmentations of ***b*** (*m/z* 288) were compared by theoretical calculations (Figure 2), and details of the corresponding structures are available in the Supplementary Material. In route 1, firstly, under collision activation, structure ***b*** can undergo rotation of -COOH to generate intermediate ***b1*** *via* overcoming a small energy barrier. Then, the three-membered ring in intermediate ***b1*** undergoes an opening reaction to form intermediate ***c*** *via* a transition state (***TS1*** in Figure 3). This process needs to surmount an energy barrier of 161 kJ/mol. The formed intermediate ***c*** continues to undergo rotation of -COOH to generate a more stable intermediate ***c1***. The hydroxyl in ***c1*** is transferred from the carbonyl carbon atom (***C*3**) to the interposition carbon atom (***C*6**) of carbonyl through a four-membered ring transition state (***TS2*** in Figure 3), leading to the formation of ion ***d*** with a new carbon-oxygen bond. This process needs to surmount an energy barrier of 178 kJ/mol. As shown in Figure 3, the distance between the ***C*3** atom and the ***O*1** atom is increased to 1.874 Å in ***TS2***, which is appreciably longer than a covalent ***C*3**–***O*1** bond (1.353 Å) in ***b***. And the distance between the ***C*6** atom and ***O*1** atom is decreased to 1.520 Å in ***TS2***. The reason for the breakage of the ***C*3**–***O*1** bond and the formation of the ***C*6**–***O*1** bond in ***c*** is due to the nucleophilic attack of the hydroxyl on the positively charged carbon atom (***C*6**) *via* the formation of the four-membered ring transition state. The formed ***d*** continues to undergo the cleavage of the ***C*5**–***C*6** bond induced by the positive charge in the ***C*3** atom and gives rise to ***p2*** and CH_2_CO with an energy barrier of 104 kJ mol^−1^ (***TS3***). The sum free energy of the separated ion ***p2*** and CH_2_CO is higher than that of ***b*** by 18 kJ/mol.

In route 2, firstly, the oxygen atom (***O*2**) on the carbonyl group conducts a nucleophilic attack on the positively charged *ortho*-position carbon atom (***C*8**) of the phenyl ring group through a six-membered ring transition state (***TS4*** in Figure 3), leading to the formation of intermediate ion ***e*** with a new carbon-oxygen bond. This process needs to surmount an energy barrier of 249 kJ/mol. The ring expansion rearrangement reaction occurs with a concerted process with cleavage of the ***C*5**–***C*7** bond and formation of the ***C*8**–***O*2** bond, which can be viewed as an electrophilic substitution of the phenyl ring (Figure 3). As shown in Figure 3, the distance between the ***C*5** atom and ***C*7** atom is increased to 2.491 Å in ***TS4***, which is appreciably longer than a covalent ***C*5**–***C*7** bond (1.590 Å) in ***b***. And the distance between the ***C*8** atom and ***O*2** atom is ranged from 4.135 Å in ***b*** to 1.842 Å in ***TS4*** and ranged from 1.842 Å in ***TS4*** to 1.408 Å in ***e***. The reason for the breakage of the ***C*5**–***C*7** bond and the formation of the ***C*8**–***O*2** bond in ***b*** is due to the nucleophilic attack of the carbonyl oxygen atom on the positively charged carbon atom on the phenyl ring group *via* the formation of the six-membered ring transition state. The free energy of ion ***e*** is 179 kJ/mol higher than that of ion ***b***.

Then, the proton at the ortho-position carbon atom (***C*8**) of the phenyl ring in ***e*** undergoes a 1,2-transfer (***TS5***) to the ***O*2** atom to form intermediate ***f***. This process needs to surmount an energy barrier of 319 kJ/mol. The formed ***f*** continues to undergo the cleavage of the ***O*2**–***C*3** bond induced by the positive charge in the ***O*2** atom (***TS6***) and the cleavage of the ***C*5**–***C*6** bond induced by the positive charge in ***C*3** atom (***TS7***), with an energy barrier of 216 kJ/mol (***TS6***) and 231 kJ/mol (***TS7***), respectively, and gives rise to 4-N(C_2_H_4_Cl)_2_-2-hydroxylbenzyl cation (***p2′***) and CH≡COH (route 2-A). The sum free energy of the separated ion ***p2′*** and CH≡COH is higher than that of ***b*** by 199 kJ/mol. Alternatively, the formed intermediate ***e*** undergoes enol interconversion to form a keto structure ***g***, this process needs to surmount an energy barrier of 392 kJ/mol (***TS8***). Then, the formed ***g*** continues to undergo a 1,2-proton transfer (from the ortho-position carbon atom (***C*8**) of the phenyl ring to the oxygen atom (***O*2**) with an energy barrier of 252 kJ/mol (***TS9***) to form intermediate ***g1***. The formed ***g1*** further undergoes the cleavage of the ***C*5**–***C*6** bond induced by the positive charge in the ***C*3** atom and gives rise to 4-N(C_2_H_4_Cl)_2_-2-hydroxylbenzyl cation (***p2’***) and CH_2_CO (route 2-B).

In route 3, firstly, the hydroxyl in ***b*** is transferred from the carbonyl carbon atom (***C*3**) to the ortho carbon atom on benzene (***C*8**) through a five-membered ring transition state (***TS11*** in Figure 3), leading to the formation of ion ***h*** with a new carbon-oxygen bond. This process needs to surmount an energy barrier of 218 kJ/mol. The rearrangement occurs with a concerted process with cleavage of the ***C*3**–***O*1** bond and formation of the ***C*8**–***O*1** bond, which can be viewed as an electrophilic substitution of the phenyl ring (Figure 3). As shown in Figure 3, the distance between the ***C*3** atom and the ***O*1** atom is increased to 2.533 Å in ***TS11***, which is appreciably longer than a covalent ***C*3**–***O*1** bond (1.353 Å) in ***b***. And the distance between the ***C*8** atom and the ***O*1** atom is ranged from 3.043 Å in ***b*** to 1.421 Å in ***TS11***. The reason for the breakage of the ***C*3**–***O*1** bond in ***b*** and the formation of the ***C*8**–***O*1** bond in ***h*** is due to the nucleophilic attack of the hydroxyl on the positively charged carbon atom on the phenyl ring group *via* the formation of the five-membered ring transition state. In ***h***, the hydroxyl was connected to the ***C*8** atom and the three-membered ring was opened (Figure 3). The free energy of ion ***h*** is 201 kJ/mol higher than that of ion ***b***. Then, the formed ***h*** continues to undergo a two-stepwise proton transfer (1,2-proton transfer from the *ortho*-carbon atom (***C*8**) of the phenyl ring to the ipso-carbon atom (***C*7**) of the phenyl ring followed by 1,3-proton transfer to the ortho carbon atom (***C*5**) of carbonyl) to form ***g1***. The formed ***g1*** continues to undergo the cleavage of the ***C*5**–***C*6** bond induced by the positive charge in the ***C*3** atom and gives rise to 4-N(C_2_H_4_Cl)_2_-2-hydroxylbenzyl cation (***p2′***) and CH_2_CO with a small energy barrier of 85 kJ mol^−1^ (***TS10***). The sum free energy of the separated ion ***p2’*** and CH_2_CO is higher than that of ***b*** by 41.0 kJ/mol.

From the calculation results in Figure 2 for the formation of product ion *m/z* 246, it can be found that the sum free energy of the separated ***p2′*** and CH_2_CO is 158 kJ/mol lower than that of the separated ***p2′*** and CH≡COH, indicating that generation of (***p2’*** + CH_2_CO) is easier than that of (***p2’*** + CH≡COH) from a thermodynamic point of view. The maximum energy barrier in route 2-B (black line) for the formation of (***p2’*** + CH_2_CO) is 174 kJ/mol higher than that in route 3 (blue line) for the formation of (***p2’*** + CH_2_CO), indicating route 3 is easier to occur. Moreover, the maximum energy barrier in route 3 (blue line) for the formation of (***p2’*** +CH_2_CO) is 57 kJ/mol higher than that in route 1 (red line) for the formation of (***p2*** + CH_2_CO), indicating route 1 is the most favorable. Thus, DFT calculations suggested that route 1 was more favorable than route 2 and route 3 from a thermodynamic point of view. That is to say, the collisional activation of protonated phenylalanine derivatives deamination products was more favorable to form ***p2*** (*m/z* 246) *via* hydroxyl transfer rather than cyclization reaction. The same hydroxyl transfer mechanism was also invoked by El Aribi et al. (El Aribi et al., 2004).

For the elimination of the CO_2_ route (Figure S3), the H atom on the carboxyl of ***b*** directly undergoes 1,3-H transfer to the *ortho* carbon atom of carbonyl ***C*5** *via* a four-membered ring transition state (***TS14***), which was further separated into spiro product ***p1*** (spiro [2.5]octa-5,7-dien-6-N(C_2_H_4_Cl)_2_-4-ylium) and CO_2_. This process needs to surmount the energy barrier of 288 kJ/mol. The sum free energy of the separated ***p1*** and CO_2_ is 114 kJ/mol lower than that of the separated ***p2*** and CH_2_CO, indicating that ***p1*** is a more stable structure. However, the maximum energy barrier in the route for the formation of (***p1*** + CO_2_) is 127 kJ/mol higher than that in route 1 (red line) for the formation of (***p2*** + CH_2_CO). Thus, DFT calculations suggested that route 1 was more favorable than the route for the elimination of CO_2_ from a thermodynamic point of view.

### The universality of the gas-phase hydroxyl transfer

**FIGURE 4:**
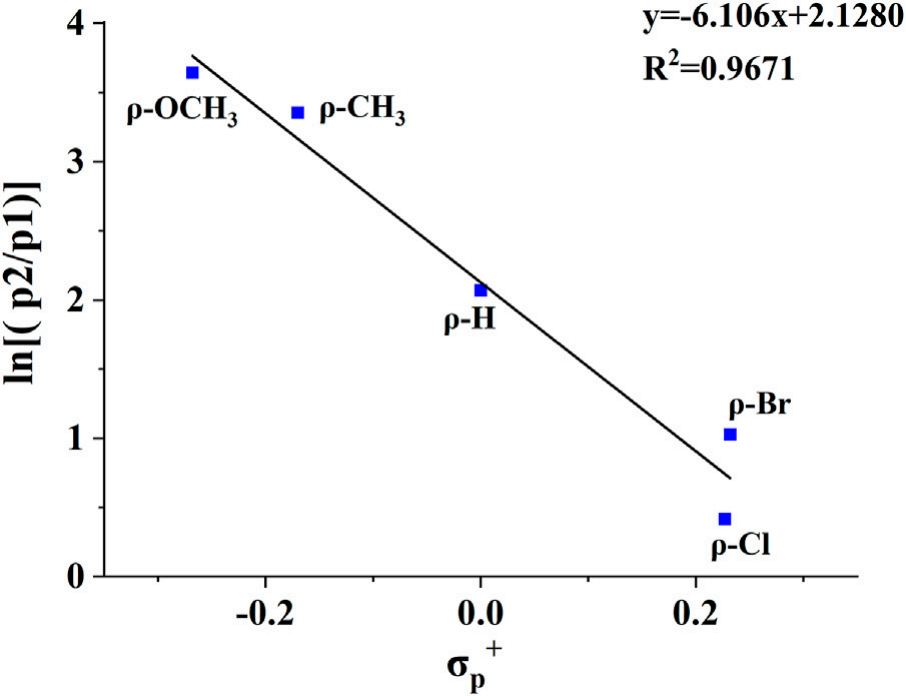
The plot of ln [(***p2***)/(***p1***)] vs the σ_p_^+^ substituent constants for the CID reactions of compounds 2, 4, 5, 6, 8.

Substituent effects are very useful in probing reaction mechanisms (Guo et al., 2012; Wang et al., 2015; Zhang et al., 2015; Wang et al., 2016). To better delineate the universality of the gas-phase hydroxyl transfer reaction, a series of compounds bearing different substituents (H-substituted, OCH_3_-substituted, Cl-substituted, CH_3_-substituted, OH-substituted, Br-substituted) at the *para* position of the phenyl ring were also investigated by tandem MS experiments (Supplementary Figure S4-10), and the tandem MS data were summarized in Table 2. Noteworthily, corresponding fragment ions of ***p2*** (*m/z* 107, m*/z* 167, m*/z* 141, m*/z* 137, m*/z* 121, m*/z* 123 and *m/z* 185 for compounds **2**-**8**) and fragment ions of ***p1*** (*m/z* 105, m*/z* 165, m*/z* 139, m*/z* 135, m*/z* 119, m*/z* 121 and *m/z* 183 for compounds **2**-**8**) were observed for all compounds. All of these compounds show similar fragmentation behaviors in the CID-MS experiments, whereas the relative intensities of the two competing product ions varied as the substituent changed (Table 2). Mainly, the presence of the electron-donating groups on the phenyl ring favors both the H transfer to give ***p1*** and the hydroxyl transfer to give ***p2***, whereas the presence of the electron-withdrawing groups suppresses both ***p1*** and ***p2***. The reason was that the electron of the electron-donating group is transferred to the positive charge site on ***p2***, which effectively reduces the positivity of the carbon atom of ***p2***, thus increasing its stability. A plot of the abundance ratio of these two ions, ln [(***p2***)/(***p1***)] *versus* the Hammett substituent constants (Hansch et al., 1991), *σ*_p_^+^, was obtained in Figure 4. The logarithmic values of the abundance ratios of these two ions are in line with *σ*_p_^+^.

**TABLE 2:** The CID mass spectra data of [M + H–NH_3_]^+^ from protonated phenylalanine derivatives (1–8).

Compounds	[M + H]^+^ *m/z*	[M + H–NH_3_]^+^ *m/z* (%)	Loss of 42 Da *m/z* (%)	Loss of 44 Da *m/z* (%)	Other ions *m/z* (%)
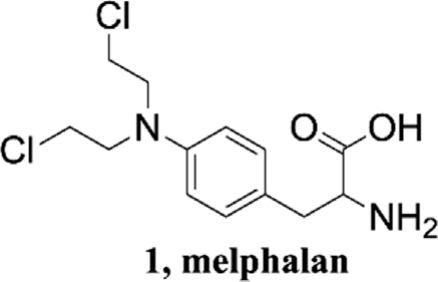	305	288 (11)	246 (100)	244 (11)	2422), 2702)
	307	2903)	248 (100)	246 (11)	2442), 2722)
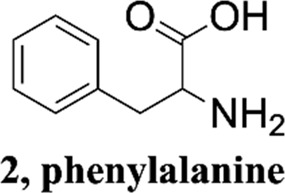	166	1493)	1073)	1051)	1213), 1321)
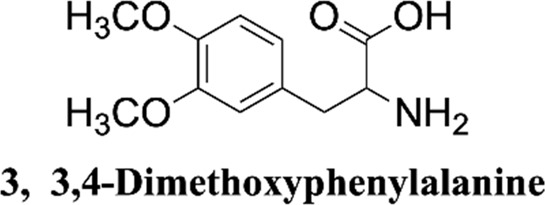	226	2095)	167 (68)	1653)	163(68), 1901), 1915), 2095)
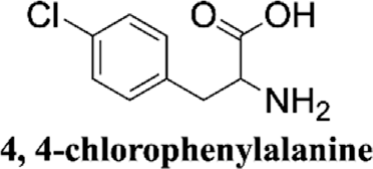	200	1832)	1414)	1391)	165 (100)
	202	185 (62)	1433)	141 (0.29)	167(100), 1574)
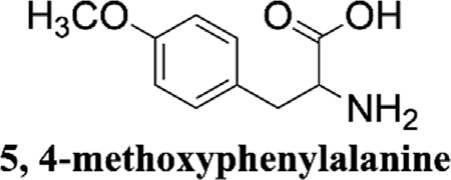	196	1796)	137 (100)	1353)	133(88), 161(54)
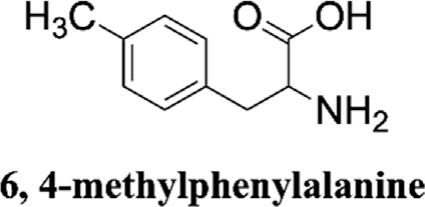	180	1633)	121 (18)	1191)	145 (100)
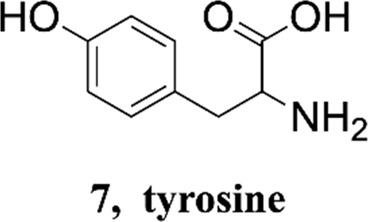	182	1652)	123 (25)	1211)	147(100), 119(27)
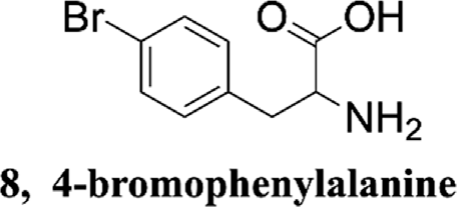	244	2273)	1854)	1831)	209 (100)
	246	2293)	1874)	1851)	211 (100)

## Conclusion

In summary, an intriguing intramolecular hydroxyl transfer reaction occurred in the gas-phase dissociation of protonated phenylalanine derivatives. Accurate mass measurements, native isotope experiments, multiple-stage mass spectrometry experiments, different substituents experiments, and DFT calculations indicate convincing evidence that the hydroxyl transfer was achieved to form hydroxylbenzyl cation *via* eliminations of NH_3_ and CH_2_CO. This study reports hydroxyl transfer reaction in the gas phase chemistry, which can not only aid us to elucidate the structural information of compounds but also help to discover new pathways of drug metabolism.

## Data Availability

The original contributions presented in the study are included in the article/Supplementary Material, further inquiries can be directed to the corresponding authors.
