# Supplementary material for: Hydroxyl transfer versus cyclization reaction in the gas phase: Sequential loss of NH3 and CH2CO from protonated phenylalanine derivatives
Source: Front Chem. 2023 Jan 9;10:1094329. doi: 10.3389/fchem.2022.1094329 (PMC9868239; doi:10.3389/fchem.2022.1094329)
Supplement: Supplementary file 1 [file DataSheet1.DOCX]

Supplementary Material

**Supplementary Table S1.** Accurate masses of product ions in the CID-MS of [**1** + H]^+^, measured by Orbitrap-XL mass spectrometer.

| Measured mass  (*m/z*) | Elemental  composition | Calculated mass  (*m/z*) | Relative error  (ppm) |
| --- | --- | --- | --- |
| 305.0819 | C_13_H_19_Cl_2_N_2_O_2_^+^ | 305.0818 | 0.30 |
| 307.0790 | C_13_H_19_Cl^37^ClN_2_O_2_^+^ | 307.0789 | 0.30 |
| 288.0551 | C_13_H_16_Cl_2_NO_2_^+^ | 288.0553 | -0.69 |
| 290.0521 | C_13_H_16_Cl^37^ClNO_2_^+^ | 290.0523 | -0.69 |
| 246.0443 | C_11_H_14_Cl_2_NO^+^ | 246.0447 | -0.16 |
| 244.0652 | C_12_H_16_Cl_2_N^+^ | 244.0654 | -0.81 |

**
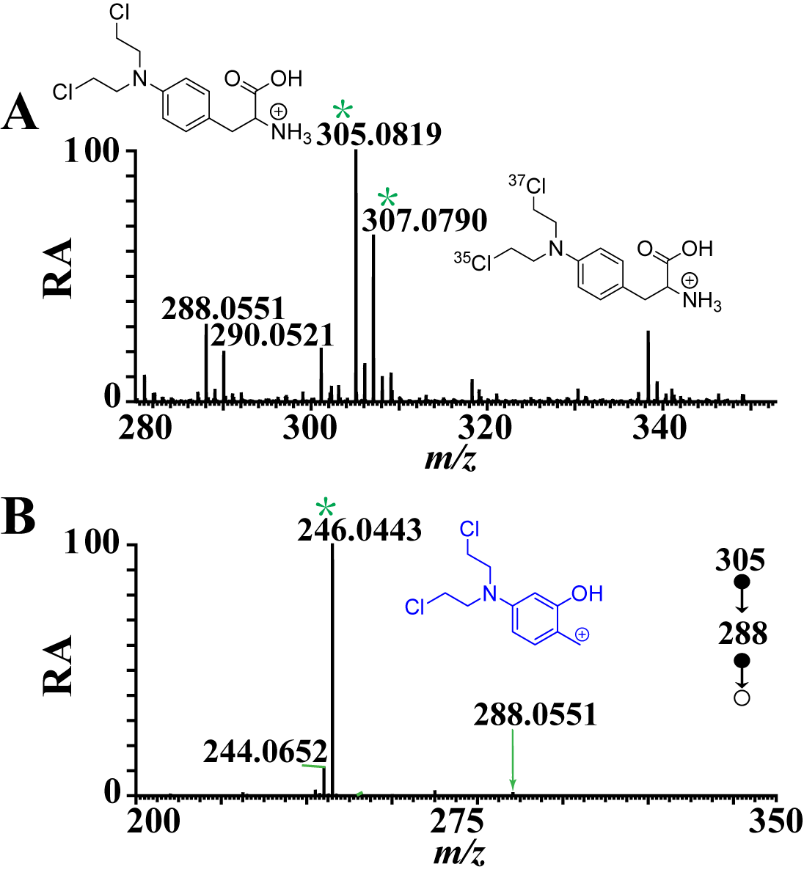
**

**Supplementary Figure S1. High resolution collision-induced dissociation mass spectra of [1 + H]^+^.** (A) MS spectrum of [**1** + H]^+^, (B) MS^3^ spectrum of [**1** + H]^+^ (*m/z* 305.0819 →*m/z* 288.0551 →),

**
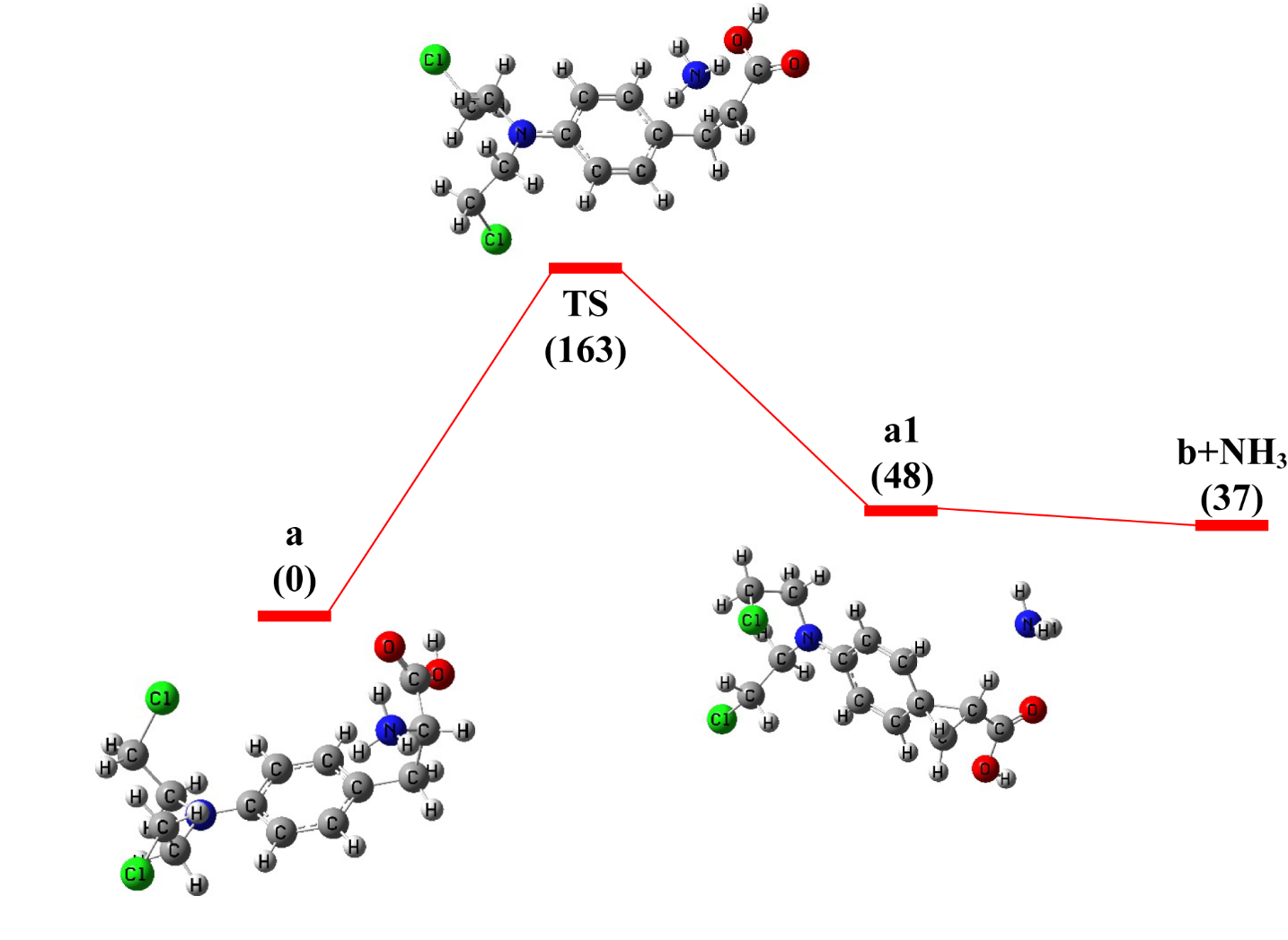
**

**Supplementary Figure S2.** Potential energy diagram for the generation of three-membered ring intermediate *m/z* 288 (***b***) in the dissociation of [**1** + H]^+^ calculated at B3LYP/6-31+G(d,p) level


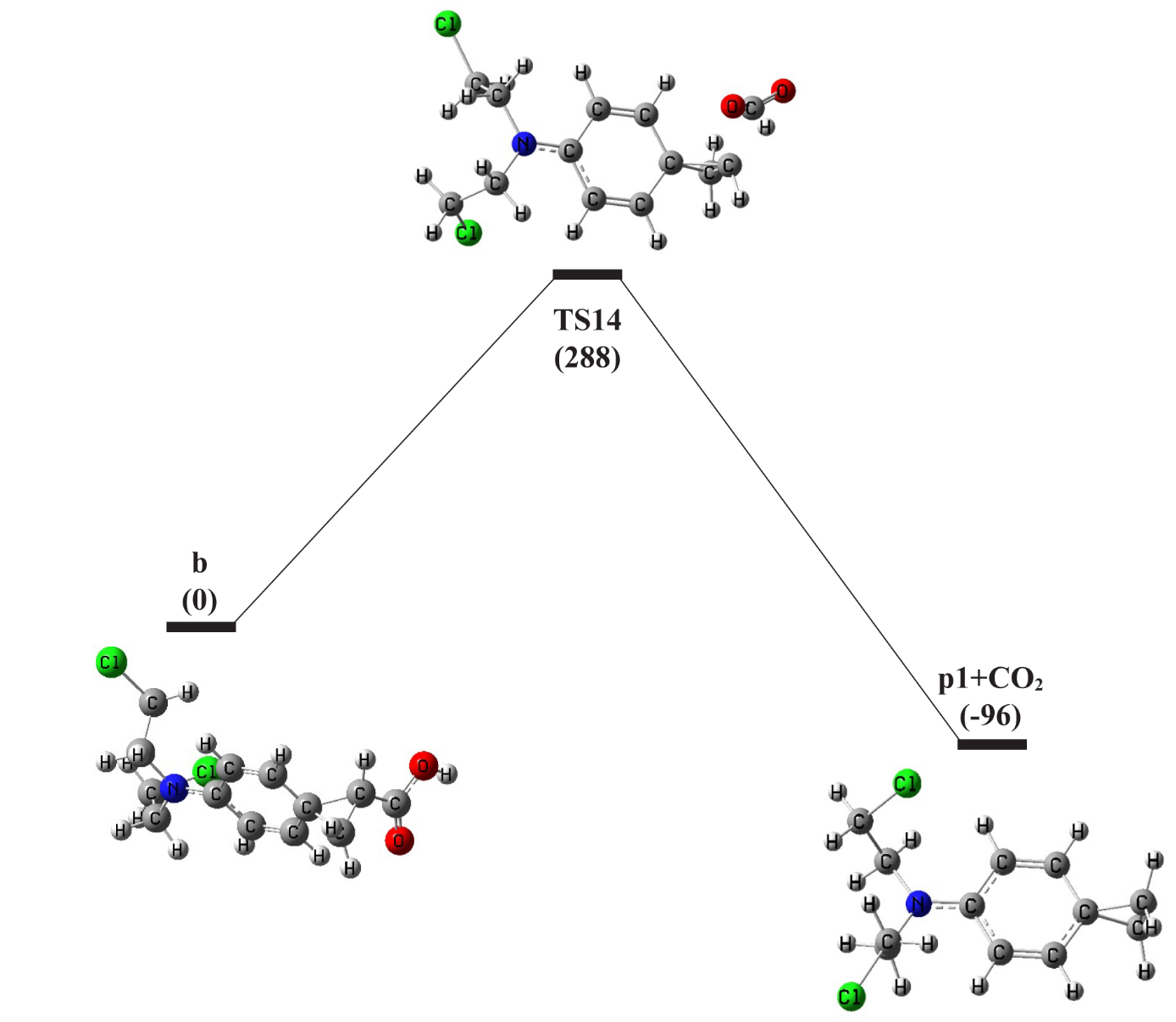


**Supplementary Figure S3.** Potential energy diagram for the generation of spiro product m/z 244 (***p1***) in the dissociation of [1 + H]^+^ calculated at B3LYP/6-31+G(d,p) level.

**
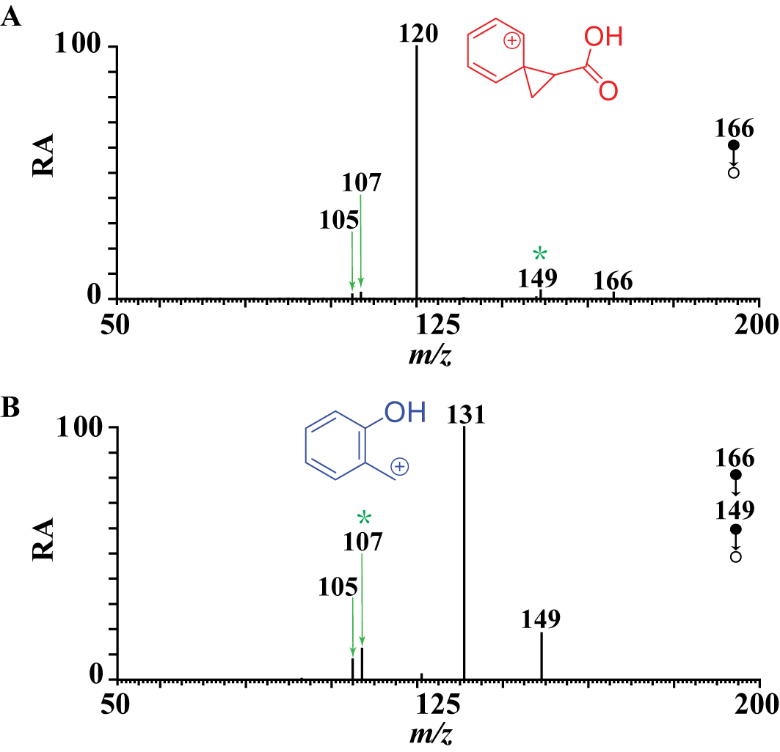
**

**Supplementary Figure S4. Collision-induced dissociation mass spectra of [2 + H]^+^.** (A) MS^2^ spectrum of [**2** + H ]^+^ (*m/z* 166→)，(B) MS^3^ spectrum of [**2** + H]^+^ (*m/z* 166→*m/z* 149→).

**
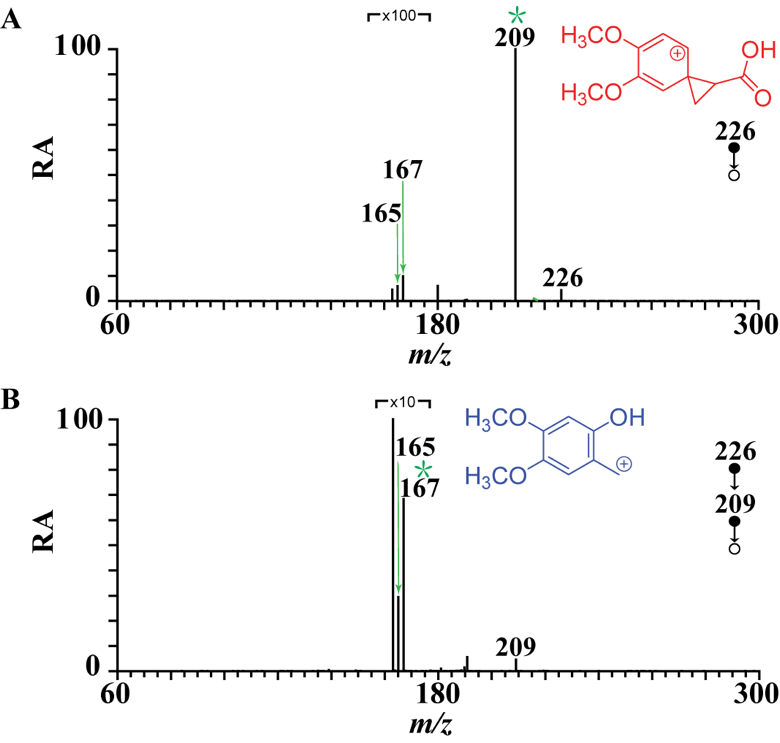
**

**Supplementary Figure S5. Collision-induced dissociation mass spectra of [3 + H]^+^.** (A) MS^2^ spectrum of [**3** + H]^+^ (*m/z* 226→), (B) MS^3^ spectrum of [**3** + H]^+^ (*m/z* 226→*m/z* 209→).

**
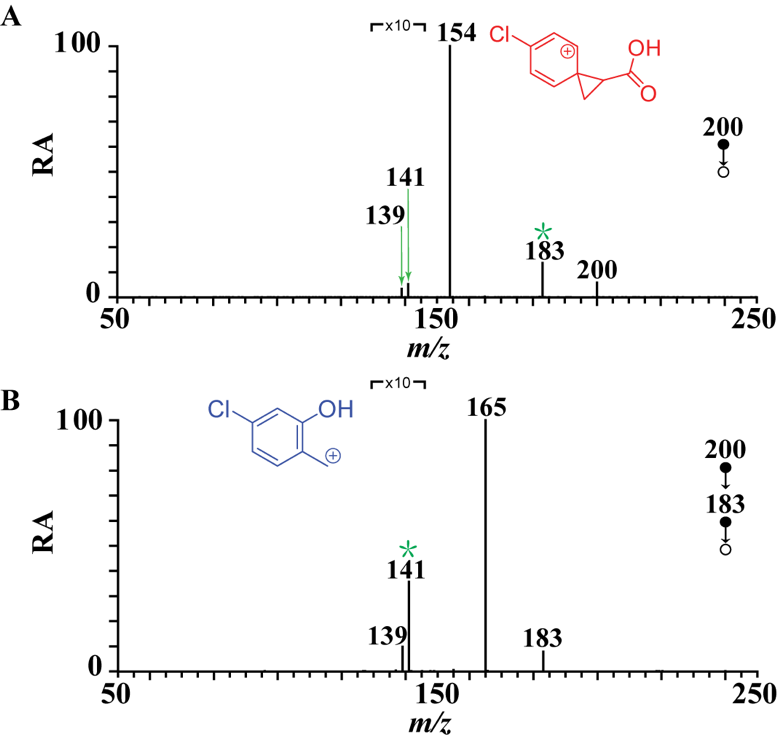
**

**Supplementary Figure S6. Collision-induced dissociation mass spectra of [4 + H]^+^.** (A) MS^2^ spectrum of [**4** + H]^+^ (*m/z* 200→), (B) MS^3^ spectrum of [**4** + H ]^+^ (*m/z* 200→*m/z* 183→).

**
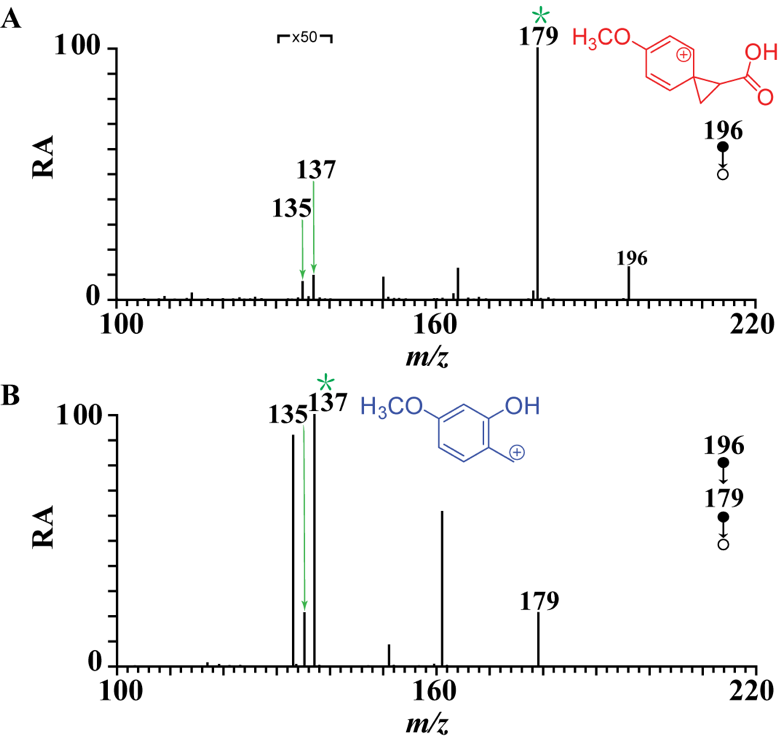
**

**Supplementary Figure S7. Collision-induced dissociation mass spectra of [5 + H]^+^.** (A) MS^2^ spectrum of [**5** + H ]^+^ (*m/z* 196→)，(B) MS^3^ spectrum of [**5** + H]^+^ (*m/z* 196→*m/z* 179→).

**
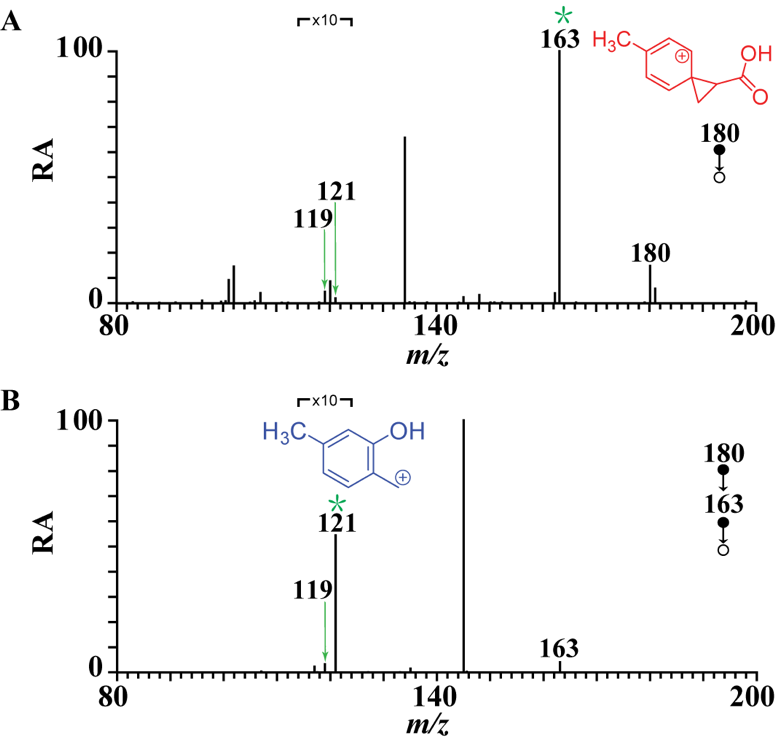
**

**Supplementary Figure S8. Collision-induced dissociation mass spectra of [6 + H]^+^.** (A) MS^2^ spectrum of [**6** + H ]^+^ (*m/z* 180→)，(B) MS^3^ spectrum of [**6** + H]^+^ (*m/z* 180→*m/z* 163→).

**
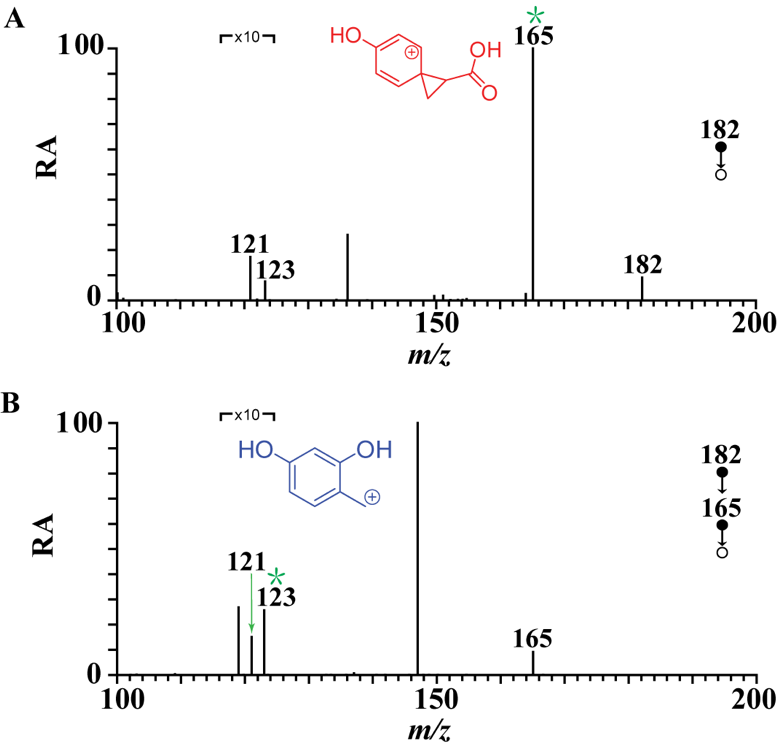
**

**Supplementary Figure S9. Collision-induced dissociation mass spectra of [7 + H]^+^.** (A) MS^2^ spectrum of [**7** + H ]^+^ (*m/z* 182→)，(B) MS^3^ spectrum of [**7** + H]^+^ (*m/z* 182→*m/z* 165→).

**
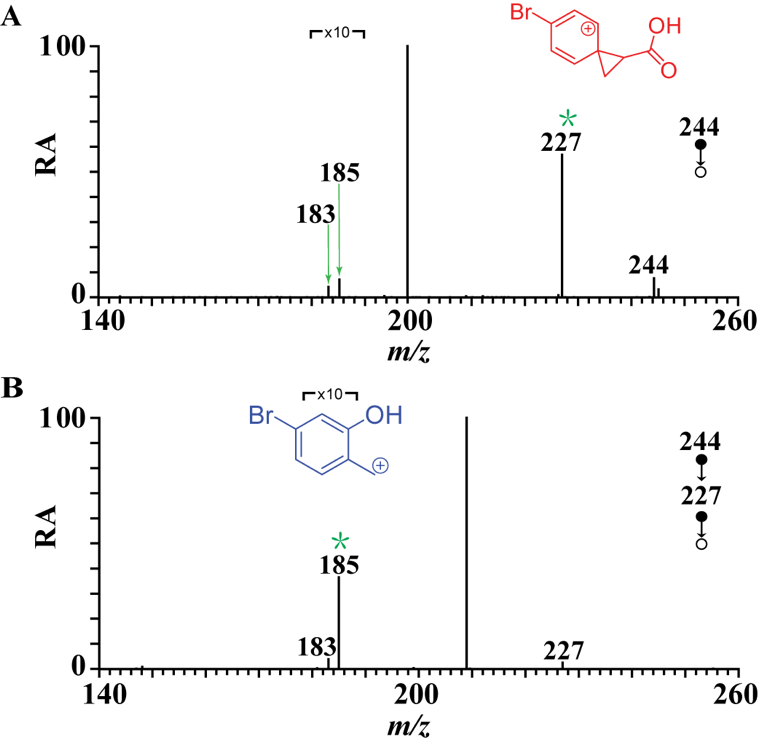
**

**Supplementary Figure S10. Collision-induced dissociation mass spectra of [8 + H]^+^.** (A) MS^2^ spectrum of [**8** + H ]^+^ (*m/z* 244→)，(B) MS^3^ spectrum of [**8** + H]^+^ (*m/z* 244→*m/z* 227→).

**a(compound 1)**


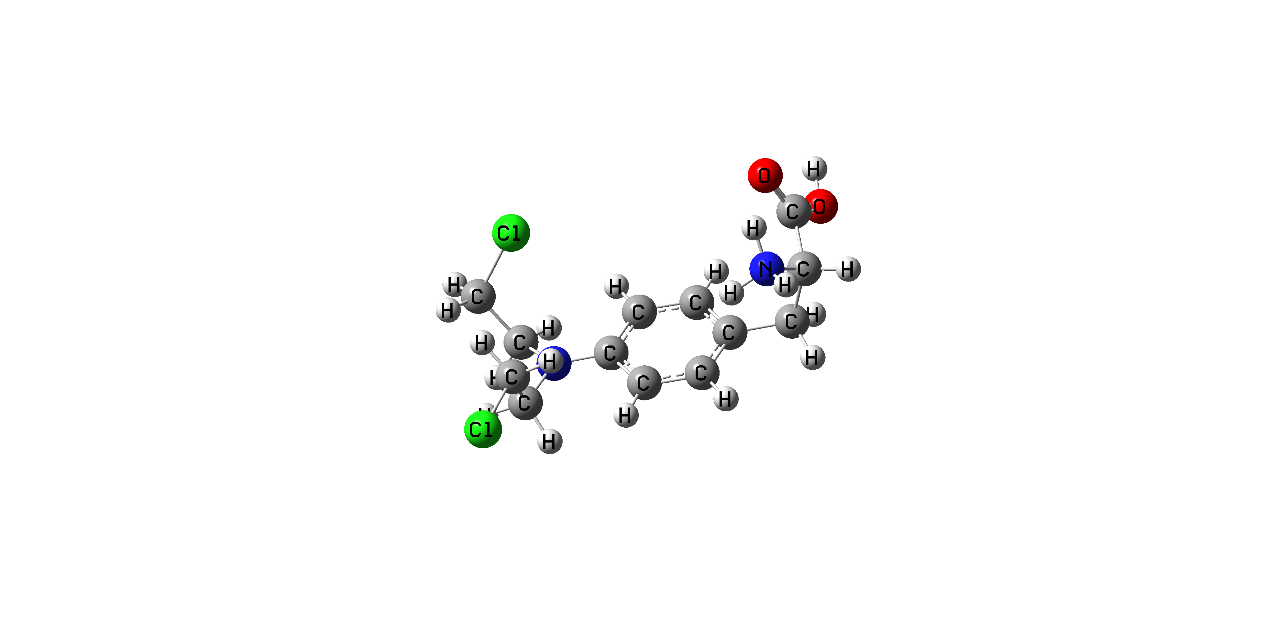


Zero-point correction= 0.316890 (Hartree/Particle)

Thermal correction to Energy= 0.336593

Thermal correction to Enthalpy= 0.337537

Thermal correction to Gibbs Free Energy= 0.264737

Sum of electronic and zero-point Energies= -1686.692559

Sum of electronic and thermal Energies= -1686.672856

Sum of electronic and thermal Enthalpies= -1686.671912

Sum of electronic and thermal Free Energies= -1686.744711

---------------------------------------------------------------------

Center Atomic Atomic Coordinates (Angstroms)

Number Number Type X Y Z

---------------------------------------------------------------------

1 6 0 -0.228269 0.717434 -1.433648

2 6 0 0.395646 -0.257721 -0.636502

3 6 0 -0.413884 -1.271118 -0.081928

4 6 0 -1.784919 -1.302069 -0.302495

5 6 0 -2.416164 -0.312825 -1.077782

6 6 0 -1.610854 0.688540 -1.642709

7 1 0 0.356130 1.491407 -1.916296

8 1 0 0.051166 -2.025990 0.542284

9 1 0 -2.371687 -2.102535 0.139321

10 1 0 -2.050546 1.433881 -2.304413

11 6 0 -3.913548 -0.312253 -1.285688

12 6 0 -4.695631 0.436896 -0.174644

13 1 0 -4.320854 -1.325583 -1.310155

14 1 0 -4.171642 0.150801 -2.244296

15 1 0 -5.759768 0.471586 -0.416603

16 6 0 -4.498298 -0.144205 1.220406

17 8 0 -3.846023 0.444563 2.052626

18 8 0 -5.116675 -1.308287 1.369163

19 1 0 -4.962330 -1.647210 2.270823

20 7 0 -4.180734 1.856079 -0.058401

21 1 0 -4.391316 2.404424 -0.898111

22 1 0 -4.563159 2.335574 0.764810

23 7 0 1.782790 -0.286276 -0.355225

24 6 0 2.455601 -1.360661 -1.128061

25 6 0 3.900972 -1.650791 -0.742782

26 1 0 2.461918 -1.104199 -2.202677

27 1 0 1.882994 -2.280192 -1.010697

28 1 0 4.278646 -2.446570 -1.383343

29 1 0 4.557440 -0.788468 -0.844880

30 6 0 2.486420 1.007457 -0.463867

31 6 0 3.406290 1.211666 0.745947

32 1 0 1.770483 1.826746 -0.470465

33 1 0 3.053370 1.076311 -1.401187

34 1 0 2.834647 1.165429 1.671530

35 1 0 4.201718 0.473943 0.802889

36 17 0 4.187288 2.844296 0.666286

37 17 0 4.064683 -2.238711 0.962925

38 1 0 -3.153706 1.814770 0.058452

--------------------------------------------------------------------

**TS**


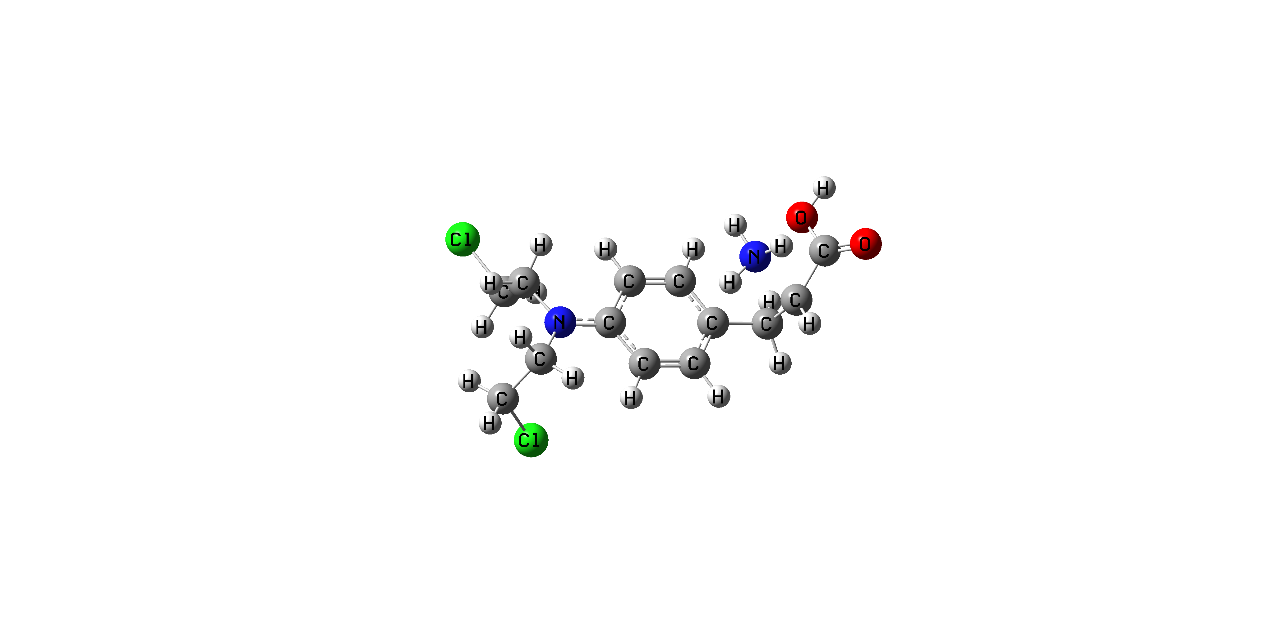


Zero-point correction= 0.310730 (Hartree/Particle)

Thermal correction to Energy= 0.331717

Thermal correction to Enthalpy= 0.332661

Thermal correction to Gibbs Free Energy= 0.256929

Sum of electronic and zero-point Energies= -1686.628725

Sum of electronic and thermal Energies= -1686.607737

Sum of electronic and thermal Enthalpies= -1686.606793

Sum of electronic and thermal Free Energies= -1686.682526

---------------------------------------------------------------------

Center Atomic Atomic Coordinates (Angstroms)

Number Number Type X Y Z

---------------------------------------------------------------------

1 6 0 -0.515961 -1.608868 -0.164268

2 6 0 -0.765143 -0.250487 -0.521668

3 6 0 0.336355 0.654007 -0.429028

4 6 0 1.593291 0.218173 -0.058375

5 6 0 1.835135 -1.127704 0.274589

6 6 0 0.746249 -2.024424 0.209090

7 1 0 -1.309476 -2.343328 -0.189396

8 1 0 0.188155 1.712961 -0.587686

9 1 0 2.392101 0.946713 0.020635

10 1 0 0.894485 -3.071585 0.459879

11 6 0 3.166701 -1.654929 0.703578

12 6 0 4.433768 -0.984516 0.482992

13 1 0 3.369447 -2.546613 0.047999

14 1 0 3.126246 -2.092314 1.708354

15 1 0 5.248875 -1.191061 1.169106

16 6 0 4.857351 -0.194863 -0.696785

17 8 0 5.831752 0.531057 -0.667209

18 8 0 4.096211 -0.409274 -1.787211

19 1 0 4.489519 0.074178 -2.535787

20 7 0 4.375376 1.359175 2.212140

21 1 0 4.688170 1.110517 3.146885

22 1 0 5.131250 1.845766 1.736068

23 7 0 -2.013319 0.161717 -0.914224

24 6 0 -2.261261 1.468575 -1.531122

25 6 0 -3.060022 2.454556 -0.679178

26 1 0 -2.837725 1.297833 -2.450435

27 1 0 -1.316959 1.914563 -1.838508

28 1 0 -3.219600 3.373755 -1.245812

29 1 0 -4.031463 2.053651 -0.386353

30 6 0 -3.174701 -0.734787 -0.821462

31 6 0 -3.684677 -0.890918 0.618095

32 1 0 -2.936369 -1.714284 -1.246084

33 1 0 -3.970010 -0.316674 -1.441425

34 1 0 -2.916604 -1.287822 1.282407

35 1 0 -4.043209 0.055395 1.023896

36 17 0 -5.066055 -2.050598 0.639038

37 17 0 -2.200251 2.911157 0.846735

38 1 0 3.579753 1.985457 2.299310

---------------------------------------------------------------------

**a1**


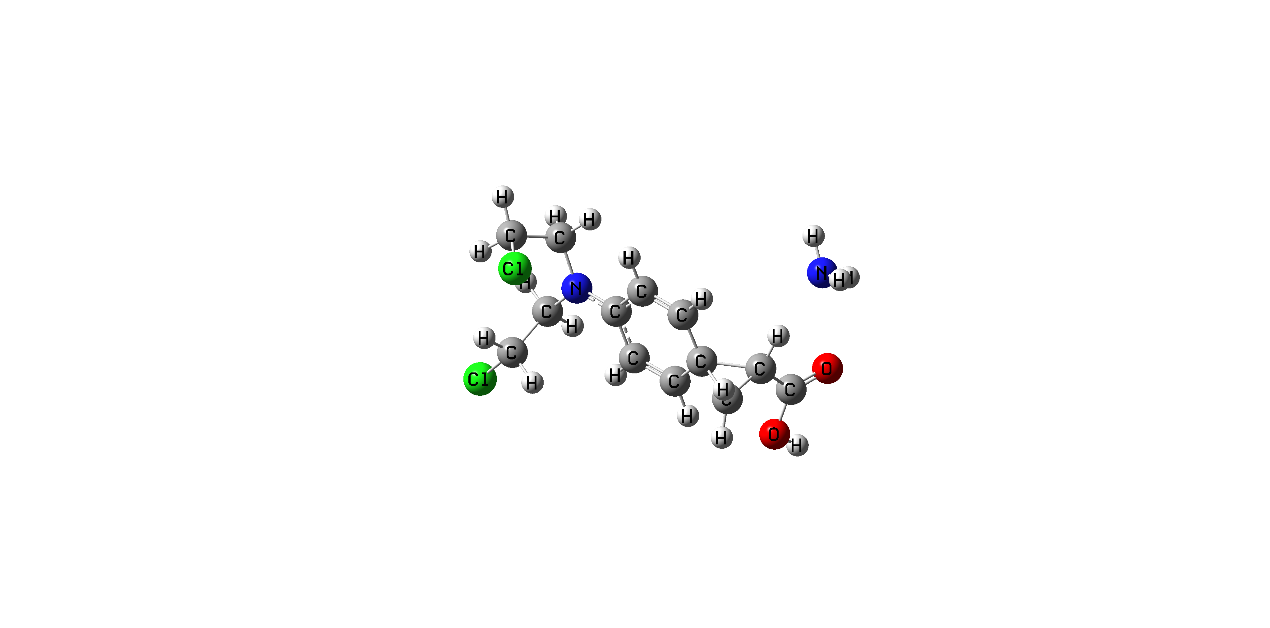


Zero-point correction= 0.311693 (Hartree/Particle)

Thermal correction to Energy= 0.333641

Thermal correction to Enthalpy= 0.334585

Thermal correction to Gibbs Free Energy= 0.255451

Sum of electronic and zero-point Energies= -1686.669928

Sum of electronic and thermal Energies= -1686.647981

Sum of electronic and thermal Enthalpies= -1686.647037

Sum of electronic and thermal Free Energies= -1686.726170

---------------------------------------------------------------------

Center Atomic Atomic Coordinates (Angstroms)

Number Number Type X Y Z

---------------------------------------------------------------------

1 6 0 0.077803 0.870107 -0.071619

2 6 0 -0.680668 -0.284238 0.306862

3 6 0 -0.049487 -1.554459 0.107782

4 6 0 1.222496 -1.650428 -0.417053

5 6 0 1.952965 -0.501758 -0.816969

6 6 0 1.354581 0.760832 -0.587140

7 1 0 -0.341889 1.861716 0.033265

8 1 0 -0.590534 -2.470802 0.298594

9 1 0 1.637968 -2.639837 -0.589050

10 1 0 1.891067 1.665409 -0.857582

11 6 0 3.270756 -0.634444 -1.590717

12 6 0 4.103979 -0.639080 -0.387157

13 1 0 3.448514 0.215698 -2.248211

14 1 0 3.309622 -1.571905 -2.150652

15 1 0 4.224802 -1.552611 0.179748

16 6 0 5.107164 0.413855 -0.056220

17 8 0 6.178059 0.119483 0.431229

18 8 0 4.762695 1.648133 -0.461984

19 1 0 5.513929 2.247424 -0.301384

20 7 0 3.366601 -0.021703 2.058322

21 1 0 2.582714 -0.635959 2.264146

22 1 0 4.088849 -0.184225 2.757711

23 7 0 -1.940409 -0.166585 0.827937

24 6 0 -2.661836 -1.288921 1.446175

25 6 0 -3.869279 -1.805519 0.664324

26 1 0 -3.029911 -0.946052 2.422169

27 1 0 -1.969207 -2.104476 1.646730

28 1 0 -4.345530 -2.608491 1.230065

29 1 0 -4.608911 -1.024456 0.482143

30 6 0 -2.630791 1.134640 0.876368

31 6 0 -3.179542 1.566318 -0.490754

32 1 0 -1.964457 1.904331 1.275780

33 1 0 -3.456659 1.046462 1.584696

34 1 0 -2.393985 1.633781 -1.243968

35 1 0 -3.950210 0.884610 -0.851072

36 17 0 -3.925767 3.202483 -0.344178

37 17 0 -3.410017 -2.485014 -0.947016

38 1 0 3.043944 0.938063 2.156112

---------------------------------------------------------------------

**b**


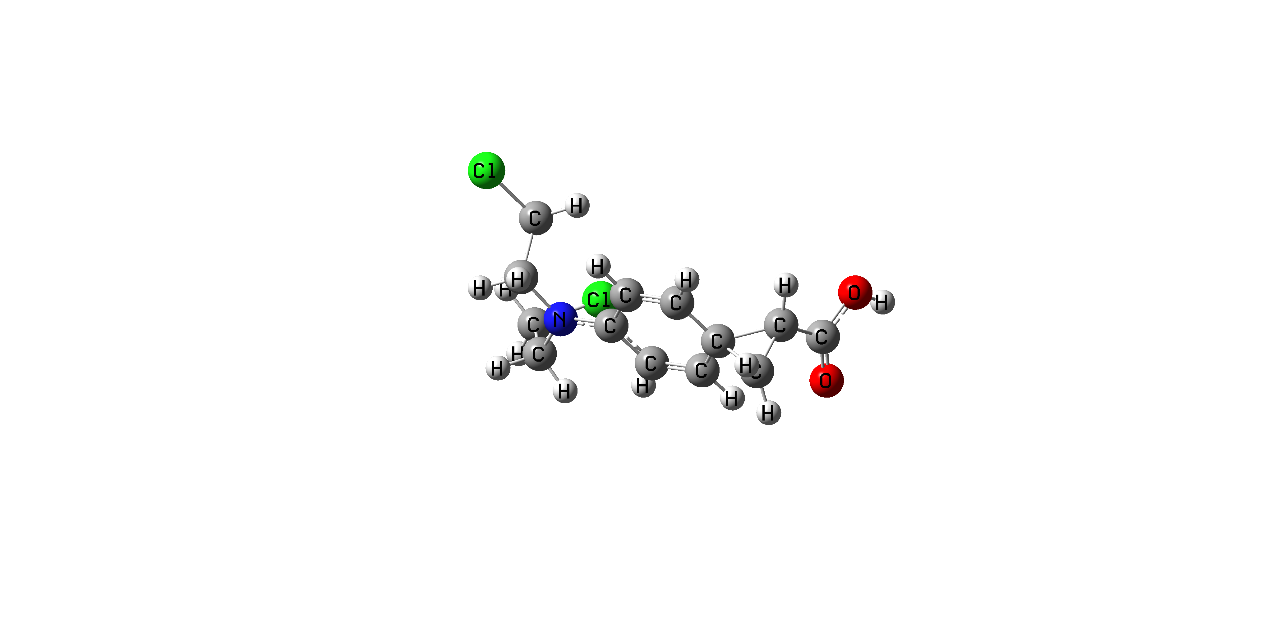


Zero-point correction= 0.275149 (Hartree/Particle)

Thermal correction to Energy= 0.292975

Thermal correction to Enthalpy= 0.293919

Thermal correction to Gibbs Free Energy= 0.226209

Sum of electronic and zero-point Energies= -1630.126019

Sum of electronic and thermal Energies= -1630.108192

Sum of electronic and thermal Enthalpies= -1630.107248

Sum of electronic and thermal Free Energies= -1630.174958

---------------------------------------------------------------------

Center Atomic Atomic Coordinates (Angstroms)

Number Number Type X Y Z

---------------------------------------------------------------------

1 6 0 0.396164 -1.684628 -0.174751

2 6 0 -0.084994 -0.310878 0.103062

3 6 0 0.703986 0.741998 -0.289425

4 6 0 2.000077 0.516006 -0.997011

5 6 0 2.630211 -0.835923 -0.679184

6 6 0 1.662302 -1.945015 -0.540129

7 1 0 0.340651 1.763322 -0.278930

8 1 0 1.933186 0.644744 -2.088067

9 1 0 1.991500 -2.959192 -0.743876

10 6 0 4.049277 -1.030132 -1.038429

11 6 0 3.746542 -0.690185 0.431930

12 1 0 4.558837 -0.273418 -1.626077

13 1 0 4.412705 -2.044374 -1.175310

14 1 0 3.866912 -1.447259 1.201092

15 6 0 3.924859 0.634343 0.902463

16 8 0 3.038100 1.477809 -0.577902

17 8 0 4.297894 1.393487 1.686972

18 1 0 2.649422 2.323621 -0.293448

19 7 0 -1.337043 -0.167356 0.662780

20 6 0 -1.956651 -1.213581 1.486560

21 6 0 -3.280389 -1.754040 0.953242

22 1 0 -2.154996 -0.794290 2.483294

23 1 0 -1.254833 -2.033413 1.639373

24 1 0 -3.707297 -2.454928 1.672867

25 1 0 -4.004891 -0.959668 0.766489

26 6 0 -1.931251 1.168556 0.765867

27 6 0 -2.539495 1.632110 -0.563193

28 1 0 -1.187215 1.893357 1.118140

29 1 0 -2.713351 1.140982 1.527446

30 1 0 -1.823768 1.574693 -1.383518

31 17 0 -3.081621 -2.656297 -0.605505

32 17 0 -3.060767 3.356734 -0.426038

33 1 0 -3.420062 1.044878 -0.825412

34 1 0 -0.318688 -2.497137 -0.135865

---------------------------------------------------------------------

**b1**

**
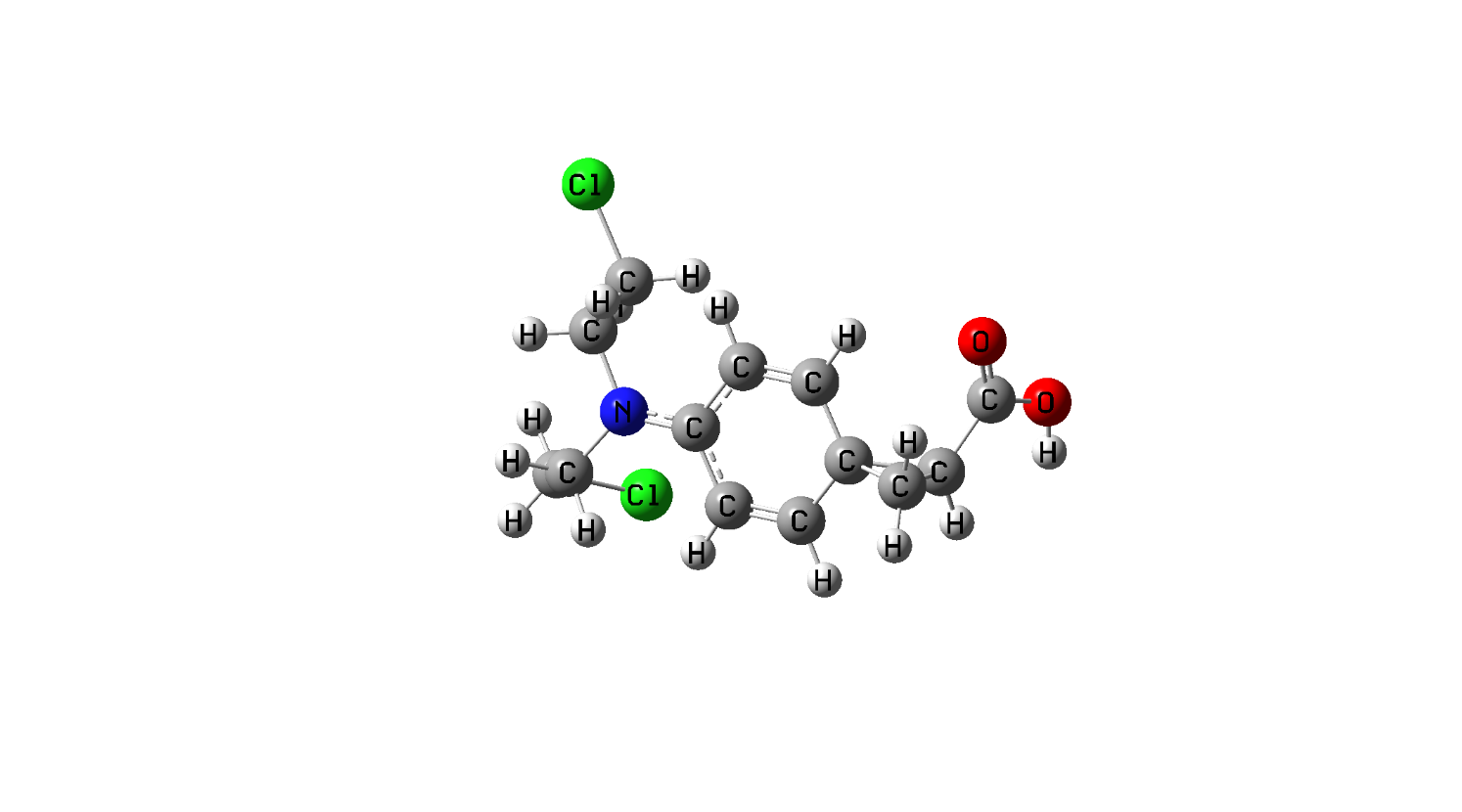
**

Zero-point correction= 0.274855 (Hartree/Particle)

Thermal correction to Energy= 0.292726

Thermal correction to Enthalpy= 0.293670

Thermal correction to Gibbs Free Energy= 0.225958

Sum of electronic and zero-point Energies= -1630.117319

Sum of electronic and thermal Energies= -1630.099448

Sum of electronic and thermal Enthalpies= -1630.098504

Sum of electronic and thermal Free Energies= -1630.166216

---------------------------------------------------------------------

Center Atomic Atomic Coordinates (Angstroms)

Number Number Type X Y Z

---------------------------------------------------------------------

1 6 0 0.065203 0.918351 0.432865

2 6 0 -0.713339 -0.269553 0.621787

3 6 0 -0.025365 -1.515475 0.514100

4 6 0 1.327168 -1.557881 0.255906

5 6 0 2.091816 -0.379066 0.094410

6 6 0 1.417996 0.857855 0.173812

7 1 0 -0.390997 1.894533 0.527529

8 1 0 -0.560156 -2.450808 0.595862

9 1 0 1.960980 1.792831 0.074855

10 6 0 3.532376 -0.513761 -0.224718

11 6 0 4.467738 0.569779 -0.420757

12 1 0 3.536264 -0.653110 -1.361503

13 1 0 3.981113 -1.430604 0.169240

14 1 0 6.269837 -1.062501 -1.267951

15 6 0 5.918449 0.477413 -0.140690

16 8 0 6.684257 -0.524921 -0.575220

17 8 0 6.328449 1.344118 0.599962

18 7 0 -2.047731 -0.192176 0.906549

19 6 0 -2.835354 -1.349461 1.350033

20 6 0 -3.846720 -1.878698 0.333393

21 1 0 -3.399860 -1.041598 2.239770

22 1 0 -2.166911 -2.147333 1.668323

23 1 0 -4.391519 -2.716568 0.772231

24 1 0 -4.566459 -1.115902 0.032768

25 6 0 -2.769804 1.089352 0.848743

26 6 0 -3.044842 1.548584 -0.590104

27 1 0 -2.219479 1.861627 1.393803

28 1 0 -3.717924 0.960398 1.373646

29 1 0 -2.125012 1.665911 -1.164096

30 1 0 -3.699061 0.854150 -1.117777

31 17 0 -3.865964 3.152625 -0.554527

32 17 0 -3.058592 -2.484182 -1.177673

33 1 0 4.118031 1.560744 -0.696683

34 1 0 1.809138 -2.528261 0.170936

---------------------------------------------------------------------

**TS1**

**
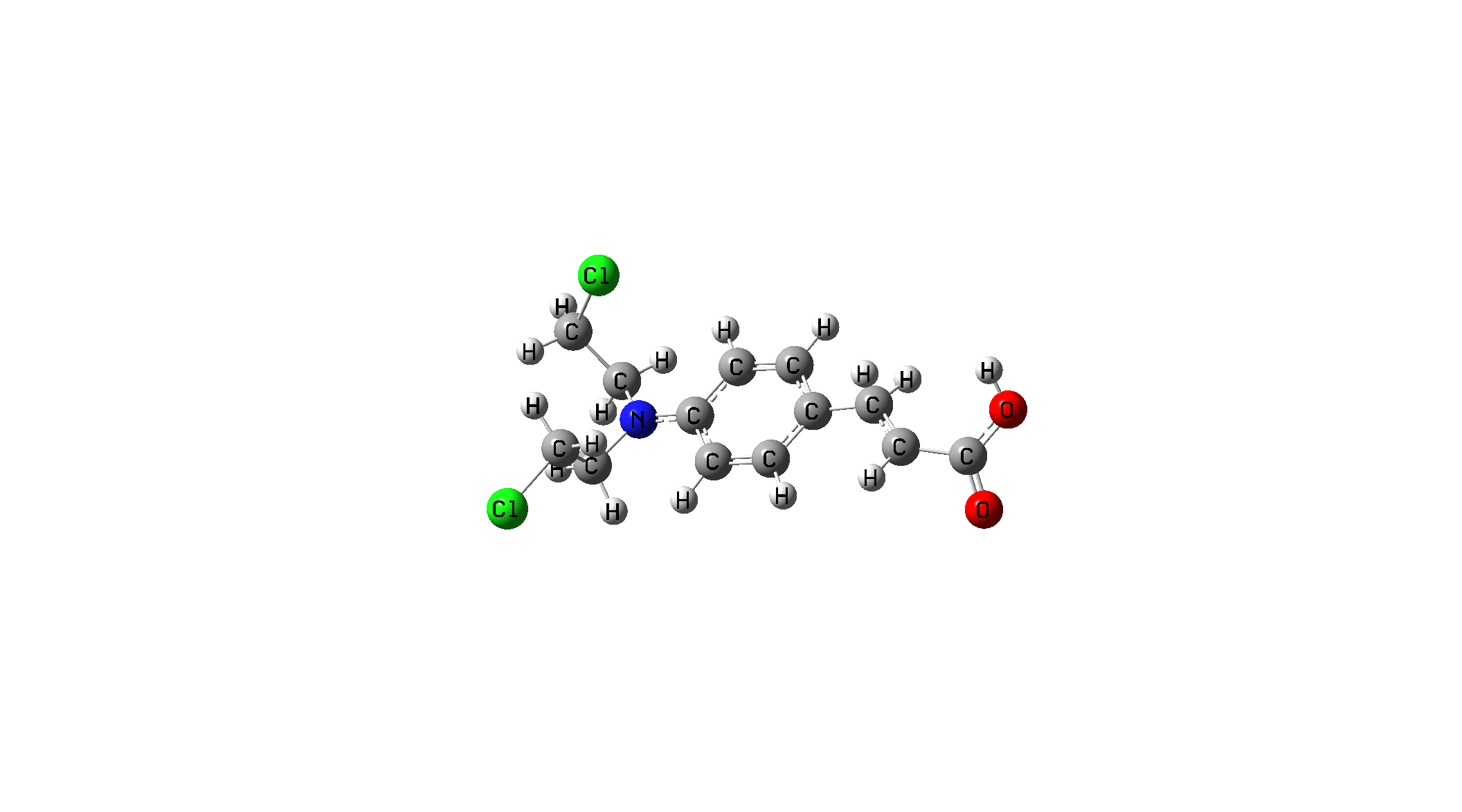
**

Zero-point correction= 0.268797 (Hartree/Particle)

Thermal correction to Energy= 0.287427

Thermal correction to Enthalpy= 0.288372

Thermal correction to Gibbs Free Energy= 0.217696

Sum of electronic and zero-point Energies= -1630.062410

Sum of electronic and thermal Energies= -1630.043779

Sum of electronic and thermal Enthalpies= -1630.042835

Sum of electronic and thermal Free Energies= -1630.113510

---------------------------------------------------------------------

Center Atomic Atomic Coordinates (Angstroms)

Number Number Type X Y Z

---------------------------------------------------------------------

1 6 0 -0.254454 0.623681 -0.792664

2 6 0 0.671283 -0.468928 -0.762580

3 6 0 0.131341 -1.787711 -0.588832

4 6 0 -1.229132 -1.989829 -0.488567

5 6 0 -2.119718 -0.910520 -0.638164

6 6 0 -1.612086 0.405778 -0.695723

7 1 0 0.094155 1.640094 -0.916211

8 1 0 0.784208 -2.645114 -0.507968

9 1 0 -2.300260 1.244898 -0.729176

10 6 0 -3.707458 -1.136601 -0.651473

11 6 0 -4.518185 -0.612723 0.540514

12 1 0 -3.868169 -2.220263 -0.702529

13 1 0 -4.102543 -0.692421 -1.566956

14 1 0 -6.517504 0.152456 1.781084

15 6 0 -5.206256 0.684246 0.451192

16 8 0 -6.267944 0.915522 1.235738

17 8 0 -4.783559 1.534400 -0.312921

18 7 0 2.013010 -0.254285 -0.887649

19 6 0 2.987294 -1.339528 -1.065381

20 6 0 3.880761 -1.619543 0.145126

21 1 0 3.644319 -1.051314 -1.895567

22 1 0 2.473430 -2.247471 -1.374354

23 1 0 4.577352 -2.421015 -0.107395

24 1 0 4.454776 -0.742029 0.445601

25 6 0 2.571159 1.109301 -0.890682

26 6 0 2.594053 1.745215 0.508743

27 1 0 2.009793 1.741807 -1.583854

28 1 0 3.589704 1.049417 -1.277169

29 1 0 1.593975 1.827096 0.935569

30 1 0 3.223724 1.182280 1.198059

31 17 0 3.268939 3.408860 0.384360

32 17 0 2.938929 -2.159386 1.588784

33 1 0 -4.396193 -1.097419 1.509051

34 1 0 -1.605169 -2.998463 -0.349683

---------------------------------------------------------------------

**c**

**
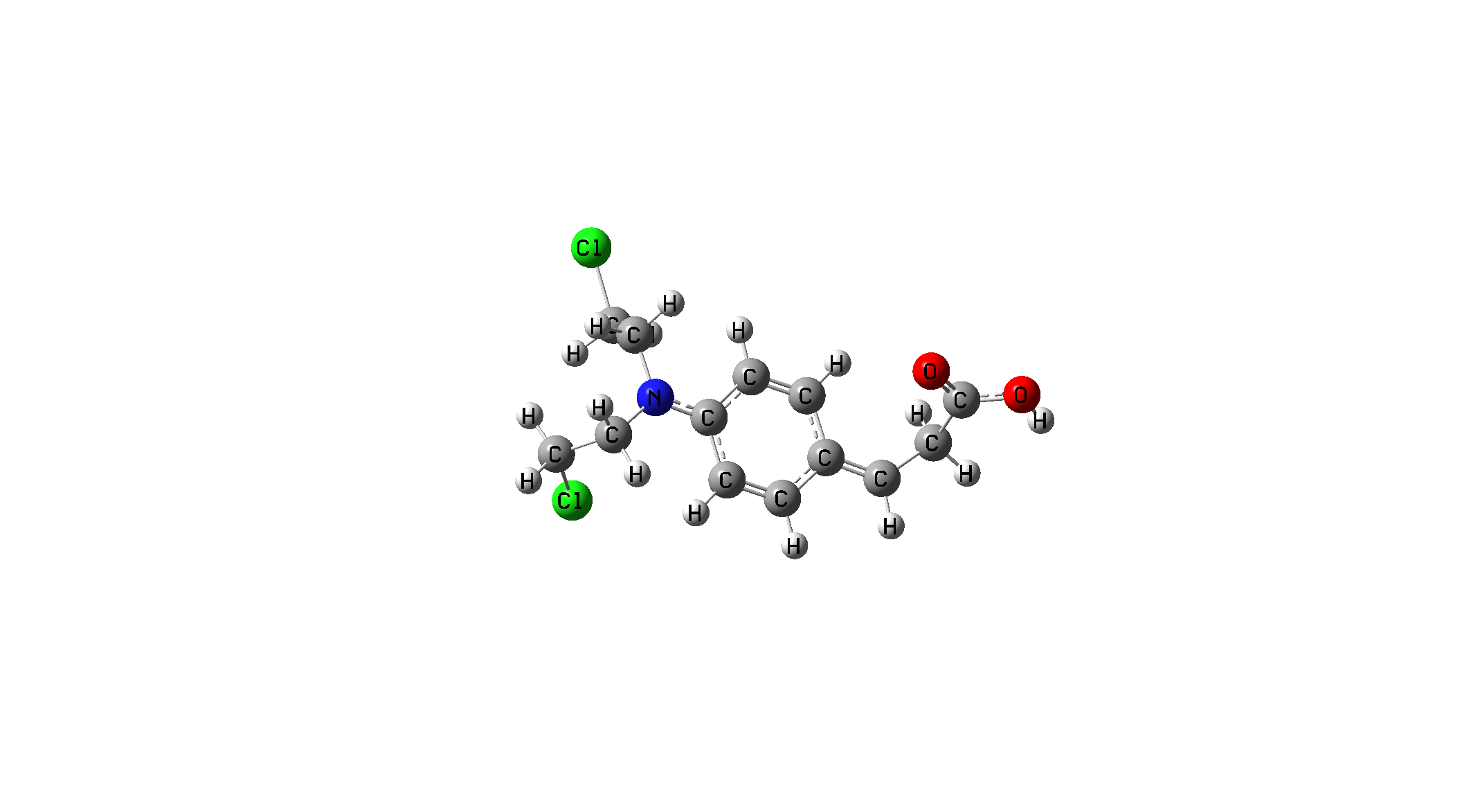
**

Zero-point correction= 0.274591 (Hartree/Particle)

Thermal correction to Energy= 0.292944

Thermal correction to Enthalpy= 0.293888

Thermal correction to Gibbs Free Energy= 0.224052

Sum of electronic and zero-point Energies= -1630.137393

Sum of electronic and thermal Energies= -1630.119040

Sum of electronic and thermal Enthalpies= -1630.118096

Sum of electronic and thermal Free Energies= -1630.187932

---------------------------------------------------------------------

Center Atomic Atomic Coordinates (Angstroms)

Number Number Type X Y Z

---------------------------------------------------------------------

1 6 0 0.066025 0.916843 0.431750

2 6 0 -0.715675 -0.271804 0.620668

3 6 0 -0.024392 -1.518628 0.511865

4 6 0 1.325728 -1.562738 0.256595

5 6 0 2.099157 -0.383651 0.084392

6 6 0 1.414940 0.857816 0.174140

7 1 0 -0.391425 1.892540 0.523606

8 1 0 -0.560524 -2.453374 0.590420

9 1 0 1.957298 1.793635 0.080596

10 6 0 3.511241 -0.504627 -0.164975

11 6 0 4.448182 0.549830 -0.439394

12 1 0 3.874606 -0.274083 -1.383038

13 1 0 3.959675 -1.470734 0.072351

14 1 0 6.280593 -1.062972 -1.277081

15 6 0 5.919726 0.473433 -0.136536

16 8 0 6.685132 -0.528980 -0.576535

17 8 0 6.323928 1.338215 0.598447

18 7 0 -2.046499 -0.195322 0.904149

19 6 0 -2.836588 -1.353276 1.346231

20 6 0 -3.847789 -1.879961 0.329340

21 1 0 -3.399561 -1.046216 2.236982

22 1 0 -2.168857 -2.152133 1.663653

23 1 0 -4.393257 -2.718628 0.765781

24 1 0 -4.567318 -1.116618 0.029492

25 6 0 -2.769862 1.087392 0.849924

26 6 0 -3.045474 1.548544 -0.587439

27 1 0 -2.220320 1.858338 1.397256

28 1 0 -3.717608 0.956544 1.375009

29 1 0 -2.126474 1.666849 -1.162525

30 1 0 -3.700173 0.855178 -1.115941

31 17 0 -3.866452 3.153173 -0.549866

32 17 0 -3.059447 -2.482885 -1.183206

33 1 0 4.088258 1.548517 -0.670986

34 1 0 1.807065 -2.533400 0.172950

---------------------------------------------------------------------

**c1**

**
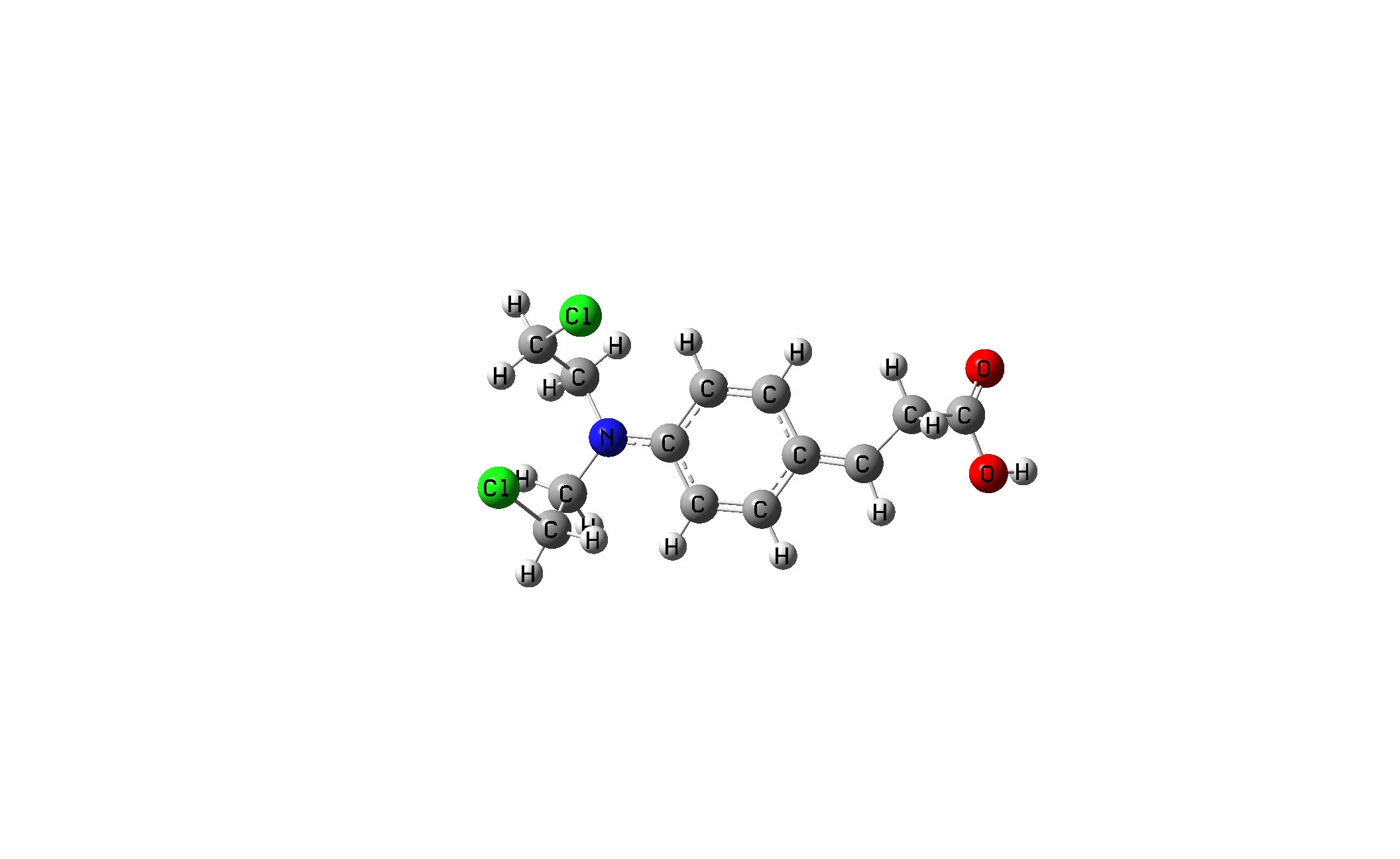
**

Zero-point correction= 0.274859 (Hartree/Particle)

Thermal correction to Energy= 0.293068

Thermal correction to Enthalpy= 0.294013

Thermal correction to Gibbs Free Energy= 0.223872

Sum of electronic and zero-point Energies= -1630.146752

Sum of electronic and thermal Energies= -1630.128542

Sum of electronic and thermal Enthalpies= -1630.127598

Sum of electronic and thermal Free Energies= -1630.197738

---------------------------------------------------------------------

Center Atomic Atomic Coordinates (Angstroms)

Number Number Type X Y Z

---------------------------------------------------------------------

1 6 0 0.050259 -1.649361 -0.518572

2 6 0 -0.578732 -0.381928 -0.683930

3 6 0 0.254406 0.771361 -0.554013

4 6 0 1.597750 0.658853 -0.271225

5 6 0 2.216687 -0.604549 -0.116910

6 6 0 1.406005 -1.749144 -0.267927

7 1 0 -0.516531 -2.565760 -0.606401

8 1 0 -0.177065 1.760174 -0.618744

9 1 0 2.177484 1.572941 -0.173496

10 1 0 1.844028 -2.738776 -0.162833

11 6 0 3.600541 -0.773170 0.255677

12 6 0 4.311270 0.108721 1.295756

13 1 0 3.913496 -1.815016 0.317630

14 1 0 4.570198 -0.389203 2.234134

15 1 0 3.772894 1.034108 1.526939

16 6 0 5.506897 0.408354 0.453370

17 8 0 4.681752 -0.117295 -0.840933

18 8 0 6.602171 0.796892 0.426731

19 7 0 -1.920009 -0.271535 -0.953033

20 6 0 -2.491700 0.947001 -1.550145

21 6 0 -3.380063 1.798026 -0.646992

22 1 0 -3.114335 0.632998 -2.398213

23 1 0 -1.689709 1.553258 -1.973338

24 1 0 -3.860335 2.573126 -1.247200

25 1 0 -4.140517 1.200400 -0.145483

26 6 0 -2.780752 -1.462722 -0.960819

27 6 0 -3.079236 -2.082693 0.405513

28 1 0 -2.337216 -2.242845 -1.595934

29 1 0 -3.724572 -1.185064 -1.432808

30 1 0 -3.593533 -3.034379 0.258603

31 1 0 -2.177068 -2.252916 0.992536

32 17 0 -4.166330 -1.062839 1.429741

33 17 0 -2.461374 2.655327 0.660169

34 1 0 5.092442 -0.643875 -1.557700

---------------------------------------------------------------------

**TS2**

**
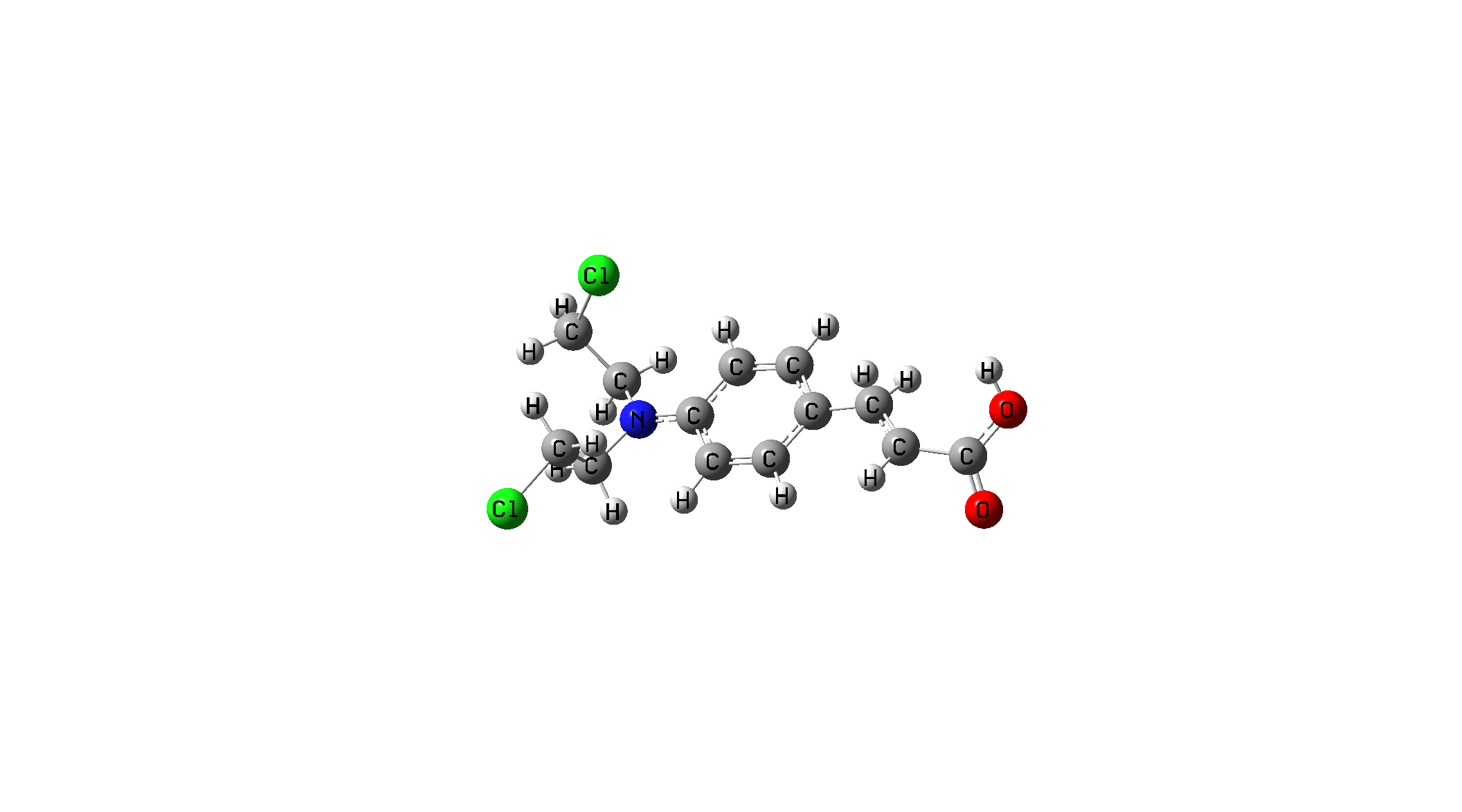
**

Zero-point correction= 0.271938 (Hartree/Particle)

Thermal correction to Energy= 0.289800

Thermal correction to Enthalpy= 0.290745

Thermal correction to Gibbs Free Energy= 0.222895

Sum of electronic and zero-point Energies= -1630.081158

Sum of electronic and thermal Energies= -1630.063296

Sum of electronic and thermal Enthalpies= -1630.062351

Sum of electronic and thermal Free Energies= -1630.130201

---------------------------------------------------------------------

Center Atomic Atomic Coordinates (Angstroms)

Number Number Type X Y Z

---------------------------------------------------------------------

1 6 0 -0.073654 -1.588350 0.297263

2 6 0 0.541710 -0.310804 0.568683

3 6 0 -0.271401 0.870042 0.378181

4 6 0 -1.563863 0.777952 -0.037456

5 6 0 -2.189579 -0.495859 -0.304220

6 6 0 -1.372098 -1.669445 -0.106863

7 1 0 0.479216 -2.505412 0.441555

8 1 0 0.163280 1.849707 0.510927

9 1 0 -2.127111 1.692676 -0.188946

10 1 0 -1.816426 -2.645321 -0.279990

11 6 0 -3.484494 -0.644189 -0.732883

12 6 0 -4.472922 0.435116 -1.009851

13 1 0 -3.855988 -1.653870 -0.904642

14 1 0 -5.032665 0.197980 -1.929608

15 1 0 -4.009348 1.409693 -1.196199

16 6 0 -5.498714 0.566437 0.112274

17 8 0 -4.804605 -0.785939 0.407481

18 8 0 -6.631080 0.778129 0.276990

19 7 0 1.815789 -0.228219 0.995169

20 6 0 2.381416 1.014474 1.571953

21 6 0 3.356078 1.786402 0.686641

22 1 0 2.924754 0.716387 2.475795

23 1 0 1.567467 1.658259 1.901686

24 1 0 3.777150 2.606610 1.271019

25 1 0 4.161272 1.153900 0.315670

26 6 0 2.671745 -1.434843 1.101423

27 6 0 3.106553 -2.077352 -0.218255

28 1 0 2.147377 -2.189949 1.699135

29 1 0 3.558677 -1.150885 1.668236

30 1 0 3.573957 -3.037608 0.008312

31 1 0 2.274007 -2.243027 -0.901131

32 17 0 4.327634 -1.108325 -1.125243

33 17 0 2.562988 2.521835 -0.760856

34 1 0 -4.935906 -1.606264 0.888548

---------------------------------------------------------------------

**d**

**
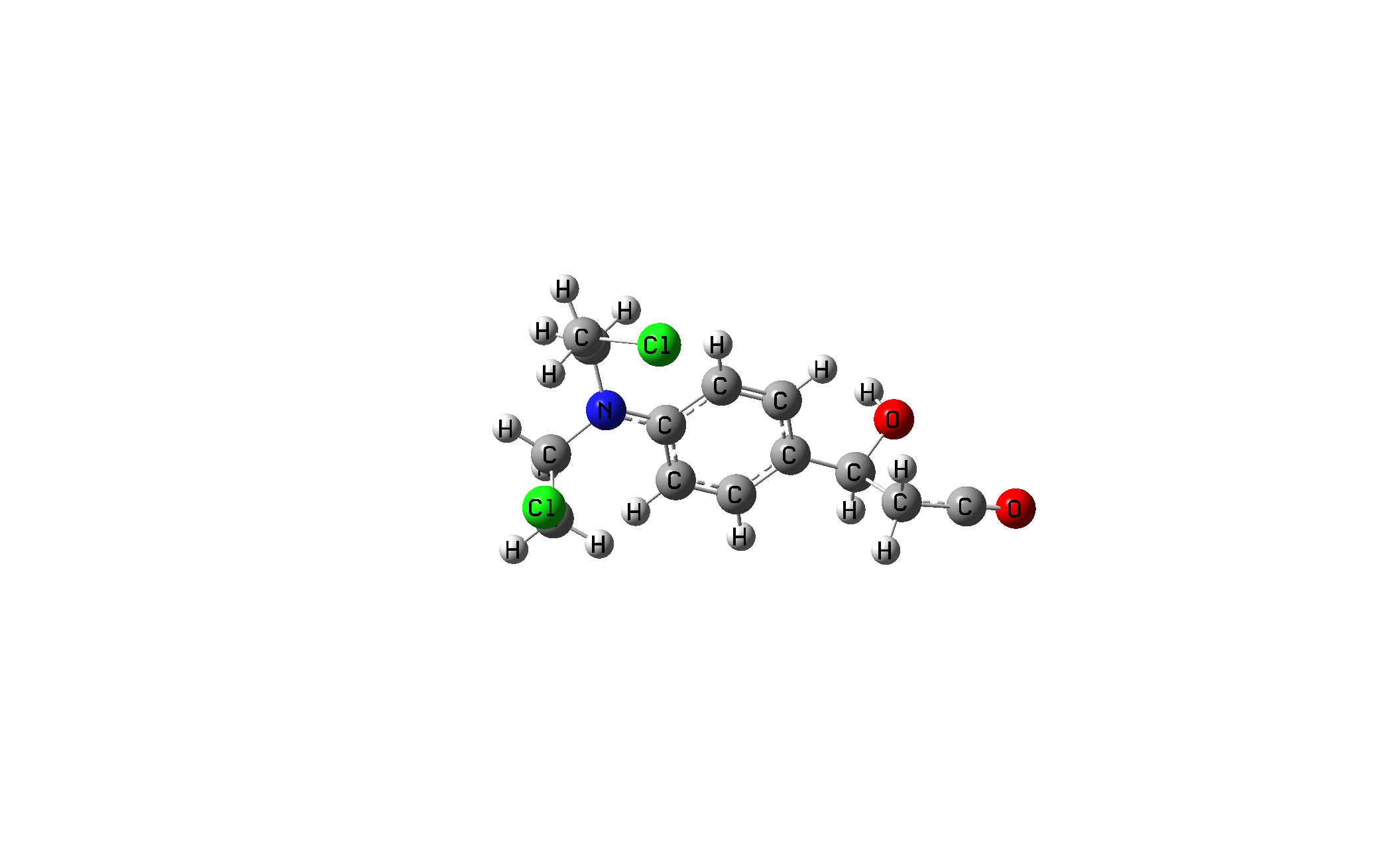
**

Zero-point correction= 0.272108 (Hartree/Particle)

Thermal correction to Energy= 0.290809

Thermal correction to Enthalpy= 0.291753

Thermal correction to Gibbs Free Energy= 0.222031

Sum of electronic and zero-point Energies= -1630.088847

Sum of electronic and thermal Energies= -1630.070146

Sum of electronic and thermal Enthalpies= -1630.069202

Sum of electronic and thermal Free Energies= -1630.138923

---------------------------------------------------------------------

Center Atomic Atomic Coordinates (Angstroms)

Number Number Type X Y Z

---------------------------------------------------------------------

1 6 0 0.146983 -1.383109 -0.959273

2 6 0 -0.601586 -0.173702 -0.894033

3 6 0 0.141665 1.029864 -0.718953

4 6 0 1.520263 1.014629 -0.614458

5 6 0 2.253688 -0.187551 -0.695233

6 6 0 1.528444 -1.380852 -0.863353

7 1 0 -0.346913 -2.332357 -1.115412

8 1 0 -0.370995 1.974439 -0.605228

9 1 0 2.021768 1.965885 -0.443789

10 1 0 2.055859 -2.328543 -0.943717

11 6 0 3.724174 -0.238027 -0.537058

12 6 0 4.066732 -0.220425 1.145035

13 1 0 4.135007 -1.197935 -0.859760

14 1 0 3.644064 -1.109565 1.625314

15 1 0 3.624044 0.687326 1.568629

16 6 0 5.461360 -0.193031 1.361228

17 8 0 4.478274 0.795692 -1.083417

18 8 0 6.599110 -0.188786 1.351765

19 7 0 -1.973692 -0.168439 -0.991352

20 6 0 -2.720721 1.053345 -1.325988

21 6 0 -3.537472 1.681183 -0.199409

22 1 0 -3.431329 0.796098 -2.122556

23 1 0 -2.039528 1.788699 -1.756394

24 1 0 -4.149725 2.487670 -0.607392

25 1 0 -4.176711 0.950078 0.294039

26 6 0 -2.722920 -1.429373 -1.059037

27 6 0 -2.790162 -2.245274 0.233777

28 1 0 -2.299842 -2.076497 -1.841166

29 1 0 -3.739832 -1.192600 -1.376421

30 1 0 -3.245637 -3.213708 0.018128

31 1 0 -1.807664 -2.405280 0.676897

32 17 0 -3.804756 -1.473826 1.517098

33 17 0 -2.513101 2.420569 1.101894

34 1 0 3.905110 1.469440 -1.480833

---------------------------------------------------------------------

**TS3**

**
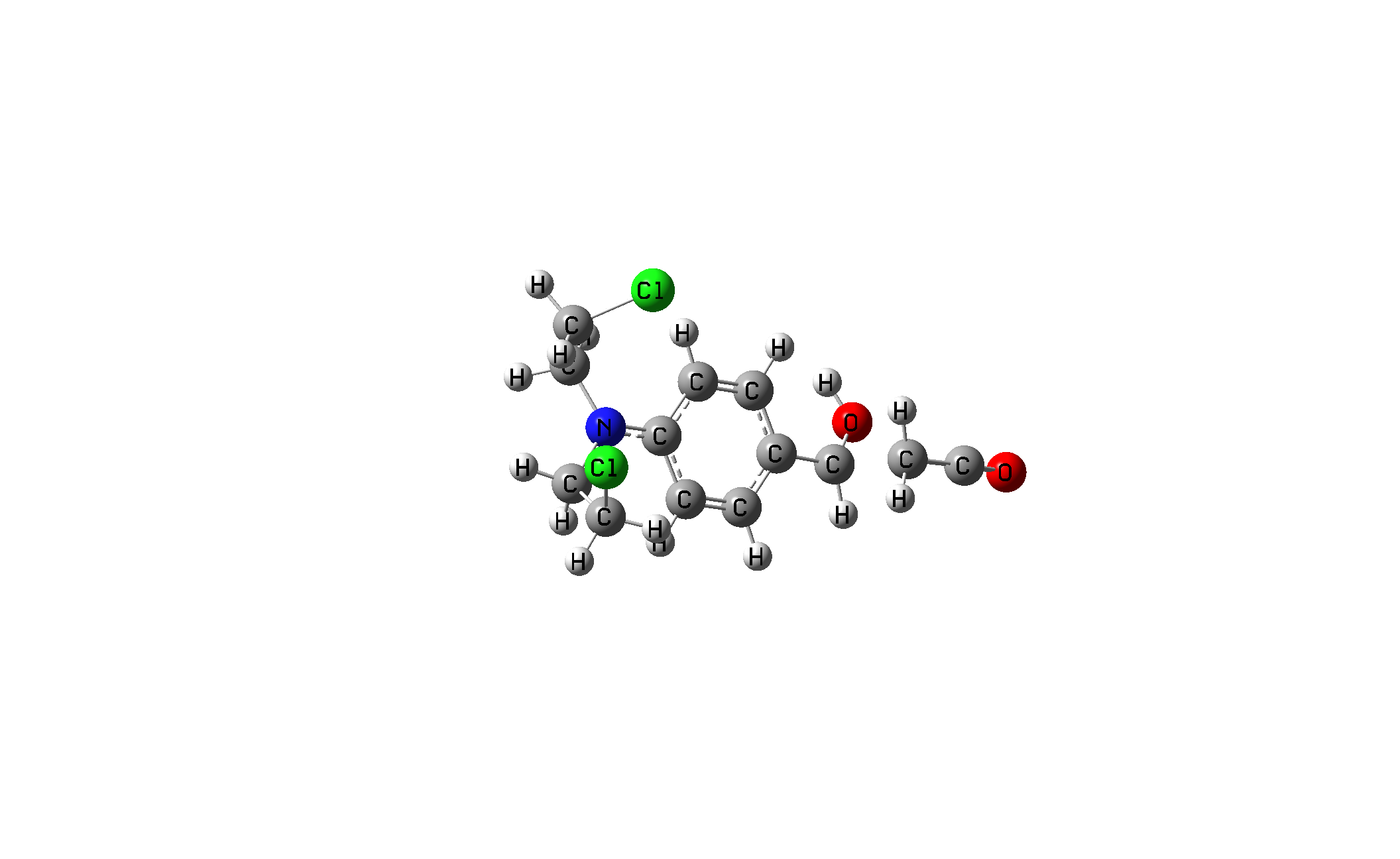
**

Zero-point correction= 0.270429 (Hartree/Particle)

Thermal correction to Energy= 0.289067

Thermal correction to Enthalpy= 0.290011

Thermal correction to Gibbs Free Energy= 0.220032

Sum of electronic and zero-point Energies= -1630.085031

Sum of electronic and thermal Energies= -1630.066393

Sum of electronic and thermal Enthalpies= -1630.065449

Sum of electronic and thermal Free Energies= -1630.135428

---------------------------------------------------------------------

Center Atomic Atomic Coordinates (Angstroms)

Number Number Type X Y Z

---------------------------------------------------------------------

1 6 0 0.026322 -1.573918 -0.804569

2 6 0 -0.667285 -0.332783 -0.865445

3 6 0 0.127910 0.849574 -0.840440

4 6 0 1.506460 0.786588 -0.766187

5 6 0 2.183652 -0.449066 -0.718237

6 6 0 1.409812 -1.622059 -0.731022

7 1 0 -0.511147 -2.511592 -0.842285

8 1 0 -0.344099 1.821889 -0.831533

9 1 0 2.067863 1.717509 -0.749200

10 1 0 1.896110 -2.594700 -0.706341

11 6 0 3.662171 -0.523487 -0.605094

12 6 0 4.316179 0.028479 1.428683

13 1 0 4.014106 -1.549311 -0.770956

14 1 0 4.042110 -0.724969 2.175586

15 1 0 3.874250 0.995790 1.699129

16 6 0 5.734644 0.191157 1.431302

17 8 0 4.396433 0.408998 -1.382707

18 8 0 6.860479 0.268863 1.320124

19 7 0 -2.041070 -0.275736 -0.944019

20 6 0 -2.737974 0.925406 -1.426875

21 6 0 -3.517328 1.725240 -0.385803

22 1 0 -3.466694 0.601857 -2.182201

23 1 0 -2.030025 1.570930 -1.948545

24 1 0 -4.106476 2.492889 -0.891163

25 1 0 -4.175323 1.088918 0.205075

26 6 0 -2.845295 -1.499091 -0.847104

27 6 0 -2.913528 -2.151602 0.535038

28 1 0 -2.472039 -2.254032 -1.554879

29 1 0 -3.859256 -1.254721 -1.168357

30 1 0 -3.421337 -3.114167 0.448837

31 1 0 -1.927230 -2.307420 0.971023

32 17 0 -3.851169 -1.180135 1.738833

33 17 0 -2.451655 2.592439 0.798190

34 1 0 4.084937 0.392603 -2.300271

---------------------------------------------------------------------

**p2**

**
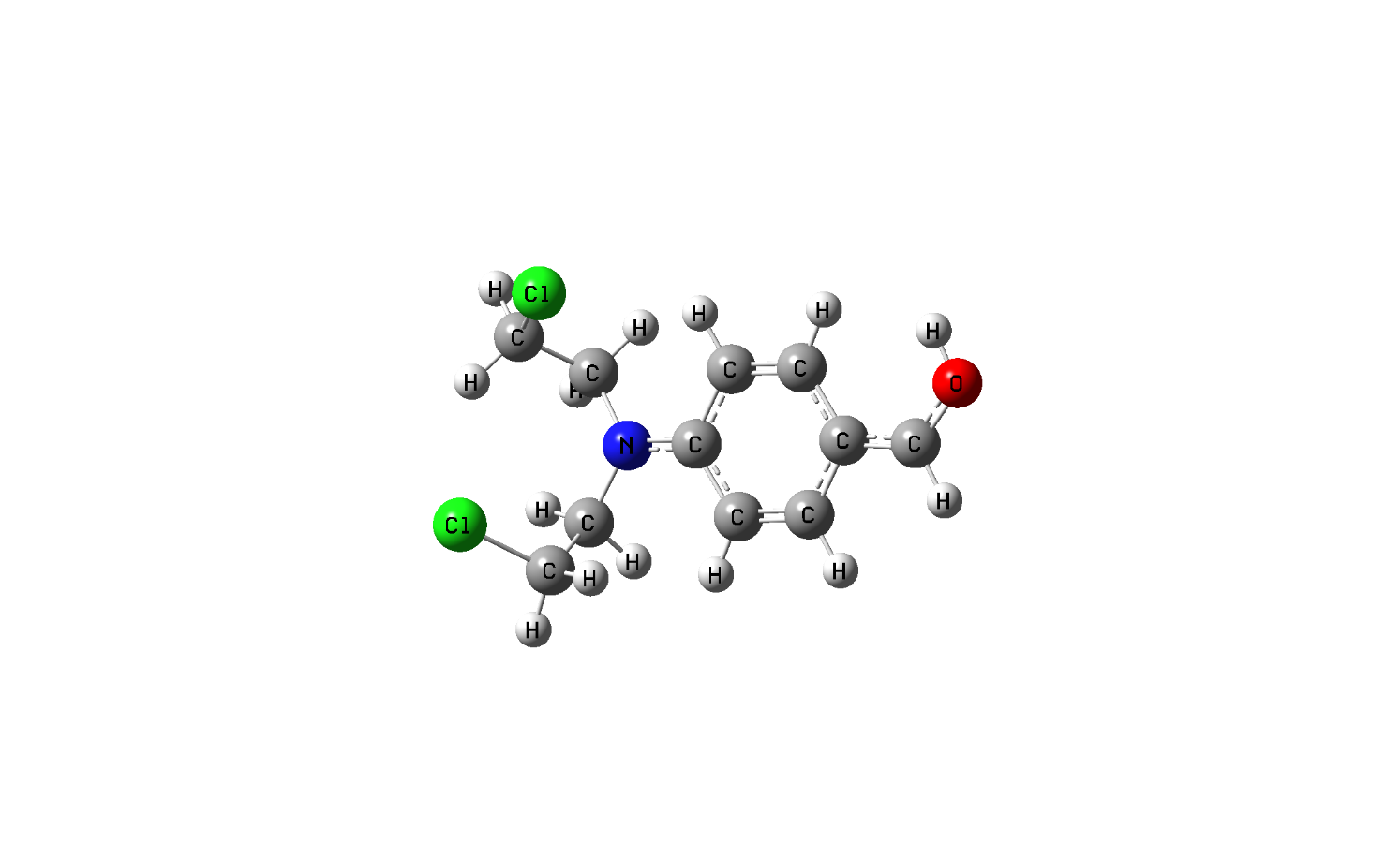
**

Zero-point correction= 0.236890 (Hartree/Particle)

Thermal correction to Energy= 0.251847

Thermal correction to Enthalpy= 0.252792

Thermal correction to Gibbs Free Energy= 0.192481

Sum of electronic and zero-point Energies= -1477.520836

Sum of electronic and thermal Energies= -1477.505879

Sum of electronic and thermal Enthalpies= -1477.504935

Sum of electronic and thermal Free Energies= -1477.565246

---------------------------------------------------------------------

Center Atomic Atomic Coordinates (Angstroms)

Number Number Type X Y Z

---------------------------------------------------------------------

1 6 0 -1.115826 -1.509039 0.420966

2 6 0 -0.421468 -0.269403 0.630630

3 6 0 -1.174658 0.945909 0.445774

4 6 0 -2.494019 0.916116 0.096766

5 6 0 -3.188004 -0.321725 -0.096035

6 6 0 -2.442212 -1.530321 0.082735

7 1 0 -0.608619 -2.453684 0.554996

8 1 0 -0.681450 1.903488 0.525151

9 1 0 -2.997097 1.868803 -0.049603

10 1 0 -2.947459 -2.482824 -0.046493

11 6 0 -4.527774 -0.431059 -0.446656

12 1 0 -4.986397 -1.408464 -0.578340

13 8 0 -5.383805 0.543083 -0.655647

14 7 0 0.882434 -0.243197 0.993220

15 6 0 1.526014 0.971166 1.540181

16 6 0 2.501371 1.696621 0.617493

17 1 0 2.091205 0.655043 2.424946

18 1 0 0.756765 1.654419 1.898713

19 1 0 2.991517 2.490805 1.183700

20 1 0 3.253902 1.024062 0.208080

21 6 0 1.673864 -1.489383 1.079620

22 6 0 2.012324 -2.168949 -0.249189

23 1 0 1.140581 -2.212177 1.710040

24 1 0 2.601037 -1.248994 1.600955

25 1 0 2.447541 -3.147015 -0.035226

26 1 0 1.139702 -2.302552 -0.887671

27 17 0 3.228515 -1.264900 -1.229763

28 17 0 1.688997 2.484097 -0.793917

29 1 0 -5.003218 1.430853 -0.547772

---------------------------------------------------------------------

**TS4**

**
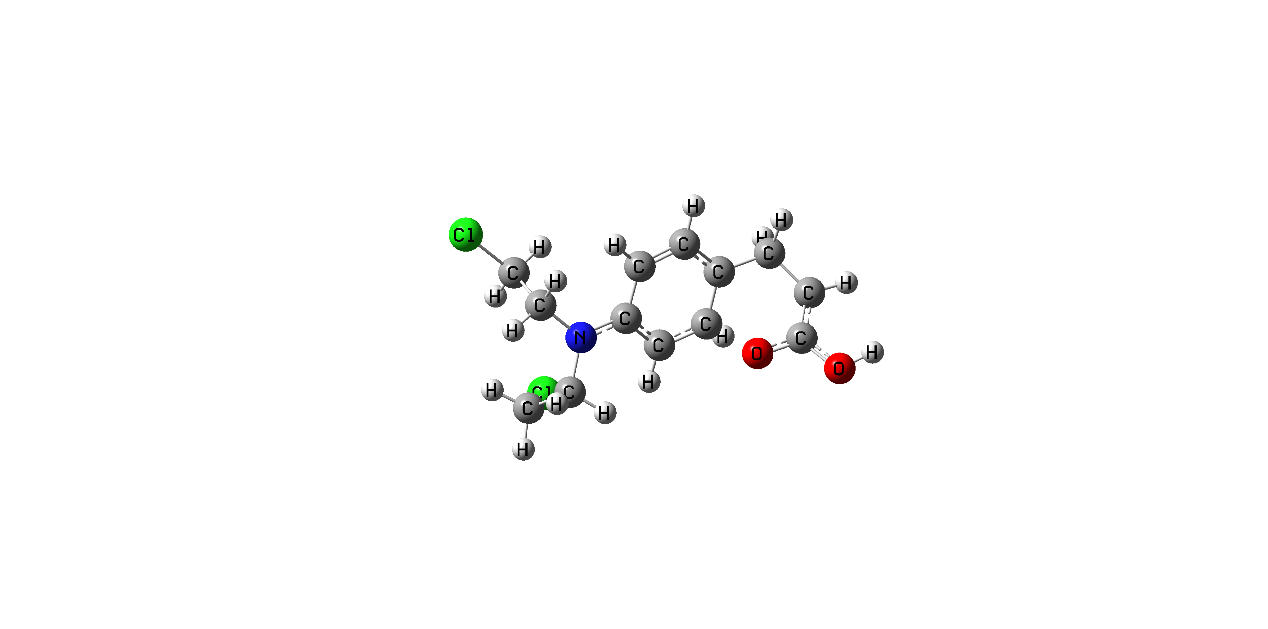
**

Zero-point correction= 0.270362 (Hartree/Particle)

Thermal correction to Energy= 0.287850

Thermal correction to Enthalpy= 0.288794

Thermal correction to Gibbs Free Energy= 0.222661

Sum of electronic and zero-point Energies= -1630.032351

Sum of electronic and thermal Energies= -1630.014864

Sum of electronic and thermal Enthalpies= -1630.013919

Sum of electronic and thermal Free Energies= -1630.080053

---------------------------------------------------------------------

Center Atomic Atomic Coordinates (Angstroms)

Number Number Type X Y Z

---------------------------------------------------------------------

1 6 0 0.046676 -1.554485 -0.276900

2 6 0 -0.232905 -0.118167 -0.350597

3 6 0 0.743309 0.763697 0.110319

4 6 0 1.997227 0.281305 0.546880

5 6 0 2.246472 -1.159700 0.612215

6 6 0 1.236531 -2.047559 0.200681

7 1 0 -0.687125 -2.261112 -0.642475

8 1 0 0.546012 1.820931 0.206519

9 1 0 1.413800 -3.118423 0.212297

10 6 0 3.589583 -1.567919 1.049805

11 6 0 4.678148 -0.627517 0.696180

12 1 0 3.822487 -2.609459 0.781430

13 1 0 3.502551 -1.600330 2.161237

14 1 0 6.283815 0.415873 -0.891549

15 6 0 4.447585 0.278383 -0.342659

16 8 0 5.427184 0.835920 -1.064159

17 8 0 3.263941 0.600728 -0.751617

18 7 0 -1.442274 0.280424 -0.849778

19 6 0 -1.680494 1.671718 -1.260322

20 6 0 -2.655858 2.462247 -0.389155

21 1 0 -2.104681 1.643181 -2.273133

22 1 0 -0.724633 2.188460 -1.332164

23 1 0 -2.762529 3.468733 -0.797718

24 1 0 -3.642970 1.998902 -0.350496

25 6 0 -2.580990 -0.640922 -1.011352

26 6 0 -3.206782 -1.048020 0.329290

27 1 0 -2.288780 -1.528908 -1.578471

28 1 0 -3.329099 -0.129723 -1.619353

29 1 0 -2.491563 -1.569283 0.968700

30 1 0 -3.594988 -0.186628 0.873536

31 17 0 -4.577868 -2.175854 0.027125

32 17 0 -2.090412 2.634060 1.320430

33 1 0 5.678048 -0.808616 1.071208

34 1 0 2.536323 0.872390 1.290337

---------------------------------------------------------------------

**e**

**
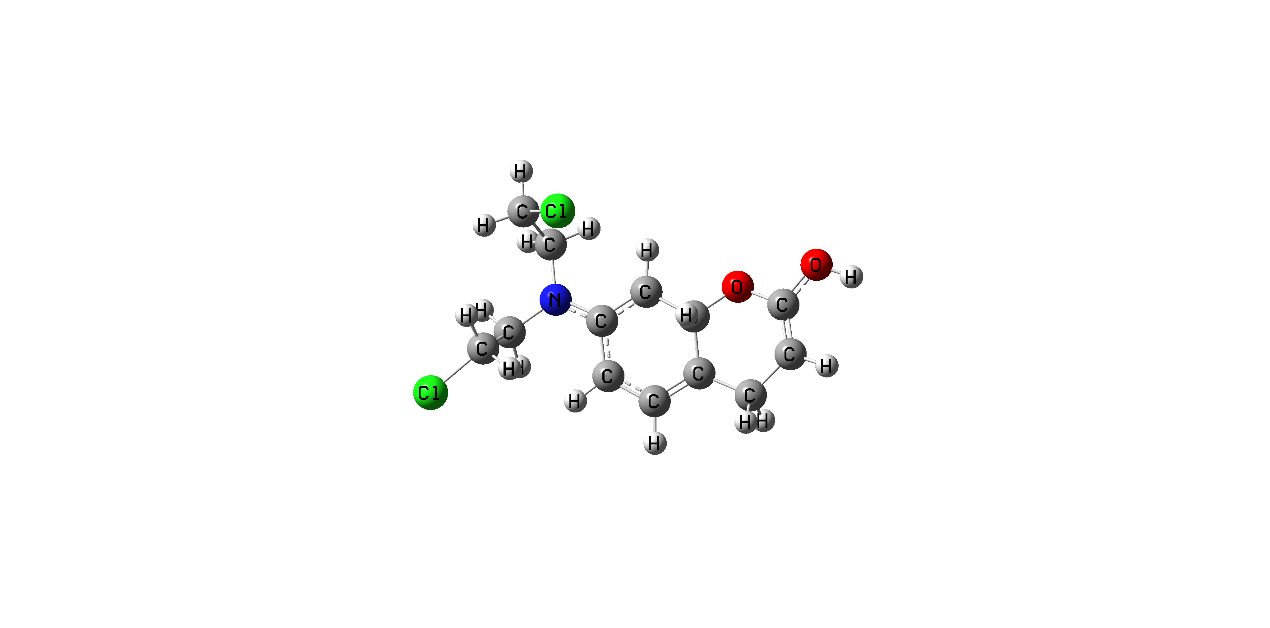
**

Zero-point correction= 0.272285 (Hartree/Particle)

Thermal correction to Energy= 0.289990

Thermal correction to Enthalpy= 0.290934

Thermal correction to Gibbs Free Energy= 0.223864

Sum of electronic and zero-point Energies= -1630.058442

Sum of electronic and thermal Energies= -1630.040737

Sum of electronic and thermal Enthalpies= -1630.039793

Sum of electronic and thermal Free Energies= -1630.106863

---------------------------------------------------------------------

Center Atomic Atomic Coordinates (Angstroms)

Number Number Type X Y Z

---------------------------------------------------------------------

1 6 0 0.219350 -1.668917 0.311897

2 6 0 0.383908 -0.265230 0.551617

3 6 0 -0.781892 0.510008 0.457949

4 6 0 -2.011805 -0.100274 0.036820

5 6 0 -2.186657 -1.518600 -0.091089

6 6 0 -1.008944 -2.255253 0.011844

7 1 0 1.078327 -2.326230 0.367515

8 1 0 -0.794263 1.585231 0.565917

9 1 0 -1.047024 -3.330929 -0.131875

10 6 0 -3.548909 -2.097325 -0.359455

11 6 0 -4.647200 -1.077941 -0.163021

12 1 0 -3.696032 -2.951112 0.316218

13 1 0 -3.580104 -2.521958 -1.375319

14 1 0 -6.198726 0.976999 -0.060163

15 6 0 -4.426516 0.237839 -0.058273

16 8 0 -5.281361 1.264971 0.052909

17 8 0 -3.156863 0.817414 -0.029744

18 7 0 1.606547 0.269389 0.877039

19 6 0 1.748360 1.643350 1.369915

20 6 0 2.340766 2.641659 0.377937

21 1 0 2.416061 1.619117 2.240536

22 1 0 0.787323 2.003928 1.740088

23 1 0 2.439934 3.618542 0.854730

24 1 0 3.320725 2.327418 0.015893

25 6 0 2.821387 -0.555921 0.859907

26 6 0 3.348244 -0.809009 -0.558260

27 1 0 2.652341 -1.506944 1.376614

28 1 0 3.586066 -0.034049 1.438287

29 1 0 2.602859 -1.284516 -1.198580

30 1 0 3.673618 0.115445 -1.036494

31 17 0 4.771016 -1.914880 -0.484221

32 17 0 1.297308 2.872996 -1.087477

33 1 0 -5.669838 -1.436587 -0.136321

34 1 0 -1.973351 0.206552 -1.069494

---------------------------------------------------------------------

**TS5**

**
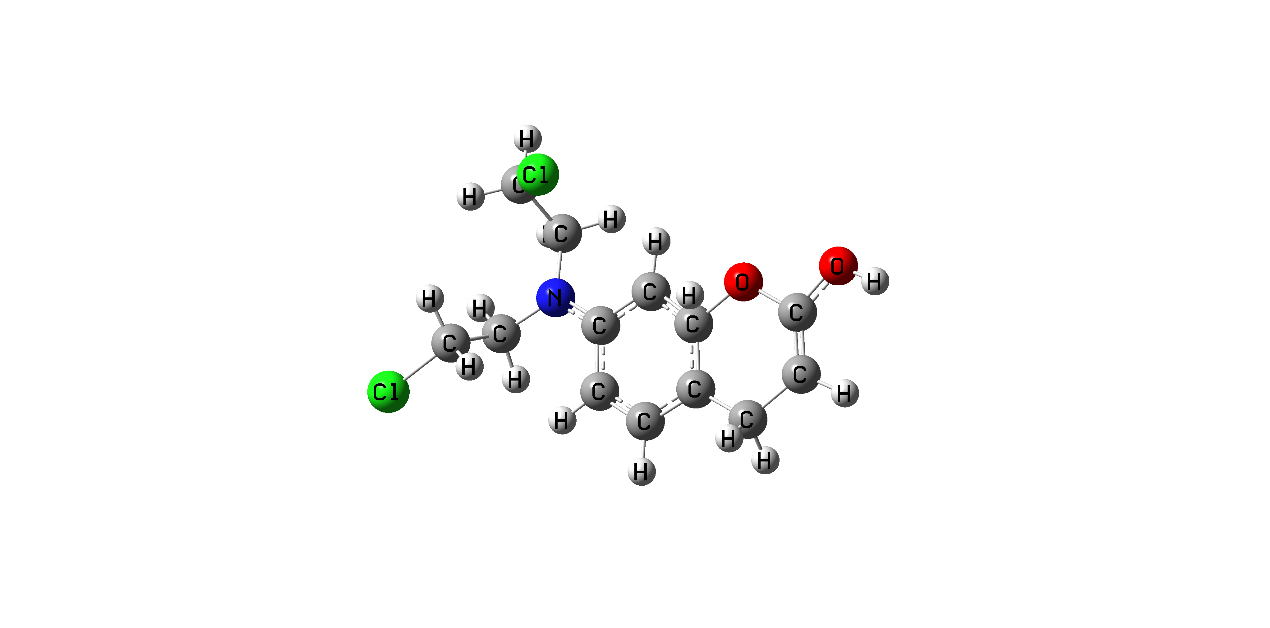
**

Zero-point correction= 0.268170 (Hartree/Particle)

Thermal correction to Energy= 0.286053

Thermal correction to Enthalpy= 0.286997

Thermal correction to Gibbs Free Energy= 0.219432

Sum of electronic and zero-point Energies= -1630.004738

Sum of electronic and thermal Energies= -1629.986855

Sum of electronic and thermal Enthalpies= -1629.985911

Sum of electronic and thermal Free Energies= -1630.053476

---------------------------------------------------------------------

Center Atomic Atomic Coordinates (Angstroms)

Number Number Type X Y Z

---------------------------------------------------------------------

1 6 0 0.212789 -1.625119 0.285574

2 6 0 0.374333 -0.213598 0.524212

3 6 0 -0.792895 0.540504 0.428861

4 6 0 -2.049498 -0.060399 -0.035080

5 6 0 -2.177380 -1.531274 -0.099130

6 6 0 -1.000027 -2.253861 -0.007429

7 1 0 1.083624 -2.267419 0.343930

8 1 0 -0.823464 1.611666 0.574718

9 1 0 -1.007478 -3.331995 -0.138573

10 6 0 -3.527340 -2.079835 -0.402381

11 6 0 -4.582558 -1.012033 -0.493192

12 1 0 -3.766496 -2.811621 0.390648

13 1 0 -3.481389 -2.682252 -1.322179

14 1 0 -6.147989 0.933849 -0.001592

15 6 0 -4.372209 0.226080 -0.012192

16 8 0 -5.236382 1.240180 0.111338

17 8 0 -3.157252 0.653173 0.463166

18 7 0 1.600518 0.308233 0.867038

19 6 0 1.748277 1.695528 1.320809

20 6 0 2.338061 2.661433 0.295595

21 1 0 2.418217 1.694264 2.189732

22 1 0 0.789159 2.068835 1.683146

23 1 0 2.419705 3.658321 0.732739

24 1 0 3.325548 2.346041 -0.044430

25 6 0 2.806974 -0.529228 0.880248

26 6 0 3.360544 -0.804086 -0.524404

27 1 0 2.620607 -1.472807 1.405072

28 1 0 3.565578 -0.009018 1.468168

29 1 0 2.618206 -1.260133 -1.182586

30 1 0 3.722610 0.108595 -0.998789

31 17 0 4.748366 -1.949129 -0.412306

32 17 0 1.304431 2.815114 -1.186229

33 1 0 -5.556349 -1.279913 -0.884884

34 1 0 -2.766619 0.582709 -0.674263

---------------------------------------------------------------------

**f**

**
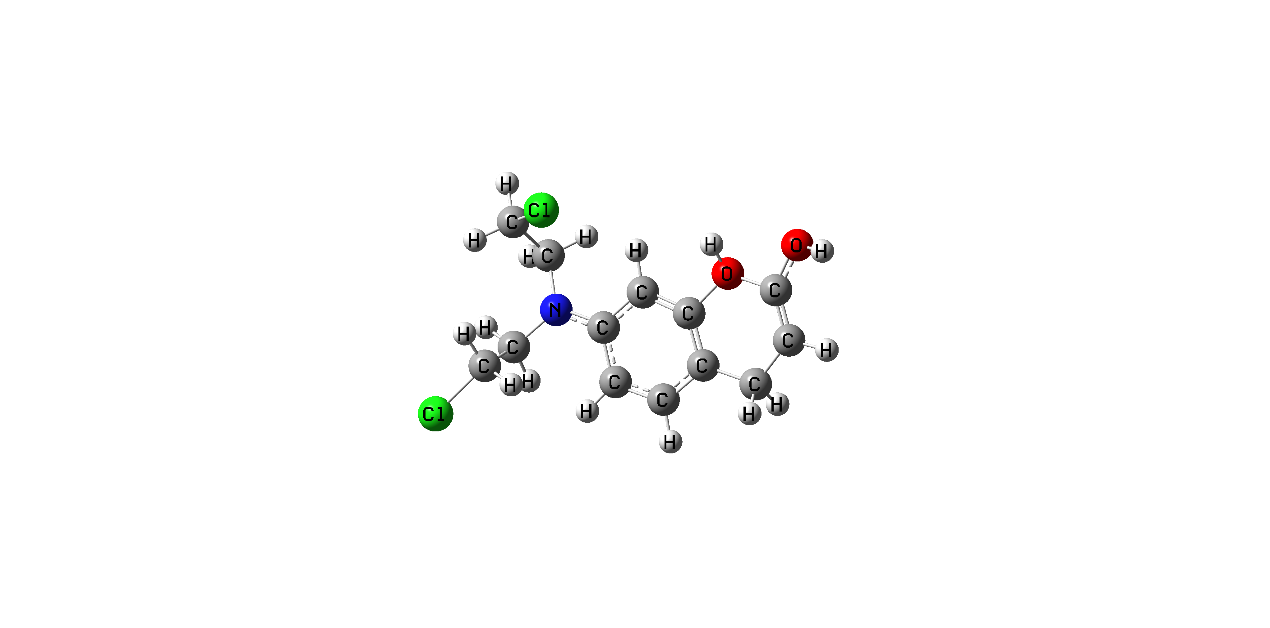
**

Zero-point correction= 0.273894 (Hartree/Particle)

Thermal correction to Energy= 0.291935

Thermal correction to Enthalpy= 0.292879

Thermal correction to Gibbs Free Energy= 0.224983

Sum of electronic and zero-point Energies= -1630.063844

Sum of electronic and thermal Energies= -1630.045803

Sum of electronic and thermal Enthalpies= -1630.044859

Sum of electronic and thermal Free Energies= -1630.112755

---------------------------------------------------------------------

Center Atomic Atomic Coordinates (Angstroms)

Number Number Type X Y Z

---------------------------------------------------------------------

1 6 0 0.226919 -1.672973 0.306359

2 6 0 0.386339 -0.276003 0.549370

3 6 0 -0.791039 0.502775 0.450072

4 6 0 -1.964164 -0.152306 0.101804

5 6 0 -2.181127 -1.507677 -0.097116

6 6 0 -0.998286 -2.255504 0.008213

7 1 0 1.084421 -2.329683 0.361976

8 1 0 -0.797669 1.576681 0.575483

9 1 0 -1.042359 -3.331057 -0.138396

10 6 0 -3.543151 -2.102228 -0.362163

11 6 0 -4.639237 -1.086900 -0.163812

12 1 0 -3.705165 -2.950973 0.312928

13 1 0 -3.592862 -2.515614 -1.379819

14 1 0 -6.200516 0.978974 -0.046528

15 6 0 -4.446737 0.222564 -0.060172

16 8 0 -5.278392 1.258634 0.059928

17 8 0 -3.126300 0.808769 -0.068703

18 7 0 1.609067 0.267874 0.876310

19 6 0 1.749740 1.638706 1.372344

20 6 0 2.340523 2.641513 0.383516

21 1 0 2.418931 1.615922 2.242532

22 1 0 0.788602 1.998035 1.743686

23 1 0 2.439061 3.617983 0.861438

24 1 0 3.319701 2.328869 0.018207

25 6 0 2.824782 -0.556207 0.856412

26 6 0 3.350854 -0.805090 -0.563005

27 1 0 2.655108 -1.508924 1.368618

28 1 0 3.590391 -0.034861 1.434807

29 1 0 2.605308 -1.280513 -1.201539

30 1 0 3.676801 0.120506 -1.038730

31 17 0 4.775736 -1.909865 -0.492888

32 17 0 1.295629 2.875620 -1.081245

33 1 0 -5.666167 -1.438384 -0.128881

34 1 0 -2.705220 0.776845 -0.980423

---------------------------------------------------------------------

**TS6**

**
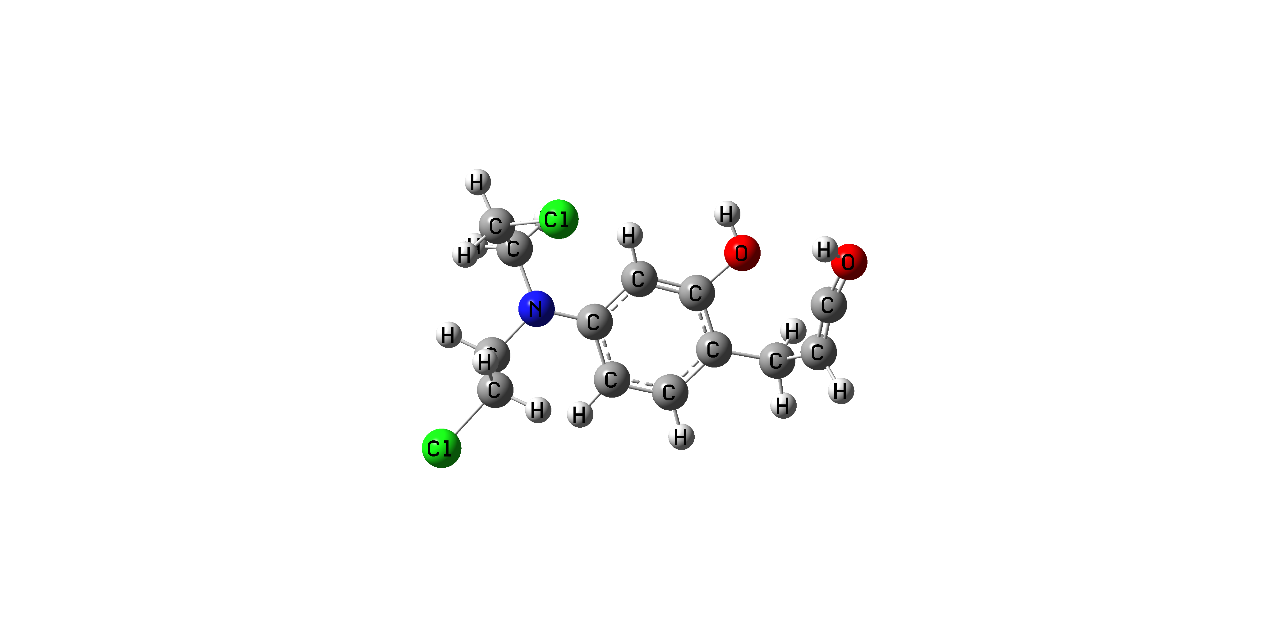
**

Zero-point correction= 0.271171 (Hartree/Particle)

Thermal correction to Energy= 0.289315

Thermal correction to Enthalpy= 0.290259

Thermal correction to Gibbs Free Energy= 0.222470

Sum of electronic and zero-point Energies= -1630.043831

Sum of electronic and thermal Energies= -1630.025687

Sum of electronic and thermal Enthalpies= -1630.024743

Sum of electronic and thermal Free Energies= -1630.092531

---------------------------------------------------------------------

Center Atomic Atomic Coordinates (Angstroms)

Number Number Type X Y Z

---------------------------------------------------------------------

1 6 0 0.314506 -1.722522 0.410210

2 6 0 0.473203 -0.322155 0.606622

3 6 0 -0.705756 0.459262 0.532815

4 6 0 -1.898714 -0.191329 0.295048

5 6 0 -2.113809 -1.541654 0.120282

6 6 0 -0.928306 -2.296066 0.187113

7 1 0 1.173237 -2.379313 0.440662

8 1 0 -0.677985 1.537927 0.613842

9 1 0 -0.989408 -3.372957 0.057869

10 6 0 -3.474220 -2.153448 -0.108662

11 6 0 -4.934032 -1.112257 -0.369144

12 1 0 -3.735968 -2.811585 0.731197

13 1 0 -3.452905 -2.796405 -0.997723

14 1 0 -6.299315 1.110667 -0.945189

15 6 0 -4.751851 0.192978 -0.263976

16 8 0 -5.520418 1.281618 -0.393632

17 8 0 -3.080800 0.707345 0.222548

18 7 0 1.708196 0.245051 0.851111

19 6 0 1.853565 1.597021 1.390752

20 6 0 2.375851 2.652950 0.419073

21 1 0 2.571183 1.554884 2.221386

22 1 0 0.908392 1.923003 1.829304

23 1 0 2.529987 3.596765 0.945345

24 1 0 3.314734 2.351655 -0.047459

25 6 0 2.931604 -0.560051 0.755135

26 6 0 3.358030 -0.808592 -0.696510

27 1 0 2.815050 -1.512790 1.282447

28 1 0 3.728350 -0.022663 1.273620

29 1 0 2.579514 -1.310233 -1.272495

30 1 0 3.622922 0.121270 -1.201297

31 17 0 4.815200 -1.874772 -0.727478

32 17 0 1.220407 2.999753 -0.940349

33 1 0 -5.942038 -1.452454 -0.586365

34 1 0 -2.899116 1.545685 -0.253726

---------------------------------------------------------------------

**f1**

**
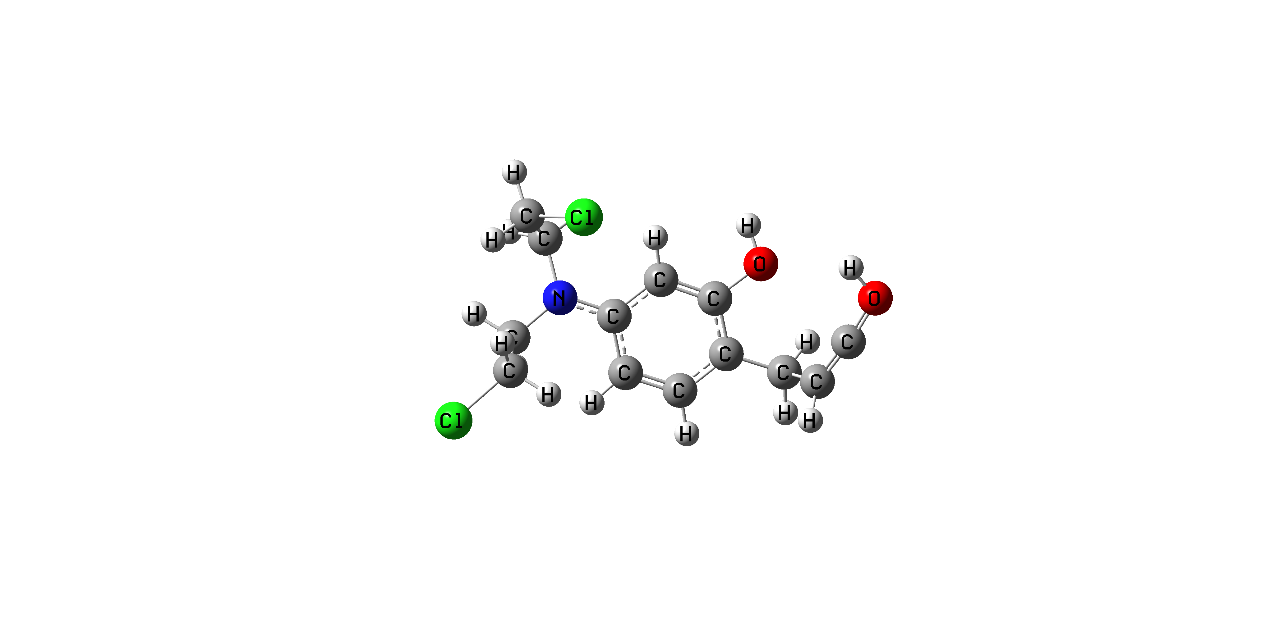
**

Zero-point correction= 0.271224 (Hartree/Particle)

Thermal correction to Energy= 0.290348

Thermal correction to Enthalpy= 0.291293

Thermal correction to Gibbs Free Energy= 0.220408

Sum of electronic and zero-point Energies= -1630.045316

Sum of electronic and thermal Energies= -1630.026191

Sum of electronic and thermal Enthalpies= -1630.025247

Sum of electronic and thermal Free Energies= -1630.096131

---------------------------------------------------------------------

Center Atomic Atomic Coordinates (Angstroms)

Number Number Type X Y Z

---------------------------------------------------------------------

1 6 0 -0.166143 -1.665290 -0.567714

2 6 0 -0.421979 -0.279533 -0.758691

3 6 0 0.700890 0.584725 -0.808379

4 6 0 1.981435 0.063828 -0.726970

5 6 0 2.244137 -1.305538 -0.575291

6 6 0 1.130632 -2.150177 -0.475919

7 1 0 -0.980031 -2.374561 -0.506600

8 1 0 0.573185 1.659082 -0.857421

9 1 0 1.281316 -3.218480 -0.346885

10 6 0 3.659136 -1.779763 -0.507427

11 6 0 4.412366 -1.231399 0.744631

12 1 0 4.244209 -1.460354 -1.374890

13 1 0 3.707009 -2.867311 -0.443960

14 1 0 4.570057 1.682709 1.833518

15 6 0 4.693234 -0.010513 1.019229

16 8 0 5.187819 1.090387 1.351791

17 8 0 3.107897 0.874361 -0.724943

18 7 0 -1.713119 0.203129 -0.876269

19 6 0 -2.006714 1.533089 -1.407088

20 6 0 -2.544752 2.549956 -0.402370

21 1 0 -2.777105 1.427712 -2.183999

22 1 0 -1.124787 1.932005 -1.909864

23 1 0 -2.808903 3.475171 -0.917922

24 1 0 -3.423748 2.178579 0.126310

25 6 0 -2.865247 -0.675677 -0.652093

26 6 0 -3.122696 -0.931526 0.837521

27 1 0 -2.741849 -1.626221 -1.181551

28 1 0 -3.744679 -0.196867 -1.088247

29 1 0 -2.259989 -1.382654 1.328568

30 1 0 -3.385769 -0.012658 1.362826

31 17 0 -4.506134 -2.078328 1.030910

32 17 0 -1.332441 2.995770 0.875669

33 1 0 4.711175 -1.950561 1.511122

34 1 0 2.875988 1.784936 -0.960379

---------------------------------------------------------------------

**TS7**

**
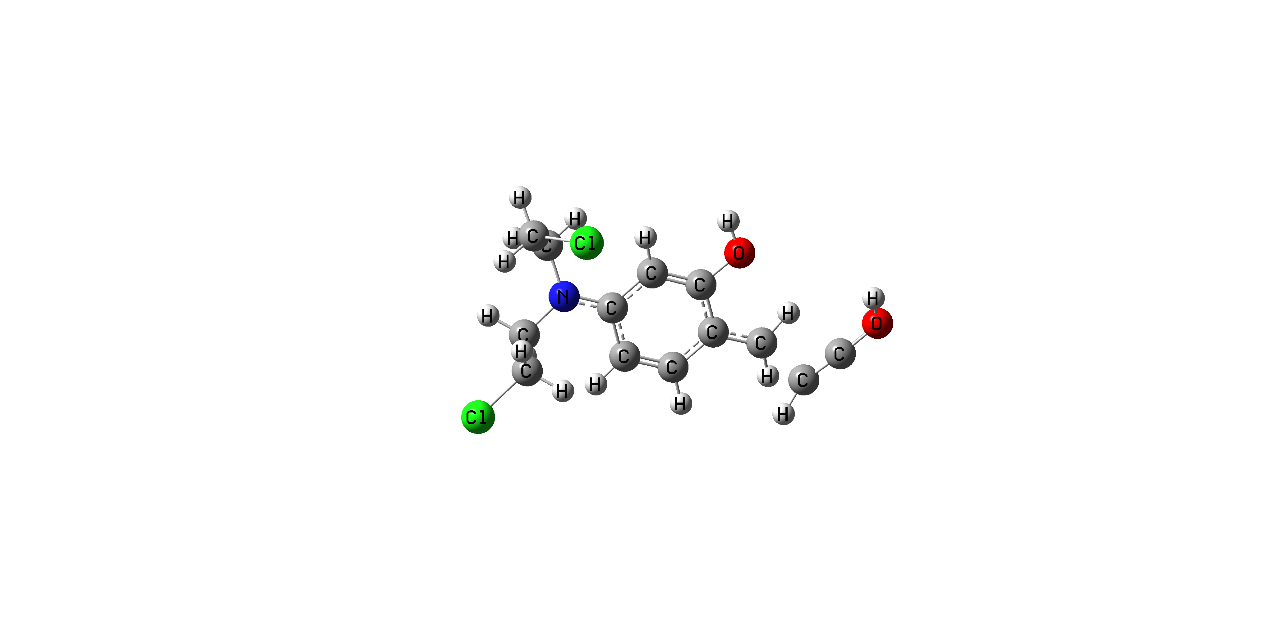
**

Zero-point correction= 0.269110 (Hartree/Particle)

Thermal correction to Energy= 0.288453

Thermal correction to Enthalpy= 0.289398

Thermal correction to Gibbs Free Energy= 0.217465

Sum of electronic and zero-point Energies= -1630.035273

Sum of electronic and thermal Energies= -1630.015929

Sum of electronic and thermal Enthalpies= -1630.014985

Sum of electronic and thermal Free Energies= -1630.086917

---------------------------------------------------------------------

Center Atomic Atomic Coordinates (Angstroms)

Number Number Type X Y Z

---------------------------------------------------------------------

1 6 0 -0.092822 -1.434933 -0.797399

2 6 0 -0.507470 -0.057121 -0.846069

3 6 0 0.510513 0.938225 -0.856009

4 6 0 1.845987 0.590717 -0.850500

5 6 0 2.269813 -0.781484 -0.859386

6 6 0 1.233961 -1.764197 -0.806814

7 1 0 -0.825043 -2.229708 -0.784621

8 1 0 0.252181 1.987479 -0.798742

9 1 0 1.517822 -2.812858 -0.801898

10 6 0 3.620066 -1.132253 -0.927059

11 6 0 4.414370 -1.371361 1.107181

12 1 0 4.351074 -0.388495 -1.215217

13 1 0 3.890786 -2.164454 -1.121925

14 1 0 6.061000 1.162743 1.568961

15 6 0 5.322188 -0.553329 1.276143

16 8 0 6.289270 0.287935 1.205275

17 8 0 2.834864 1.514288 -0.816208

18 7 0 -1.830429 0.265554 -0.881536

19 6 0 -2.314698 1.617785 -1.191271

20 6 0 -2.940343 2.376408 -0.023046

21 1 0 -3.088015 1.519926 -1.964167

22 1 0 -1.510306 2.201862 -1.635622

23 1 0 -3.306603 3.342587 -0.374772

24 1 0 -3.772659 1.831492 0.424555

25 6 0 -2.875903 -0.758016 -0.699038

26 6 0 -3.027588 -1.190275 0.764714

27 1 0 -2.676689 -1.625610 -1.333669

28 1 0 -3.818764 -0.333365 -1.048171

29 1 0 -2.104586 -1.610967 1.164881

30 1 0 -3.337999 -0.360842 1.400891

31 17 0 -4.294693 -2.468256 0.879119

32 17 0 -1.757283 2.708417 1.307116

33 1 0 3.845263 -2.172849 1.533103

34 1 0 2.477624 2.412670 -0.870053

---------------------------------------------------------------------

**TS8**

**
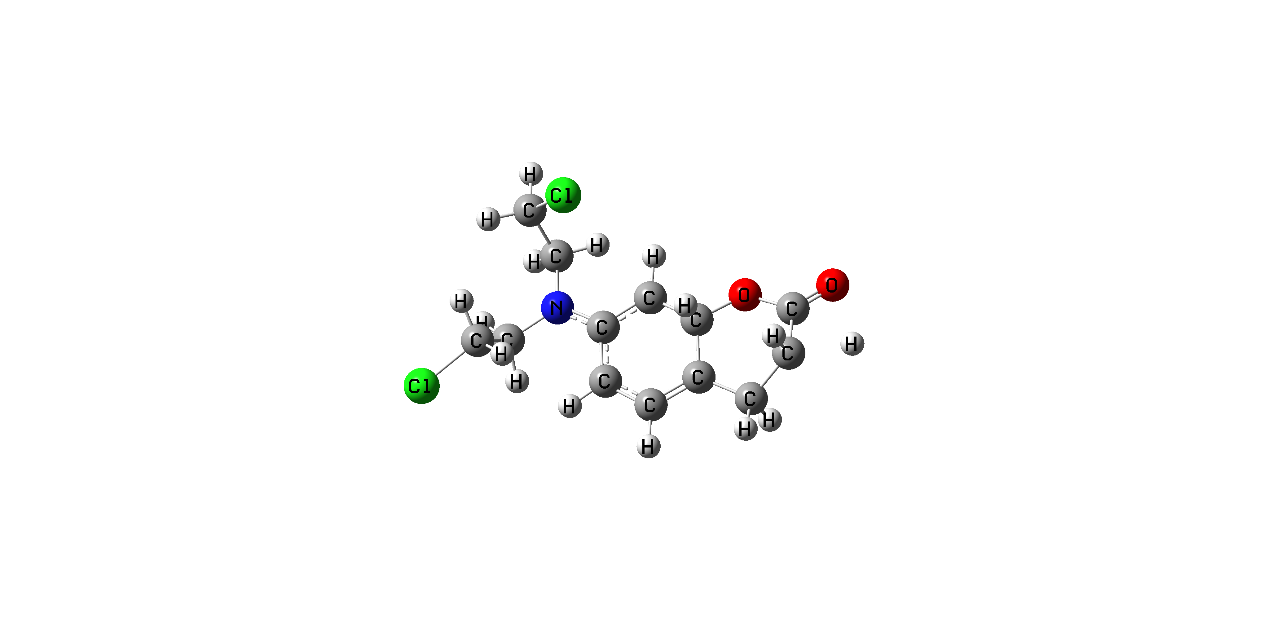
**

Zero-point correction= 0.266737 (Hartree/Particle)

Thermal correction to Energy= 0.284123

Thermal correction to Enthalpy= 0.285067

Thermal correction to Gibbs Free Energy= 0.218476

Sum of electronic and zero-point Energies= -1629.977270

Sum of electronic and thermal Energies= -1629.959884

Sum of electronic and thermal Enthalpies= -1629.958940

Sum of electronic and thermal Free Energies= -1630.025531

---------------------------------------------------------------------

Center Atomic Atomic Coordinates (Angstroms)

Number Number Type X Y Z

---------------------------------------------------------------------

1 6 0 0.194306 -1.603348 0.266812

2 6 0 0.373331 -0.198454 0.523173

3 6 0 -0.786094 0.573828 0.428078

4 6 0 -2.055974 -0.013359 -0.017980

5 6 0 -2.202998 -1.477756 -0.082798

6 6 0 -1.030326 -2.211842 -0.025003

7 1 0 1.058094 -2.256533 0.302487

8 1 0 -0.795567 1.649200 0.543995

9 1 0 -1.912041 0.146464 -1.134188

10 1 0 -1.053542 -3.288368 -0.166067

11 6 0 -3.581515 -2.008088 -0.279259

12 6 0 -4.531935 -0.912920 -0.801086

13 1 0 -3.938282 -2.357635 0.703779

14 1 0 -3.583599 -2.886893 -0.932151

15 1 0 -6.053473 -0.537799 -0.337739

16 6 0 -4.451096 0.357575 0.021770

17 8 0 -5.367877 1.046725 0.356391

18 8 0 -3.165773 0.733714 0.424407

19 7 0 1.602120 0.310552 0.865798

20 6 0 1.764927 1.698417 1.316115

21 6 0 2.360136 2.655291 0.285671

22 1 0 2.437951 1.691579 2.182270

23 1 0 0.810826 2.081531 1.682005

24 1 0 2.450367 3.653229 0.718598

25 1 0 3.344117 2.330861 -0.055754

26 6 0 2.797156 -0.544598 0.890329

27 6 0 3.365079 -0.822009 -0.508251

28 1 0 2.587448 -1.486991 1.408156

29 1 0 3.555001 -0.038703 1.491445

30 1 0 2.621543 -1.253235 -1.181764

31 1 0 3.757571 0.084891 -0.969376

32 17 0 4.720932 -2.002162 -0.383495

33 17 0 1.323947 2.811554 -1.194454

34 1 0 -4.284980 -0.665059 -1.842961

---------------------------------------------------------------------

**g**

**
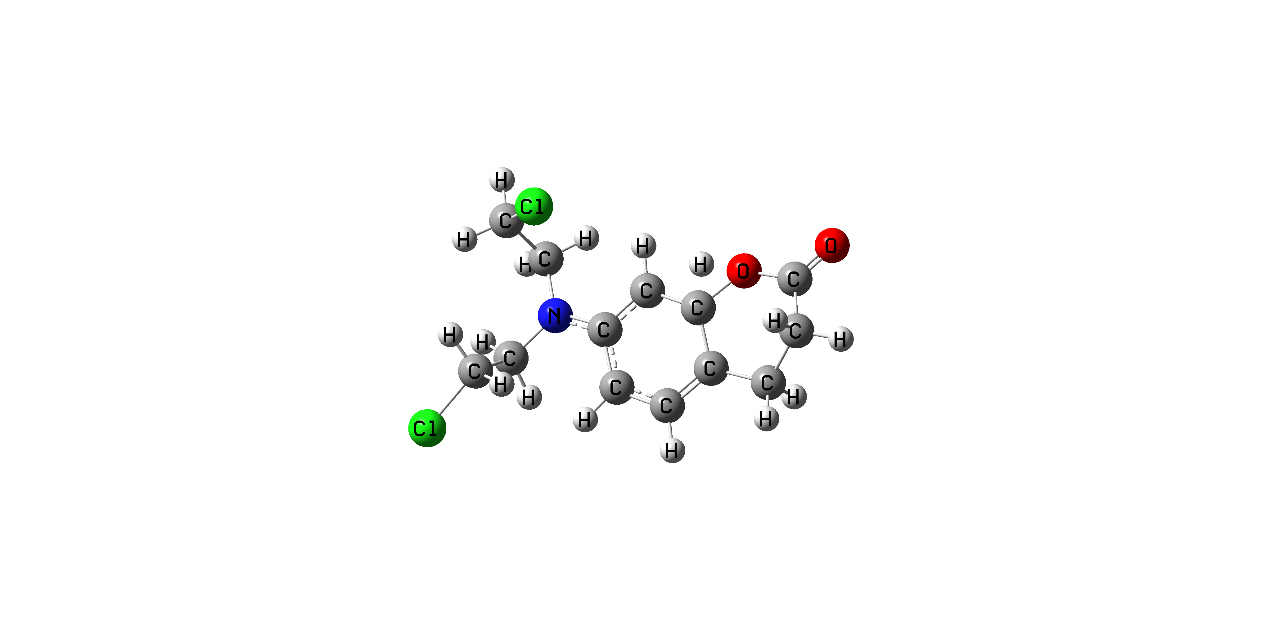
**

Zero-point correction= 0.272881 (Hartree/Particle)

Thermal correction to Energy= 0.290378

Thermal correction to Enthalpy= 0.291322

Thermal correction to Gibbs Free Energy= 0.224538

Sum of electronic and zero-point Energies= -1630.083089

Sum of electronic and thermal Energies= -1630.065592

Sum of electronic and thermal Enthalpies= -1630.064648

Sum of electronic and thermal Free Energies= -1630.131432

---------------------------------------------------------------------

Center Atomic Atomic Coordinates (Angstroms)

Number Number Type X Y Z

---------------------------------------------------------------------

1 6 0 0.174487 -1.585905 0.273309

2 6 0 0.370131 -0.178869 0.510488

3 6 0 -0.779676 0.600645 0.397701

4 6 0 -2.047587 0.029418 -0.077181

5 6 0 -2.216907 -1.442490 -0.135855

6 6 0 -1.052494 -2.184758 -0.028332

7 1 0 1.027873 -2.250705 0.338849

8 1 0 -0.786826 1.673533 0.535285

9 1 0 -1.958213 0.205730 -1.189810

10 1 0 -1.082401 -3.263403 -0.153425

11 6 0 -3.570752 -2.004491 -0.441508

12 6 0 -4.656639 -0.931593 -0.544940

13 1 0 -3.808354 -2.729200 0.351453

14 1 0 -3.507026 -2.605910 -1.357326

15 1 0 -5.599099 -0.931056 0.065158

16 6 0 -4.393245 0.395451 0.057917

17 8 0 -5.356026 1.060627 0.364550

18 8 0 -3.140551 0.789460 0.421073

19 7 0 1.601534 0.315241 0.864128

20 6 0 1.778118 1.705262 1.302790

21 6 0 2.390539 2.645568 0.267331

22 1 0 2.445513 1.698178 2.173193

23 1 0 0.826489 2.102859 1.659338

24 1 0 2.490841 3.646066 0.692050

25 1 0 3.372240 2.306140 -0.066031

26 6 0 2.786584 -0.553416 0.902687

27 6 0 3.356771 -0.852866 -0.490371

28 1 0 2.565399 -1.487538 1.430768

29 1 0 3.547785 -0.049088 1.500774

30 1 0 2.611987 -1.286512 -1.161116

31 1 0 3.757908 0.045037 -0.961548

32 17 0 4.702362 -2.042398 -0.346325

33 17 0 1.363785 2.801156 -1.219387

34 1 0 -5.102694 -0.832153 -1.538622

---------------------------------------------------------------------

**TS9**

**
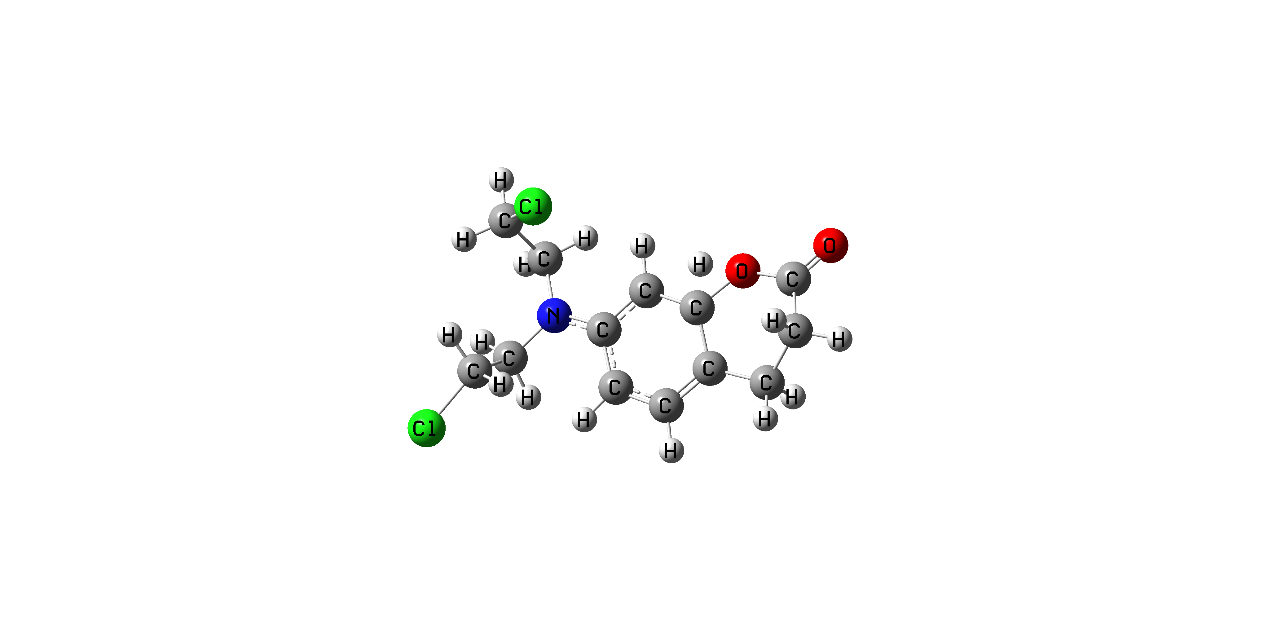
**

Zero-point correction= 0.268610 (Hartree/Particle)

Thermal correction to Energy= 0.286185

Thermal correction to Enthalpy= 0.287129

Thermal correction to Gibbs Free Energy= 0.220361

Sum of electronic and zero-point Energies= -1630.030769

Sum of electronic and thermal Energies= -1630.013194

Sum of electronic and thermal Enthalpies= -1630.012250

Sum of electronic and thermal Free Energies= -1630.079017

---------------------------------------------------------------------

Center Atomic Atomic Coordinates (Angstroms)

Number Number Type X Y Z

---------------------------------------------------------------------

1 6 0 0.192121 -1.604138 0.267910

2 6 0 0.373795 -0.199313 0.522785

3 6 0 -0.784484 0.574790 0.428537

4 6 0 -2.055917 -0.010702 -0.015319

5 6 0 -2.205434 -1.474912 -0.078639

6 6 0 -1.033895 -2.210875 -0.021744

7 1 0 1.054881 -2.258710 0.303023

8 1 0 -0.792037 1.650279 0.543510

9 1 0 -2.409436 0.964575 -0.670676

10 1 0 -1.059068 -3.287486 -0.161819

11 6 0 -3.585080 -2.003150 -0.272805

12 6 0 -4.534388 -0.906883 -0.794348

13 1 0 -3.941119 -2.351237 0.711015

14 1 0 -3.589473 -2.882529 -0.924912

15 1 0 -5.573211 -1.238438 -0.779902

16 6 0 -4.450372 0.364207 0.027270

17 8 0 -5.365575 1.055161 0.362491

18 8 0 -3.163900 0.738588 0.427872

19 7 0 1.603871 0.307975 0.863331

20 6 0 1.769556 1.695969 1.312198

21 6 0 2.364972 2.650948 0.280117

22 1 0 2.443714 1.688792 2.177467

23 1 0 0.816571 2.080976 1.679009

24 1 0 2.457416 3.649120 0.712037

25 1 0 3.347965 2.324597 -0.062321

26 6 0 2.797531 -0.549118 0.887041

27 6 0 3.363145 -0.828703 -0.512041

28 1 0 2.586960 -1.490705 1.405983

29 1 0 3.557002 -0.043937 1.486704

30 1 0 2.618010 -1.259303 -1.184187

31 1 0 3.756516 0.077141 -0.974491

32 17 0 4.717220 -2.010974 -0.388029

33 17 0 1.327082 2.807602 -1.198774

34 1 0 -4.288407 -0.660353 -1.836769

---------------------------------------------------------------------

**TS11**


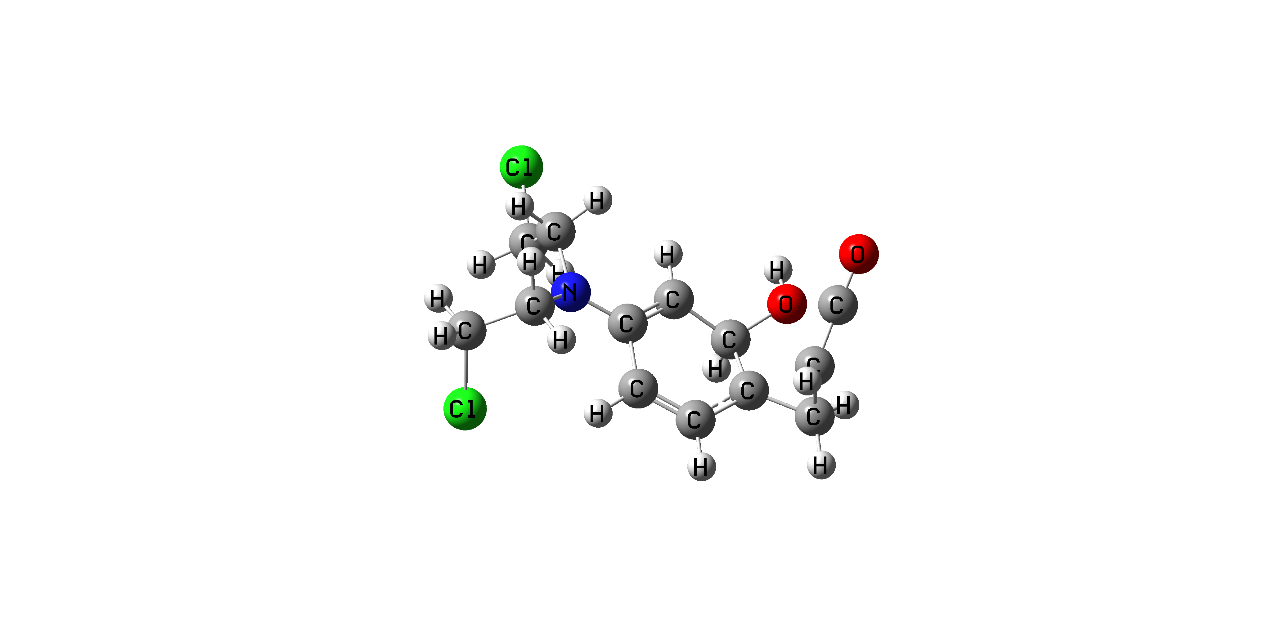


Zero-point correction= 0.270613 (Hartree/Particle)

Thermal correction to Energy= 0.288811

Thermal correction to Enthalpy= 0.289755

Thermal correction to Gibbs Free Energy= 0.221633

Sum of electronic and zero-point Energies= -1630.042944

Sum of electronic and thermal Energies= -1630.024747

Sum of electronic and thermal Enthalpies= -1630.023803

Sum of electronic and thermal Free Energies= -1630.091924

---------------------------------------------------------------------

Center Atomic Atomic Coordinates (Angstroms)

Number Number Type X Y Z

---------------------------------------------------------------------

1 6 0 0.392196 -1.640119 -0.216194

2 6 0 -0.118604 -0.298647 0.094127

3 6 0 0.667520 0.764268 -0.270044

4 6 0 1.947031 0.554982 -1.026871

5 6 0 2.594631 -0.781865 -0.782975

6 6 0 1.674122 -1.880958 -0.596942

7 1 0 0.335178 1.791165 -0.178141

8 1 0 1.709912 0.514183 -2.113167

9 1 0 2.016938 -2.898059 -0.762605

10 6 0 4.034165 -0.946812 -1.020687

11 6 0 3.920097 -0.724612 0.486895

12 1 0 4.539749 -0.155290 -1.565551

13 1 0 4.389038 -1.946084 -1.264133

14 1 0 3.906088 -1.579297 1.157393

15 6 0 4.144507 0.460788 1.120496

16 8 0 2.934711 1.554639 -0.817142

17 8 0 4.374070 1.375196 1.775823

18 1 0 2.569743 2.441594 -0.945394

19 7 0 -1.373456 -0.181332 0.668305

20 6 0 -1.963591 -1.243534 1.488580

21 6 0 -3.274517 -1.811852 0.951734

22 1 0 -2.171168 -0.839296 2.489916

23 1 0 -1.242502 -2.049209 1.633943

24 1 0 -3.683468 -2.534826 1.659890

25 1 0 -4.018485 -1.033033 0.776003

26 6 0 -1.972735 1.148627 0.800176

27 6 0 -2.576939 1.637872 -0.521134

28 1 0 -1.236012 1.871599 1.172693

29 1 0 -2.758243 1.099485 1.557215

30 1 0 -1.860189 1.588074 -1.341286

31 17 0 -3.054472 -2.684401 -0.622468

32 17 0 -3.087000 3.363689 -0.360616

33 1 0 -3.460613 1.060051 -0.793941

34 1 0 -0.294307 -2.477133 -0.160955

---------------------------------------------------------------------

**h**


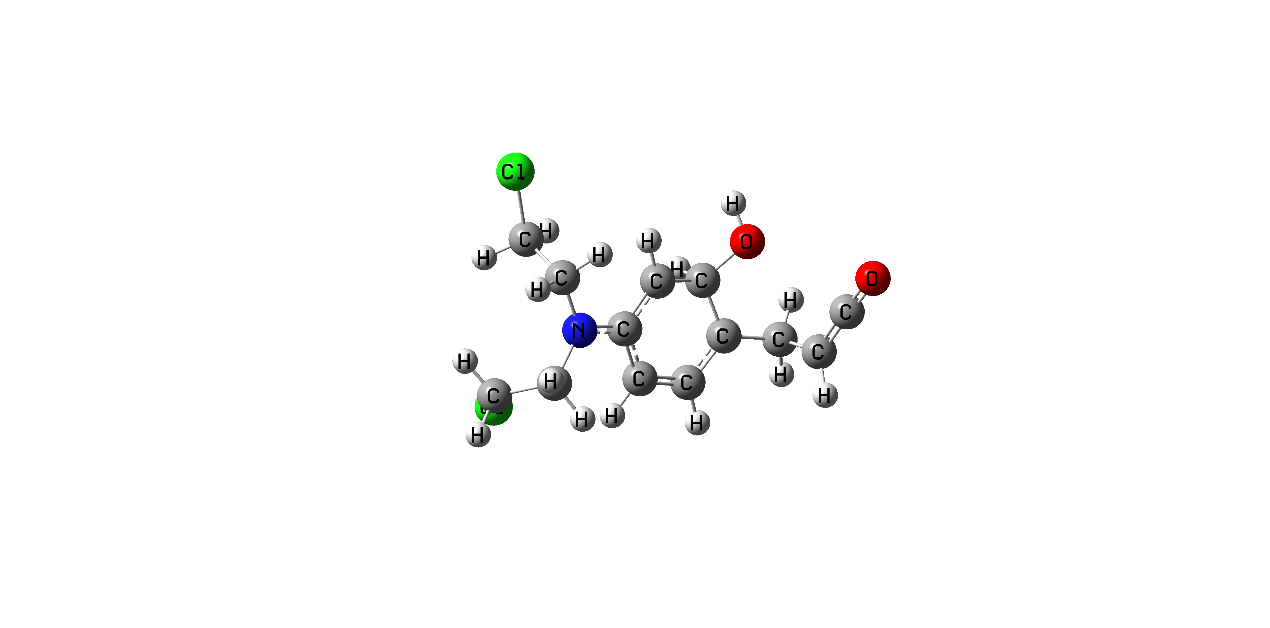


Zero-point correction= 0.270157 (Hartree/Particle)

Thermal correction to Energy= 0.289295

Thermal correction to Enthalpy= 0.290239

Thermal correction to Gibbs Free Energy= 0.219312

Sum of electronic and zero-point Energies= -1630.047527

Sum of electronic and thermal Energies= -1630.028389

Sum of electronic and thermal Enthalpies= -1630.027445

Sum of electronic and thermal Free Energies= -1630.098372

---------------------------------------------------------------------

Center Atomic Atomic Coordinates (Angstroms)

Number Number Type X Y Z

---------------------------------------------------------------------

1 6 0 0.343681 -1.628237 -0.220939

2 6 0 -0.192895 -0.311577 0.069821

3 6 0 0.583637 0.757875 -0.331384

4 6 0 1.887243 0.550321 -1.027049

5 6 0 2.519432 -0.786340 -0.874441

6 6 0 1.643367 -1.856897 -0.627944

7 1 0 0.241740 1.783610 -0.269371

8 1 0 1.611633 0.464299 -2.115182

9 1 0 1.996885 -2.879079 -0.726451

10 6 0 3.997248 -0.918127 -0.982465

11 6 0 4.262638 -0.743840 0.507230

12 1 0 4.464008 -0.147433 -1.593521

13 1 0 4.321233 -1.907390 -1.308425

14 1 0 4.286784 -1.616657 1.152813

15 6 0 4.418275 0.427367 1.135824

16 8 0 2.842150 1.570179 -0.862367

17 8 0 4.587034 1.400943 1.740274

18 1 0 2.488852 2.432015 -1.124449

19 7 0 -1.425293 -0.182289 0.677606

20 6 0 -2.002296 -1.246048 1.506884

21 6 0 -3.298572 -1.844286 0.969262

22 1 0 -2.220070 -0.830448 2.500416

23 1 0 -1.265792 -2.035767 1.666827

24 1 0 -3.690182 -2.579692 1.674377

25 1 0 -4.060433 -1.082645 0.795439

26 6 0 -2.010612 1.154062 0.820054

27 6 0 -2.653244 1.644225 -0.482854

28 1 0 -1.256401 1.871652 1.167000

29 1 0 -2.770791 1.113942 1.602656

30 1 0 -1.972608 1.566532 -1.331754

31 17 0 -3.051466 -2.702566 -0.608049

32 17 0 -3.110039 3.382704 -0.321361

33 1 0 -3.560828 1.085824 -0.714848

34 1 0 -0.312274 -2.485047 -0.114058

---------------------------------------------------------------------

**TS12**


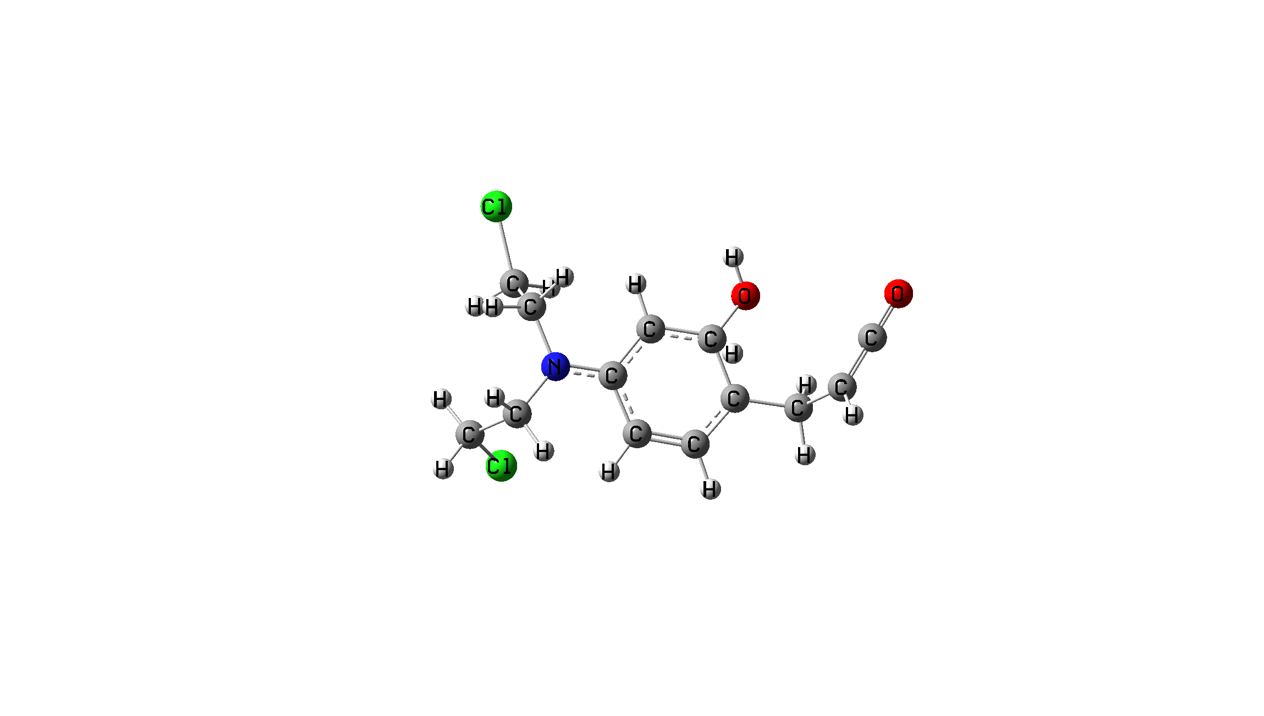


Zero-point correction= 0.268028 (Hartree/Particle)

Thermal correction to Energy= 0.286744

Thermal correction to Enthalpy= 0.287688

Thermal correction to Gibbs Free Energy= 0.217521

Sum of electronic and zero-point Energies= -1630.043664

Sum of electronic and thermal Energies= -1630.024949

Sum of electronic and thermal Enthalpies= -1630.024004

Sum of electronic and thermal Free Energies= -1630.094171

---------------------------------------------------------------------

Center Atomic Atomic Coordinates (Angstroms)

Number Number Type X Y Z

---------------------------------------------------------------------

1 6 0 -0.240594 -1.739415 -0.717035

2 6 0 0.371442 -0.416332 -0.764590

3 6 0 -0.481776 0.719602 -0.584213

4 6 0 -1.832560 0.571225 -0.409561

5 6 0 -2.528017 -0.753653 -0.366439

6 6 0 -1.566166 -1.883539 -0.534254

7 1 0 -0.075634 1.722797 -0.602608

8 1 0 -1.987694 -2.884098 -0.495898

9 6 0 -3.411580 -0.945631 0.917333

10 6 0 -4.827280 -0.411254 0.849898

11 1 0 -2.868910 -0.529490 1.776469

12 1 0 -3.482993 -2.022572 1.098654

13 1 0 -5.555332 -0.821018 1.545339

14 6 0 -5.297658 0.531112 0.054641

15 8 0 -5.747412 1.345029 -0.655484

16 7 0 1.687455 -0.261414 -0.973311

17 6 0 2.599621 -1.373144 -1.314304

18 6 0 3.568510 -1.764864 -0.200124

19 1 0 3.190311 -1.048581 -2.178736

20 1 0 2.023892 -2.236443 -1.637226

21 1 0 4.189481 -2.593165 -0.545959

22 1 0 4.222570 -0.941881 0.091307

23 6 0 2.323371 1.076184 -0.919948

24 6 0 2.517232 1.587171 0.514644

25 1 0 1.733862 1.785906 -1.504881

26 1 0 3.292677 0.999734 -1.414231

27 1 0 1.582571 1.604040 1.075702

28 17 0 2.707646 -2.316795 1.288492

29 17 0 3.144531 3.273214 0.449614

30 1 0 3.240618 0.986844 1.066095

31 1 0 -2.710091 0.246478 -1.312047

32 1 0 0.375573 -2.623289 -0.786256

33 8 0 -2.152438 1.231425 0.817921

34 1 0 -2.438565 2.129118 0.633860

---------------------------------------------------------------------

**h1**

**
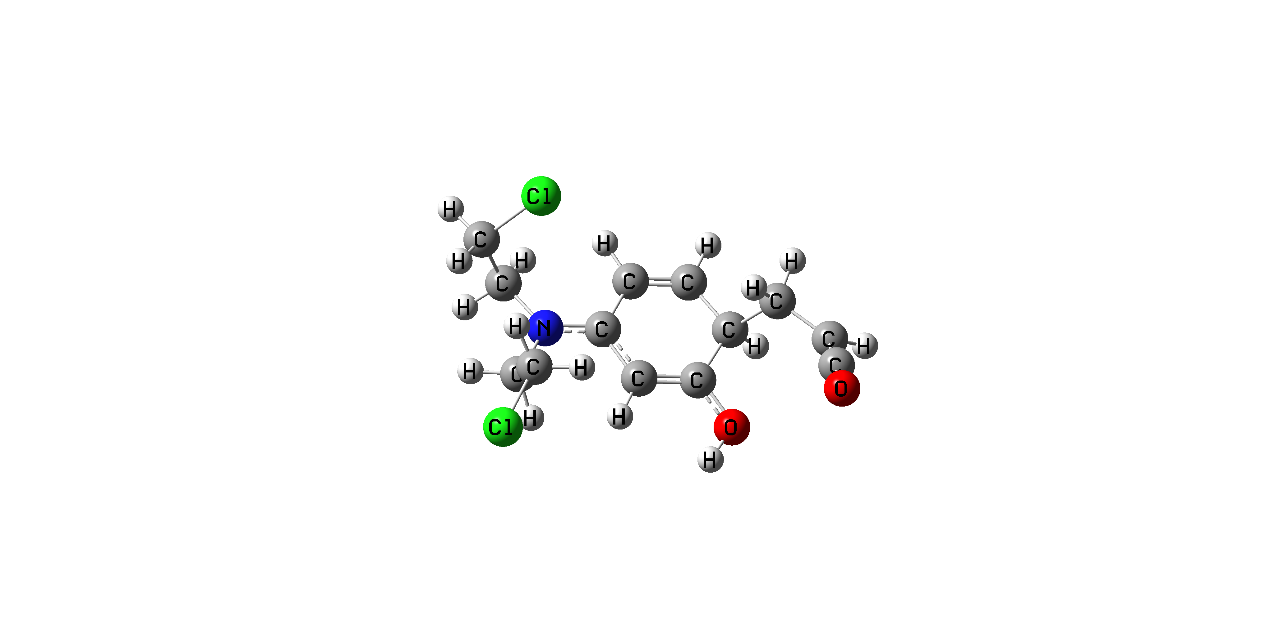
**

Zero-point correction= 0.273125 (Hartree/Particle)

Thermal correction to Energy= 0.291797

Thermal correction to Enthalpy= 0.292741

Thermal correction to Gibbs Free Energy= 0.222067

Sum of electronic and zero-point Energies= -1630.100051

Sum of electronic and thermal Energies= -1630.081379

Sum of electronic and thermal Enthalpies= -1630.080434

Sum of electronic and thermal Free Energies= -1630.151108

---------------------------------------------------------------------

Center Atomic Atomic Coordinates (Angstroms)

Number Number Type X Y Z

---------------------------------------------------------------------

1 6 0 0.117750 -1.730662 0.356702

2 6 0 -0.433311 -0.401121 0.499664

3 6 0 0.438563 0.667527 0.181986

4 6 0 1.778873 0.450722 -0.196057

5 6 0 2.346313 -0.887046 -0.237372

6 6 0 1.417103 -1.959046 0.016201

7 1 0 0.093124 1.693215 0.175432

8 1 0 1.780548 -2.977762 -0.071971

9 6 0 3.785153 -1.123918 -0.682070

10 6 0 4.775195 -0.661339 0.371991

11 1 0 3.961910 -0.614744 -1.633179

12 1 0 3.917385 -2.194191 -0.864029

13 1 0 5.017360 -1.296054 1.218935

14 6 0 5.457412 0.468119 0.262769

15 8 0 6.051015 1.467661 0.176947

16 7 0 -1.725500 -0.196399 0.887892

17 6 0 -2.604319 -1.272666 1.377141

18 6 0 -3.675391 -1.742523 0.394411

19 1 0 -3.119292 -0.894536 2.268369

20 1 0 -2.001673 -2.116904 1.710114

21 1 0 -4.280825 -2.520540 0.863130

22 1 0 -4.333056 -0.930210 0.081660

23 6 0 -2.278143 1.167723 0.928316

24 6 0 -2.673547 1.696634 -0.458003

25 1 0 -1.556698 1.843421 1.398338

26 1 0 -3.155548 1.158448 1.577418

27 1 0 -1.864954 1.601378 -1.183698

28 17 0 -2.964849 -2.452064 -1.111830

29 17 0 -3.081346 3.449355 -0.334810

30 1 0 -3.551377 1.183428 -0.851318

31 1 0 2.454824 -0.326715 0.848883

32 1 0 -0.520020 -2.590020 0.503490

33 8 0 2.577790 1.415043 -0.690900

34 1 0 2.281786 2.306563 -0.446383

---------------------------------------------------------------------

**TS13**


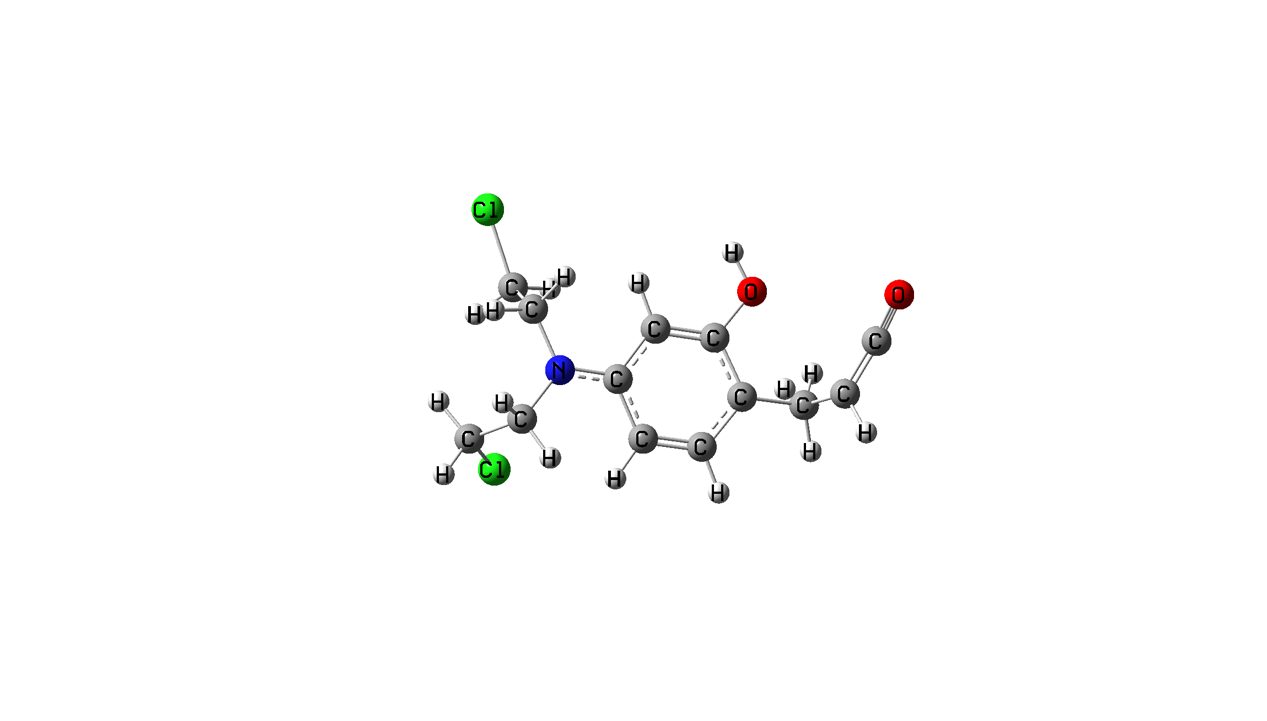


Zero-point correction= 0.267495 (Hartree/Particle)

Thermal correction to Energy= 0.285757

Thermal correction to Enthalpy= 0.286701

Thermal correction to Gibbs Free Energy= 0.217938

Sum of electronic and zero-point Energies= -1630.056474

Sum of electronic and thermal Energies= -1630.038213

Sum of electronic and thermal Enthalpies= -1630.037268

Sum of electronic and thermal Free Energies= -1630.106031

---------------------------------------------------------------------

Center Atomic Atomic Coordinates (Angstroms)

Number Number Type X Y Z

---------------------------------------------------------------------

1 6 0 0.213833 -1.726264 0.320572

2 6 0 -0.345721 -0.409036 0.458417

3 6 0 0.487883 0.695645 0.120566

4 6 0 1.791157 0.500800 -0.294078

5 6 0 2.396698 -0.802963 -0.329167

6 6 0 1.507275 -1.902585 -0.087107

7 1 0 0.115189 1.710902 0.173509

8 1 0 1.882997 -2.913308 -0.220725

9 6 0 3.765808 -0.982924 -1.002213

10 6 0 4.671364 -0.730697 0.241161

11 1 0 3.945500 -0.284326 -1.820437

12 1 0 3.915644 -1.999887 -1.368051

13 1 0 5.138549 -1.587201 0.726550

14 6 0 5.369391 0.429820 0.376478

15 8 0 5.870407 1.450113 0.556953

16 7 0 -1.622353 -0.212387 0.894172

17 6 0 -2.459295 -1.288702 1.442648

18 6 0 -3.623907 -1.724996 0.555993

19 1 0 -2.886446 -0.926248 2.386567

20 1 0 -1.837580 -2.145140 1.696433

21 1 0 -4.187922 -2.510442 1.062491

22 1 0 -4.302953 -0.900956 0.331987

23 6 0 -2.216302 1.133627 0.916156

24 6 0 -2.659225 1.612807 -0.473081

25 1 0 -1.513950 1.847499 1.356893

26 1 0 -3.081312 1.107587 1.581579

27 1 0 -1.845535 1.582643 -1.198203

28 17 0 -3.073252 -2.398566 -1.029915

29 17 0 -3.217156 3.325584 -0.361613

30 1 0 -3.490032 1.022404 -0.859702

31 8 0 2.619267 1.517564 -0.635122

32 1 0 2.191723 2.381981 -0.540831

33 1 0 3.338609 -0.625750 0.759428

34 1 0 -0.400224 -2.600959 0.476574

---------------------------------------------------------------------

**g1**


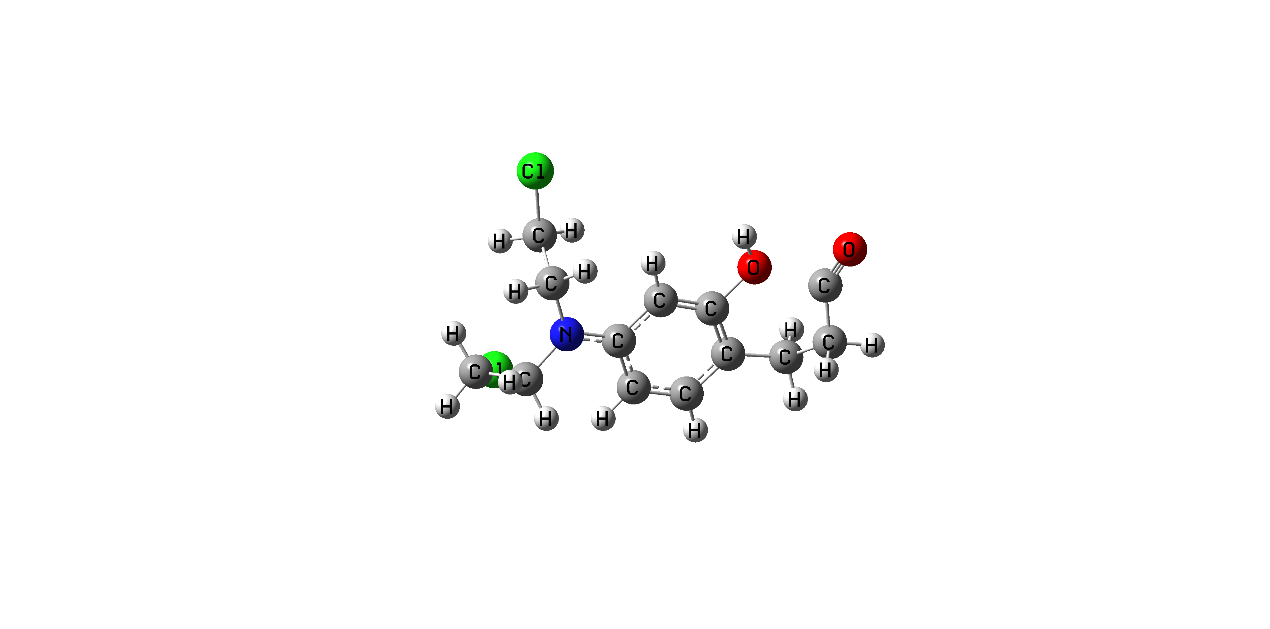


Zero-point correction= 0.273179 (Hartree/Particle)

Thermal correction to Energy= 0.291442

Thermal correction to Enthalpy= 0.292386

Thermal correction to Gibbs Free Energy= 0.223729

Sum of electronic and zero-point Energies= -1630.098702

Sum of electronic and thermal Energies= -1630.080439

Sum of electronic and thermal Enthalpies= -1630.079495

Sum of electronic and thermal Free Energies= -1630.148152

---------------------------------------------------------------------

Center Atomic Atomic Coordinates (Angstroms)

Number Number Type X Y Z

---------------------------------------------------------------------

1 6 0 0.209381 -1.721374 0.322126

2 6 0 -0.343348 -0.411768 0.457633

3 6 0 0.486222 0.692131 0.121187

4 6 0 1.793656 0.487148 -0.292086

5 6 0 2.371644 -0.807872 -0.365964

6 6 0 1.514204 -1.897470 -0.089210

7 1 0 0.116262 1.708984 0.172367

8 1 0 1.882943 -2.911348 -0.224455

9 6 0 3.750471 -0.985117 -1.004274

10 6 0 4.644695 -0.738366 0.257816

11 1 0 3.948106 -0.282097 -1.815035

12 1 0 3.919383 -2.000837 -1.364516

13 1 0 5.180447 -1.596112 0.674404

14 6 0 5.377332 0.439919 0.374035

15 8 0 5.869179 1.448624 0.550975

16 7 0 -1.628665 -0.210955 0.895874

17 6 0 -2.459791 -1.286126 1.443813

18 6 0 -3.625453 -1.724721 0.559044

19 1 0 -2.890634 -0.928666 2.389140

20 1 0 -1.837657 -2.143277 1.696569

21 1 0 -4.190954 -2.510267 1.064021

22 1 0 -4.303470 -0.900045 0.333738

23 6 0 -2.216655 1.132349 0.916797

24 6 0 -2.660414 1.612317 -0.471895

25 1 0 -1.515348 1.849559 1.356184

26 1 0 -3.082614 1.110611 1.582143

27 1 0 -1.847458 1.582128 -1.197803

28 17 0 -3.075896 -2.398572 -1.027901

29 17 0 -3.218310 3.326271 -0.361346

30 1 0 -3.492013 1.022917 -0.858355

31 8 0 2.622493 1.519610 -0.637692

32 1 0 2.182342 2.376963 -0.548178

33 1 0 3.749355 -0.566488 0.927693

34 1 0 -0.401566 -2.598411 0.477758

---------------------------------------------------------------------

**TS10**


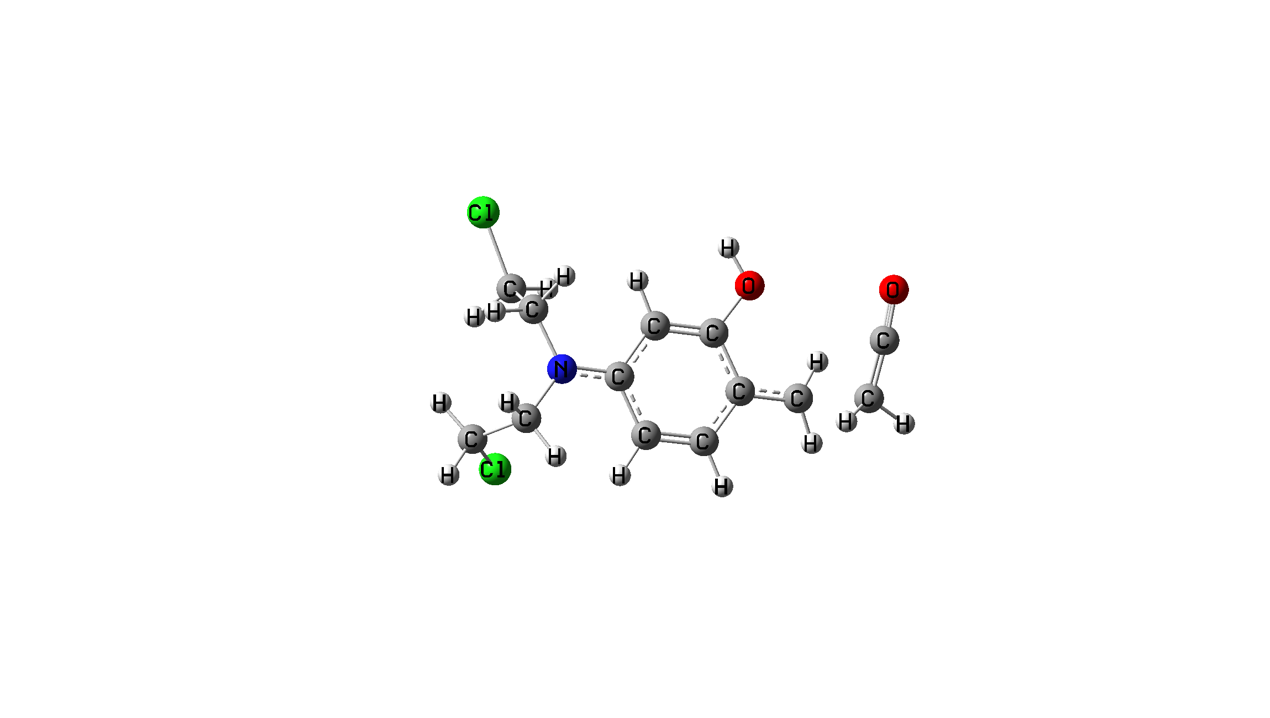


Zero-point correction= 0.269821 (Hartree/Particle)

Thermal correction to Energy= 0.288722

Thermal correction to Enthalpy= 0.289666

Thermal correction to Gibbs Free Energy= 0.218254

Sum of electronic and zero-point Energies= -1630.090861

Sum of electronic and thermal Energies= -1630.071960

Sum of electronic and thermal Enthalpies= -1630.071016

Sum of electronic and thermal Free Energies= -1630.142428

---------------------------------------------------------------------

Center Atomic Atomic Coordinates (Angstroms)

Number Number Type X Y Z

---------------------------------------------------------------------

1 6 0 0.243250 -1.693179 0.276089

2 6 0 -0.346816 -0.391488 0.420939

3 6 0 0.446644 0.733877 0.052633

4 6 0 1.738182 0.574911 -0.412163

5 6 0 2.325139 -0.719503 -0.579252

6 6 0 1.521893 -1.832712 -0.192741

7 1 0 0.059388 1.740324 0.149842

8 1 0 1.928579 -2.832626 -0.317071

9 6 0 3.608541 -0.877158 -1.134125

10 6 0 5.158935 -0.868202 0.350109

11 1 0 4.032621 -0.065382 -1.712393

12 1 0 3.907396 -1.874396 -1.441099

13 1 0 5.932459 -1.451924 -0.147002

14 6 0 5.518419 0.418104 0.591441

15 8 0 5.744321 1.541384 0.700457

16 7 0 -1.614194 -0.223109 0.896491

17 6 0 -2.405286 -1.315517 1.479145

18 6 0 -3.594510 -1.775844 0.638292

19 1 0 -2.802014 -0.961331 2.439484

20 1 0 -1.757080 -2.159003 1.707340

21 1 0 -4.123137 -2.571678 1.166321

22 1 0 -4.297752 -0.965494 0.440486

23 6 0 -2.244637 1.106283 0.926952

24 6 0 -2.730222 1.566451 -0.454406

25 1 0 -1.555114 1.842216 1.350840

26 1 0 -3.094593 1.057836 1.610209

27 1 0 -1.928251 1.567651 -1.192921

28 17 0 -3.091306 -2.439779 -0.967018

29 17 0 -3.351945 3.256656 -0.333209

30 1 0 -3.544026 0.943822 -0.826535

31 8 0 2.536742 1.624243 -0.723806

32 1 0 2.068706 2.468374 -0.646342

33 1 0 4.654251 -1.360773 1.177784

34 1 0 -0.332496 -2.583918 0.478817

---------------------------------------------------------------------

**p2’**


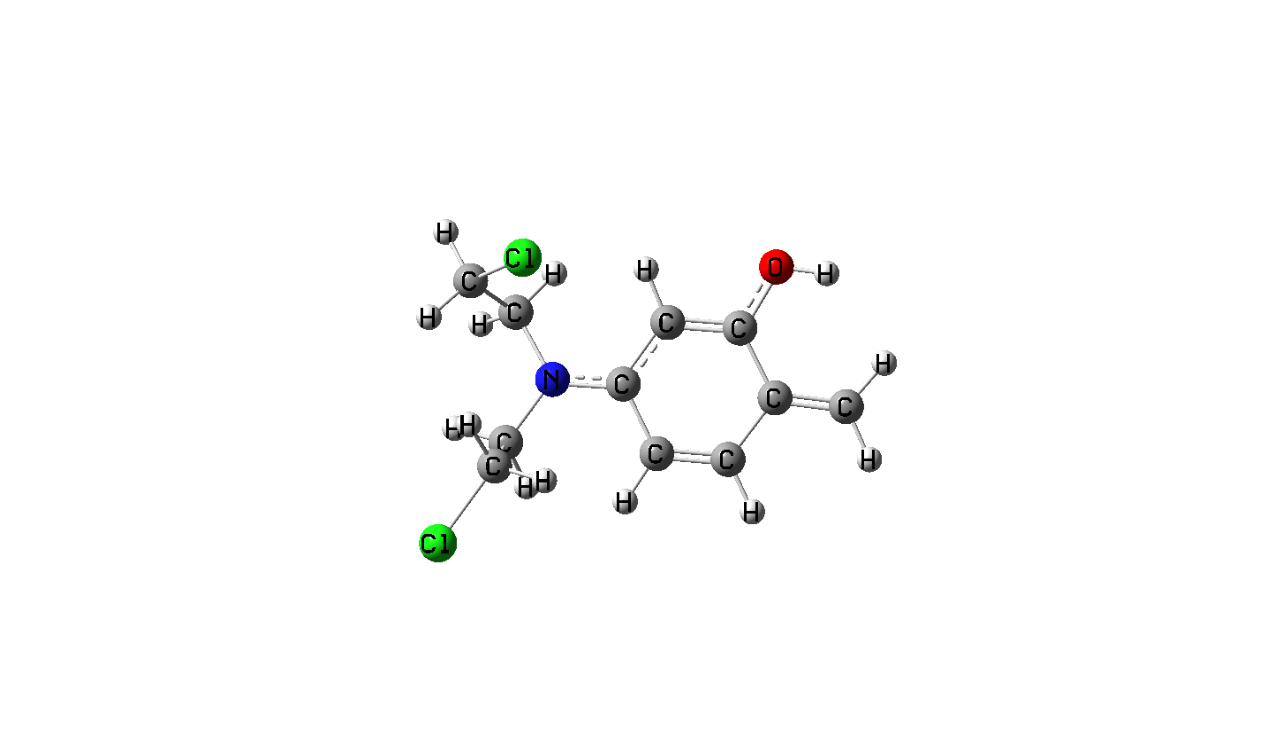


Zero-point correction= 0.235521 (Hartree/Particle)

Thermal correction to Energy= 0.250814

Thermal correction to Enthalpy= 0.251758

Thermal correction to Gibbs Free Energy= 0.190788

Sum of electronic and zero-point Energies= -1477.511706

Sum of electronic and thermal Energies= -1477.496413

Sum of electronic and thermal Enthalpies= -1477.495469

Sum of electronic and thermal Free Energies= -1477.556439

---------------------------------------------------------------------

Center Atomic Atomic Coordinates (Angstroms)

Number Number Type X Y Z

---------------------------------------------------------------------

1 6 0 -0.737550 -1.597557 0.255126

2 6 0 -0.498241 -0.220146 0.491489

3 6 0 -1.612444 0.649492 0.348114

4 6 0 -2.871439 0.169251 0.018553

5 6 0 -3.108469 -1.198500 -0.208670

6 6 0 -2.008326 -2.063392 -0.078819

7 1 0 0.057145 -2.324997 0.355791

8 1 0 -1.494722 1.717141 0.471264

9 1 0 -2.145828 -3.138098 -0.192571

10 6 0 -4.469478 -1.722091 -0.608556

11 1 0 -5.278072 -1.229978 -0.060601

12 1 0 -4.544120 -2.791742 -0.385363

13 7 0 0.758041 0.242368 0.845340

14 6 0 0.983181 1.591380 1.361776

15 6 0 1.736470 2.538064 0.428559

16 1 0 1.582401 1.512388 2.279846

17 1 0 0.034143 2.037264 1.658982

18 1 0 1.880201 3.503307 0.917977

19 1 0 2.712355 2.141723 0.143909

20 6 0 1.905245 -0.666507 0.879666

21 6 0 2.457382 -0.963529 -0.520063

22 1 0 1.647884 -1.601865 1.388304

23 1 0 2.689735 -0.201601 1.481115

24 1 0 1.690579 -1.366707 -1.182193

25 1 0 2.885446 -0.073185 -0.981458

26 17 0 3.772845 -2.200737 -0.416302

27 17 0 0.842001 2.859424 -1.116767

28 8 0 -3.946923 1.107002 -0.075556

29 1 0 -4.558421 0.967292 0.651185

---------------------------------------------------------------------
